# Supplementary material for: Screening for functional regulatory variants in open chromatin using GenIE-ATAC
Source: Nucleic Acids Res. 2023 May 1;51(11):e64. doi: 10.1093/nar/gkad332 (PMC10287956; doi:10.1093/nar/gkad332)
Supplement: gkad332_Supplemental_Files [file gkad332_supplemental_files.zip › Supplementary_Note.pdf]

Experiment summary

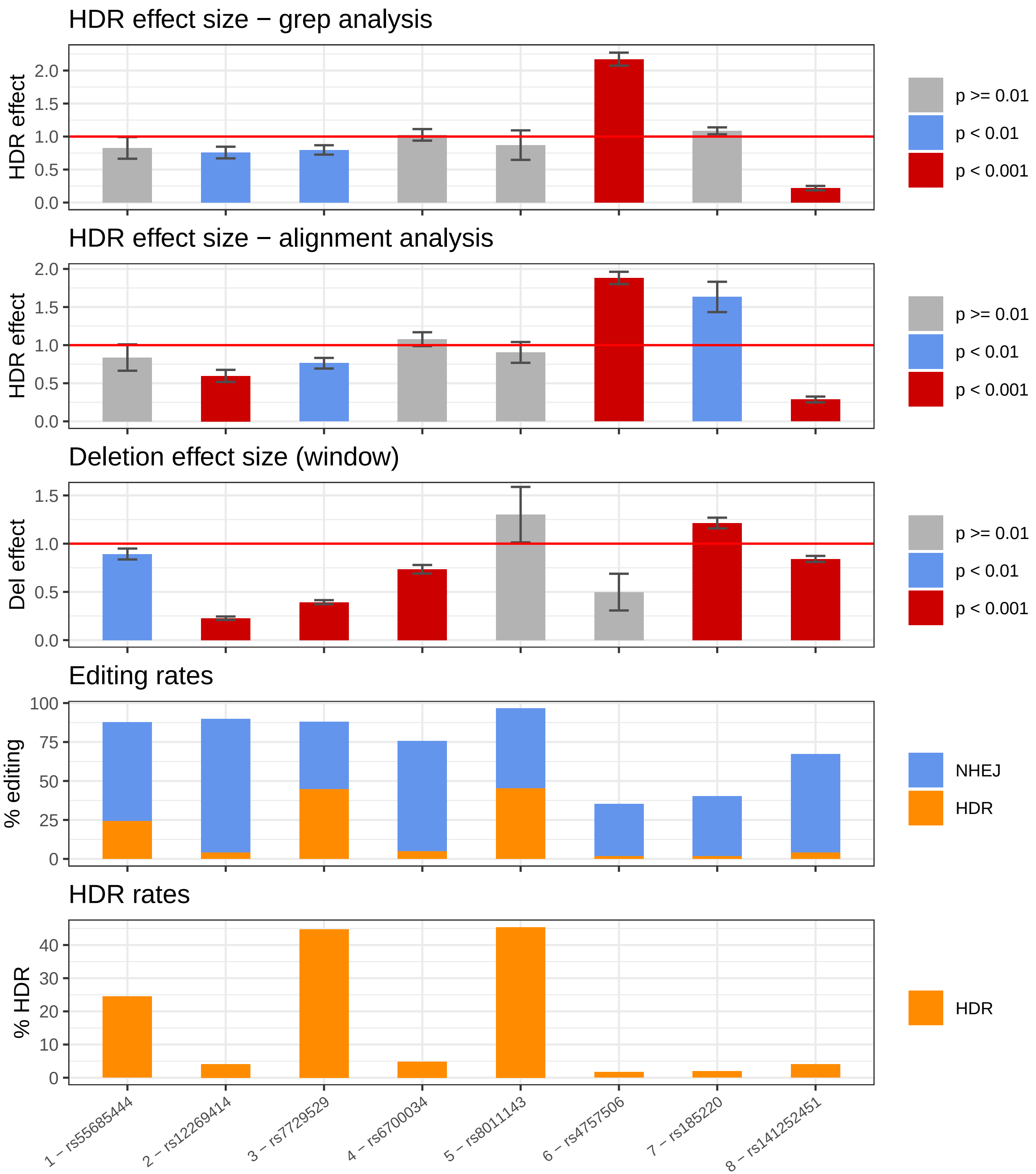

1 − rs55685444 grep summary

Mean HDR frac gDNA: 23%, ATAC: 6.7%
Mean WT frac gDNA: 9.1%, ATAC: 3.2%

ATAC:gDNA ratio (HDR/WT): 0.828
95% CI: (0.664, 0.993),   p = 0.0446

replicate	atac1.1	atac1.2	atac1.3	gDNA1.1	gDNA1.2	gDNA1.3
type	ATAC	ATAC	ATAC	gDNA	gDNA	gDNA
num_reads	72132	99745	92593	69808	81434	38719
HDR reads	5448	5972	6005	16214	18927	9156
WT reads	2432	3104	2750	6368	7352	3598
HDR_WT_ratio	2.24	1.92	2.18	2.55	2.57	2.54
HDR_frac	7.55%	5.99%	6.49%	23.23%	23.24%	23.65%
WT_frac	3.37%	3.11%	2.97%	9.12%	9.03%	9.29%

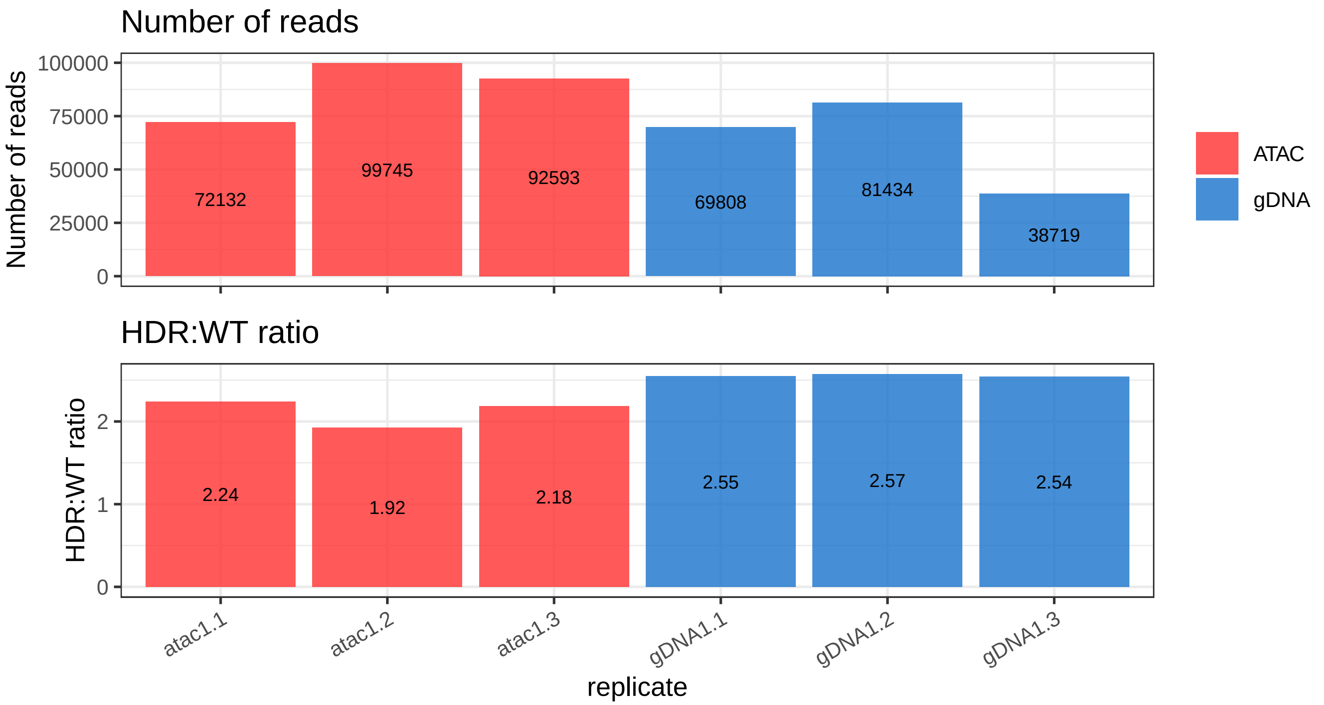

1 − rs55685444 analysis summary

Mean HDR frac gDNA: 25%, ATAC: 22%
Mean DEL frac gDNA: 63%, ATAC: 54%
Mean WT frac gDNA: 10%, ATAC: 11%

ATAC:gDNA ratio (HDR/WT): 0.837
95% CI: (0.664, 1.01),   p = 0.0515

ATAC:gDNA ratio (DEL/WT) [65−77]: 0.893
95% CI: (0.836, 0.949),   p = 0.00856
ATAC:gDNA ratio (DEL/WT) − Del 1: 1.47
95% CI: (0.882, 2.06),   p = 0.0373
ATAC:gDNA ratio (DEL/WT) − Del 2: 0.739
95% CI: (0.669, 0.809),   p = 0.00511

replicate	atac1.1	atac1.2	atac1.3	gDNA1.1	gDNA1.2	gDNA1.3
type	ATAC	ATAC	ATAC	gDNA	gDNA	gDNA
num_udps	678	715	651	2377	2536	1809
HDR_WT_ratio	2.17	1.86	2.15	2.47	2.48	2.43
DEL_WT_ratio	5.28	4.97	5.14	6.4	6.45	6.19
HDR_rate	22.42%	20.57%	22.53%	24.41%	24.43%	24.67%
DEL_rate	54.66%	54.90%	53.89%	63.32%	63.42%	62.86%
editing_rate	77.09%	75.47%	76.42%	87.73%	87.85%	87.53%
WT_rate	10.35%	11.04%	10.49%	9.89%	9.84%	10.16%
num_reads	72132	99745	92593	69808	81434	38719
HDR reads	5104	5609	5690	15081	17520	8387
WT reads	2357	3010	2648	6108	7057	3453
Deletion reads	12443	14973	13608	39112	45486	21374
excluded−insertion	2954	3179	3028	7715	9213	4508
excluded−minoverlap	53	88	75	0	0	0
excluded−mismatches	1	4	4	1	0	0
excluded−nonspanning	46361	69201	64235	322	500	209
excluded−mult.deletions	0	0	0	0	0	0

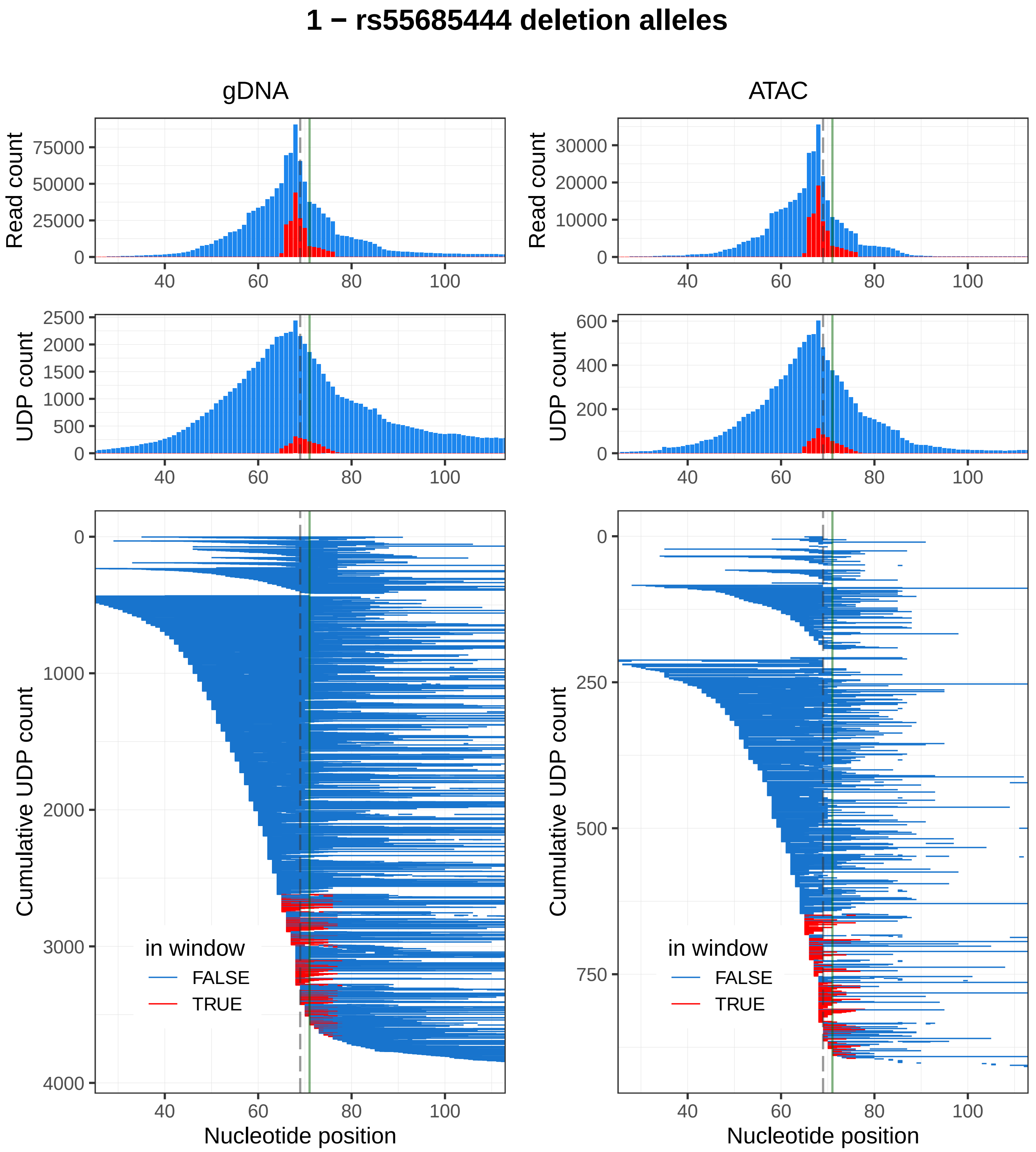
1 − rs55685444 deletion alleles
gDNA
ATAC
Read count
UDP count
Cumulative UDP count
Nucleotide position
in window
FALSE
TRUE
75000
50000
25000
0
30000
20000
10000
2500
2000
1500
1000
500
600
400
200
3000
4000
250
750
40
60
80
100

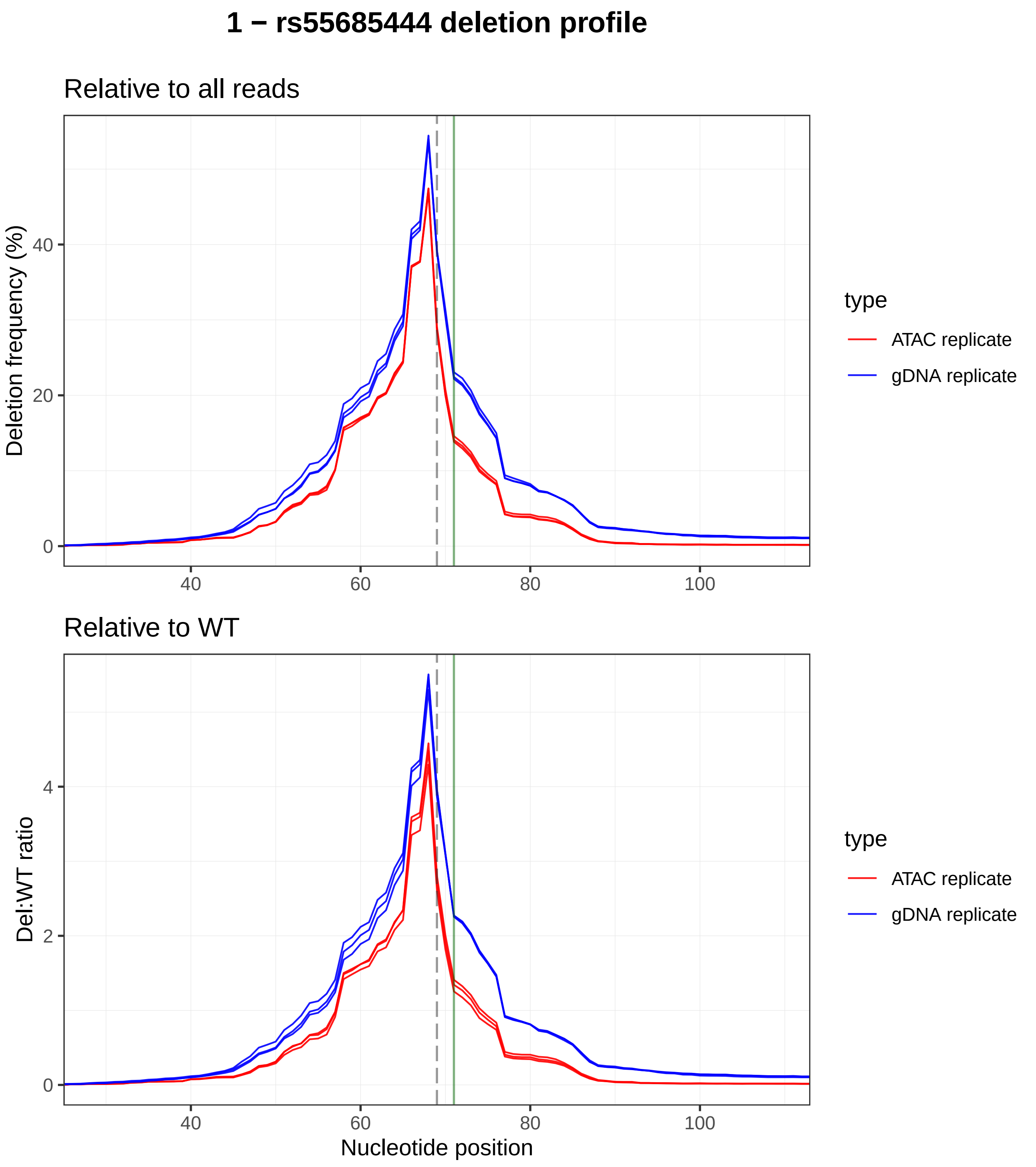
1 – rs55685444 deletion profile
Relative to all reads
Deletion frequency (%)
0
20
40
40
60
80
100
type
ATAC replicate
gDNA replicate
Relative to WT
Del:WT ratio
0
2
4
40
60
80
100
Nucleotide position
type
ATAC replicate
gDNA replicate

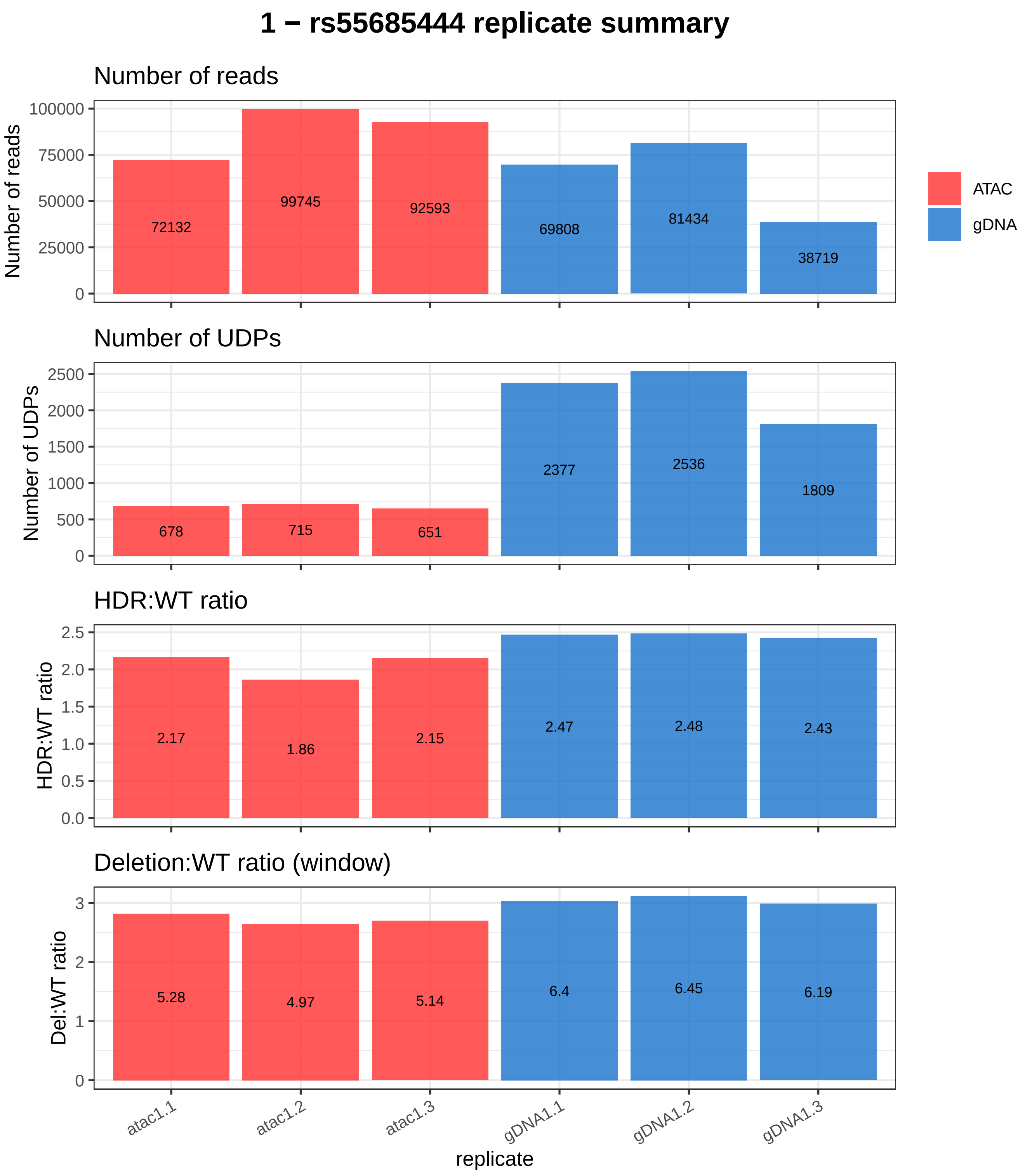
1 − rs55685444 replicate summary
Number of reads
Number of reads
100000
75000
50000
25000
0
72132
99745
92593
69808
81434
38719
ATAC
gDNA
Number of UDPs
Number of UDPs
2500
2000
1500
1000
500
0
678
715
651
2377
2536
1809
HDR:WT ratio
HDR:WT ratio
2.5
2.0
1.5
1.0
0.5
0.0
2.17
1.86
2.15
2.47
2.48
2.43
Deletion:WT ratio (window)
Del:WT ratio
3
2
1
0
5.28
4.97
5.14
6.4
6.45
6.19
atac1.1
atac1.2
atac1.3
gDNA1.1
gDNA1.2
gDNA1.3
replicate

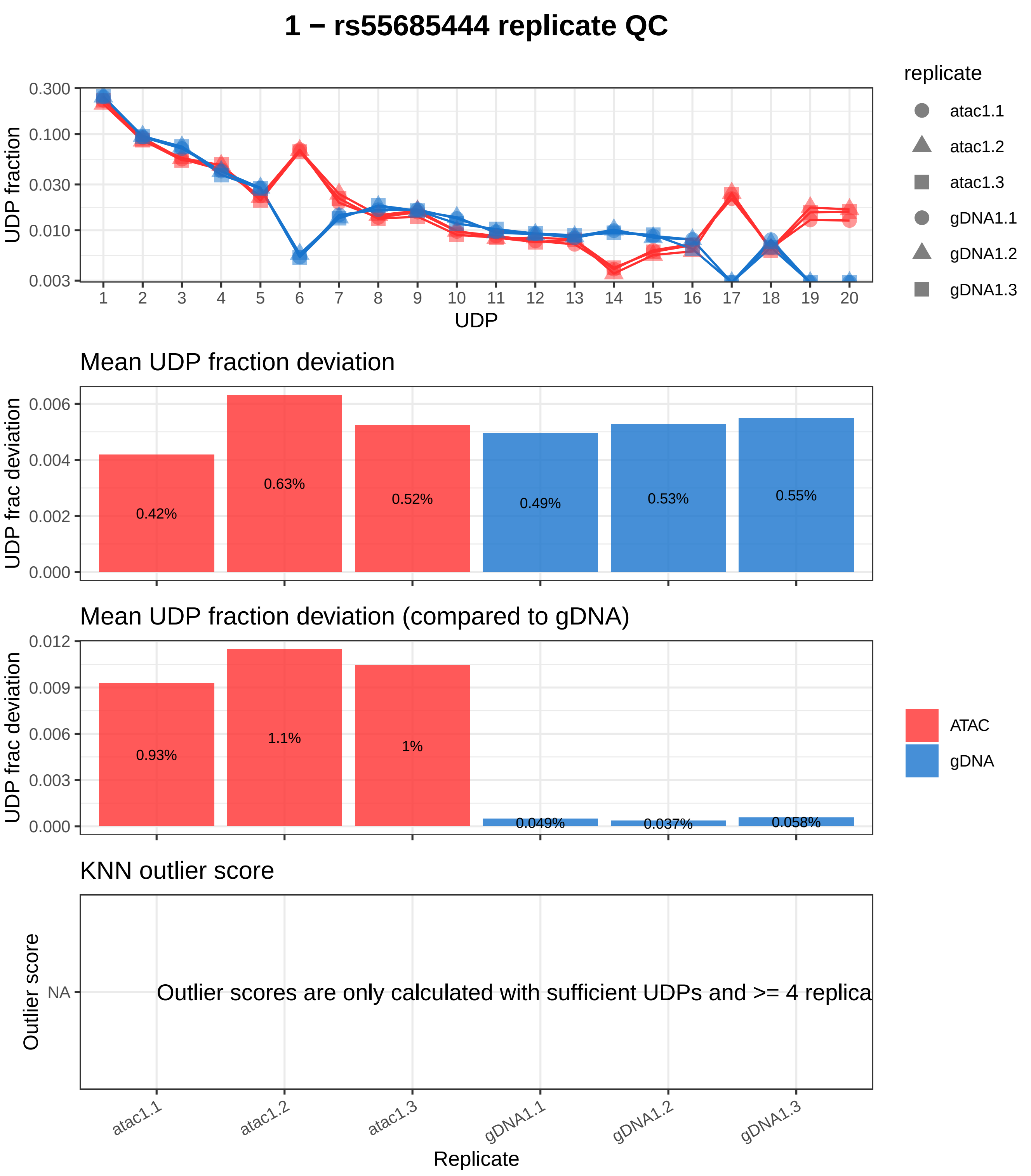
1 − rs55685444 replicate QC
UDP fraction
UDP
replicate
atac1.1
atac1.2
atac1.3
gDNA1.1
gDNA1.2
gDNA1.3
Mean UDP fraction deviation
UDP frac deviation
0.42%
0.63%
0.52%
0.49%
0.53%
0.55%
Mean UDP fraction deviation (compared to gDNA)
0.93%
1.1%
1%
0.049%
0.037%
0.058%
ATAC
gDNA
KNN outlier score
Outlier score
NA
Outlier scores are only calculated with sufficient UDPs and >= 4 replica
Replicate

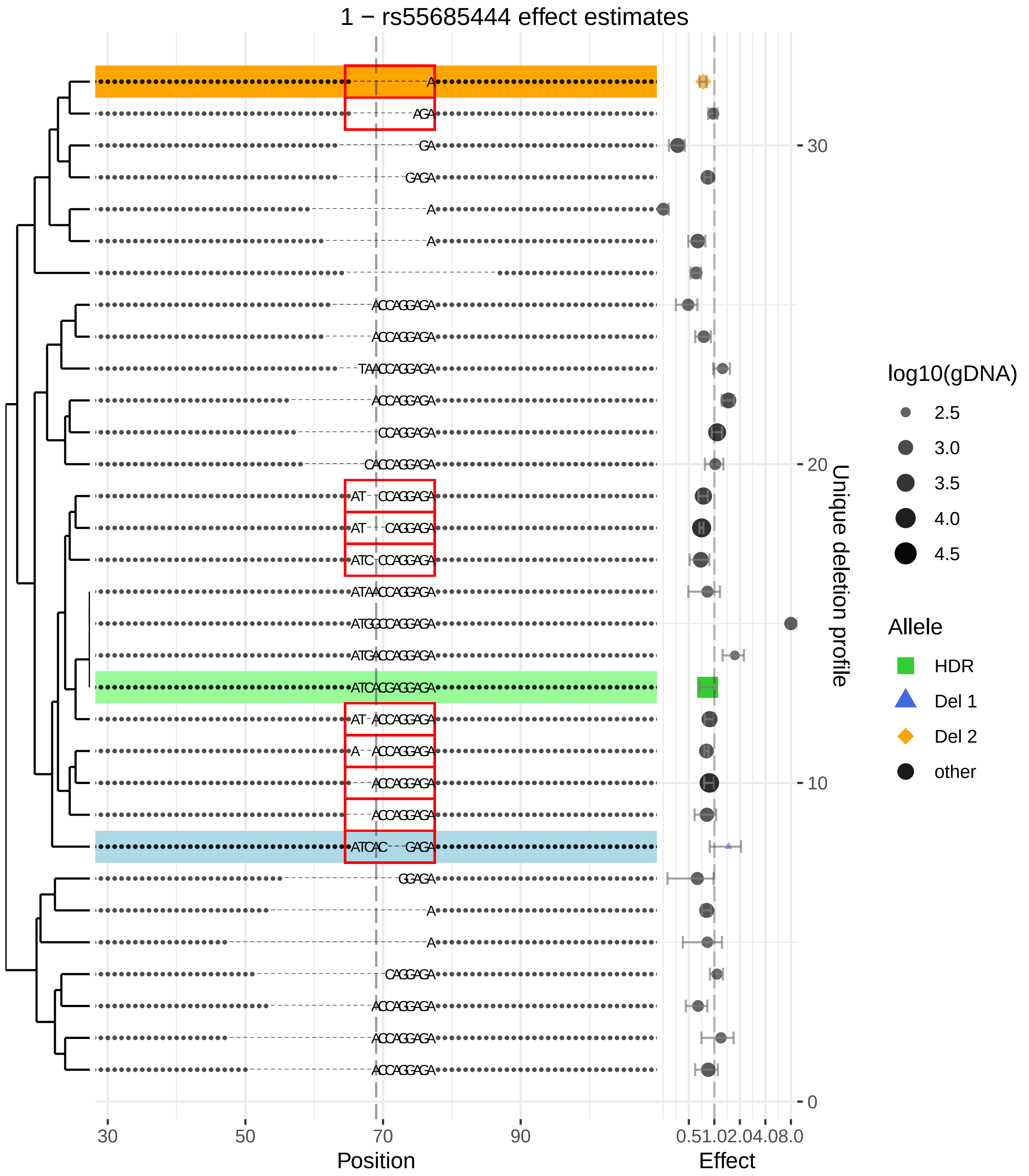

1 − rs55685444 effect estimates
Position
Effect
Unique deletion profile
log10(gDNA)
2.5
3.0
3.5
4.0
4.5
Allele
HDR
Del 1
Del 2
other
30
50
70
90
0.5
1.0
2.0
4.0
8.0
0
10
20
30

2 − rs12269414 grep summary

Mean HDR frac gDNA: 3.9%, ATAC: 1%
Mean WT frac gDNA: 12%, ATAC: 4.2%

ATAC:gDNA ratio (HDR/WT): 0.757
95% CI: (0.669, 0.846),   p = 0.00259

replicate	atac1.1	atac1.2	atac1.3	gDNA1.1	gDNA1.2	gDNA1.3
type	ATAC	ATAC	ATAC	gDNA	gDNA	gDNA
num_reads	150042	140257	160530	88261	74223	41843
HDR reads	1599	1425	1509	3354	2974	1662
WT reads	6254	6152	6507	10839	9127	5290
HDR_WT_ratio	0.256	0.232	0.232	0.309	0.326	0.314
HDR_frac	1.07%	1.02%	0.94%	3.80%	4.01%	3.97%
WT_frac	4.17%	4.39%	4.05%	12.28%	12.30%	12.64%

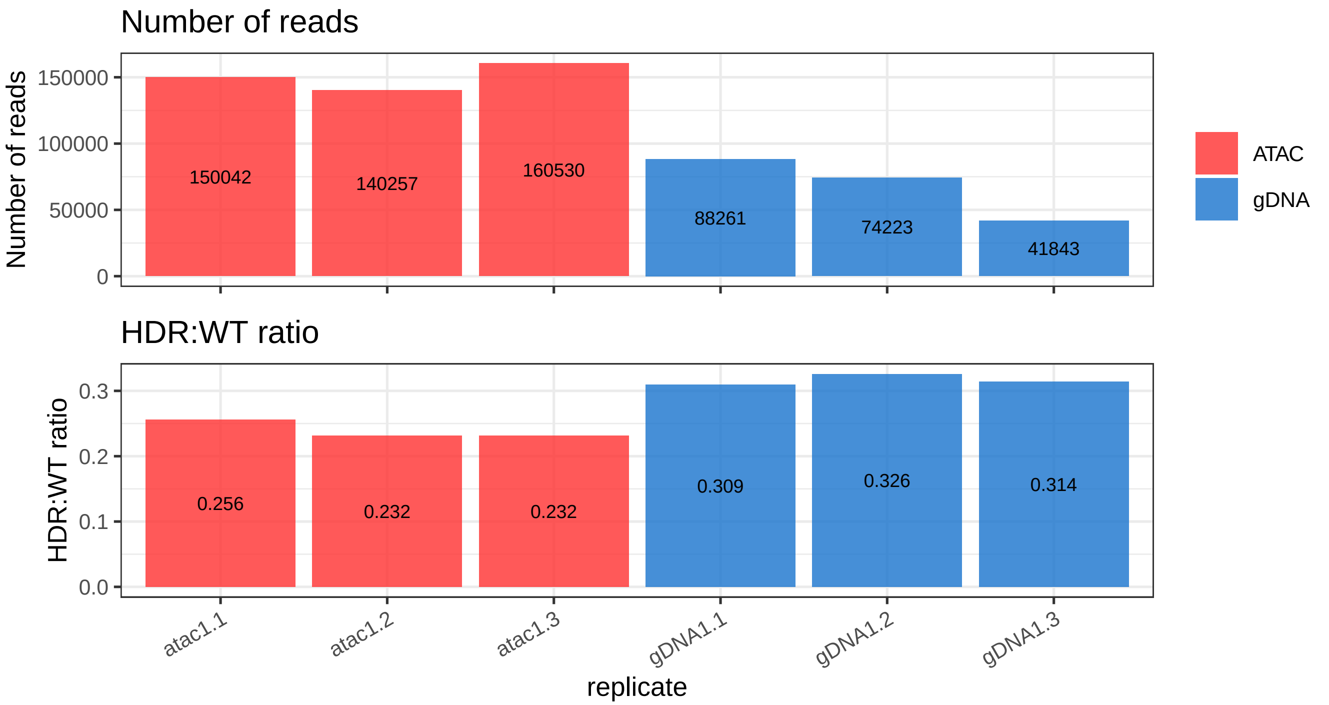

2 − rs12269414 analysis summary

Mean HDR frac gDNA: 4.1%, ATAC: 7%
Mean DEL frac gDNA: 86%, ATAC: 56%
Mean WT frac gDNA: 9.5%, ATAC: 27%

ATAC:gDNA ratio (HDR/WT): 0.596
95% CI: (0.517, 0.676), p = 0.00052

ATAC:gDNA ratio (DEL/WT) [64−76]: 0.227
95% CI: (0.209, 0.245), p = 0.000401
ATAC:gDNA ratio (DEL/WT) − Del 1: 0.202
95% CI: (0.175, 0.23), p = 0.00012
ATAC:gDNA ratio (DEL/WT) − Del 2: 0.239
95% CI: (0.223, 0.256), p = 0.000367

replicate	atac1.1	atac1.2	atac1.3	gDNA1.1	gDNA1.2	gDNA1.3
type	ATAC	ATAC	ATAC	gDNA	gDNA	gDNA
num_udps	395	421	416	2419	2322	1696
HDR_WT_ratio	0.277	0.245	0.247	0.431	0.44	0.42
DEL_WT_ratio	2.08	2.06	2.05	9.32	9.09	8.67
HDR_rate	7.48%	6.72%	6.77%	3.98%	4.15%	4.13%
DEL_rate	56.15%	56.46%	56.00%	86.11%	85.77%	85.31%
editing_rate	63.63%	63.18%	62.77%	90.09%	89.92%	89.45%
WT_rate	27.01%	27.38%	27.38%	9.24%	9.43%	9.84%
num_reads	150042	140257	160530	88261	74223	41843
HDR reads	1519	1329	1417	2983	2603	1453
WT reads	5486	5419	5727	6923	5918	3458
Deletion reads	11404	11173	11714	64523	53802	29980
excluded−insertion	3215	3181	3322	13029	11262	6597
excluded−minoverlap	2	1	3	0	0	0
excluded−mismatches	2	3	2	1	1	0
excluded−nonspanning	126512	117283	136284	300	231	105
excluded−mult.deletions	0	0	0	0	0	0

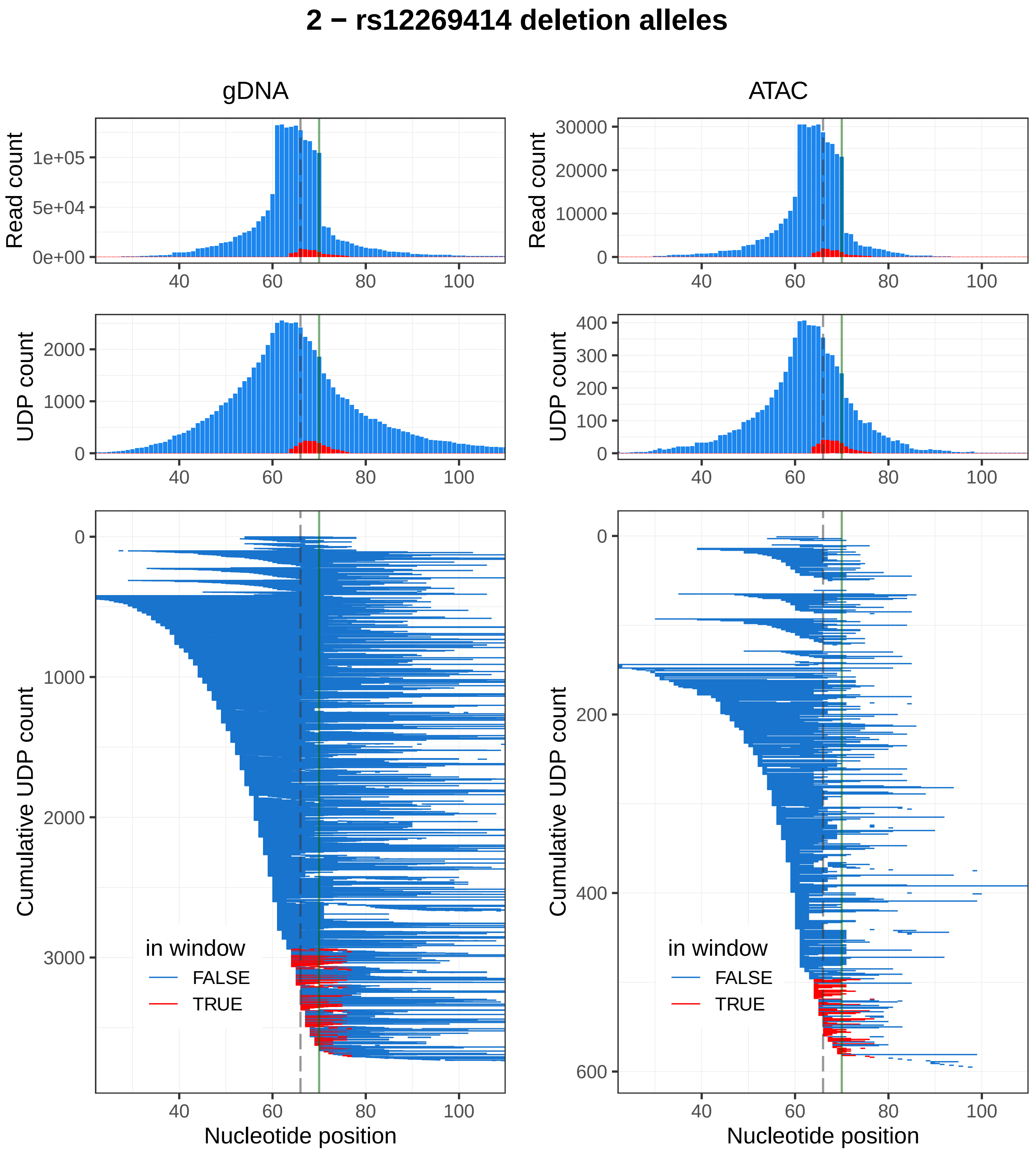
2 − rs12269414 deletion alleles
gDNA
ATAC
Read count
UDP count
Cumulative UDP count
Nucleotide position
in window
FALSE
TRUE
1e+05
5e+04
0e+00
30000
20000
10000
0
2000
1000
400
300
200
100
3000
600
40
60
80
100

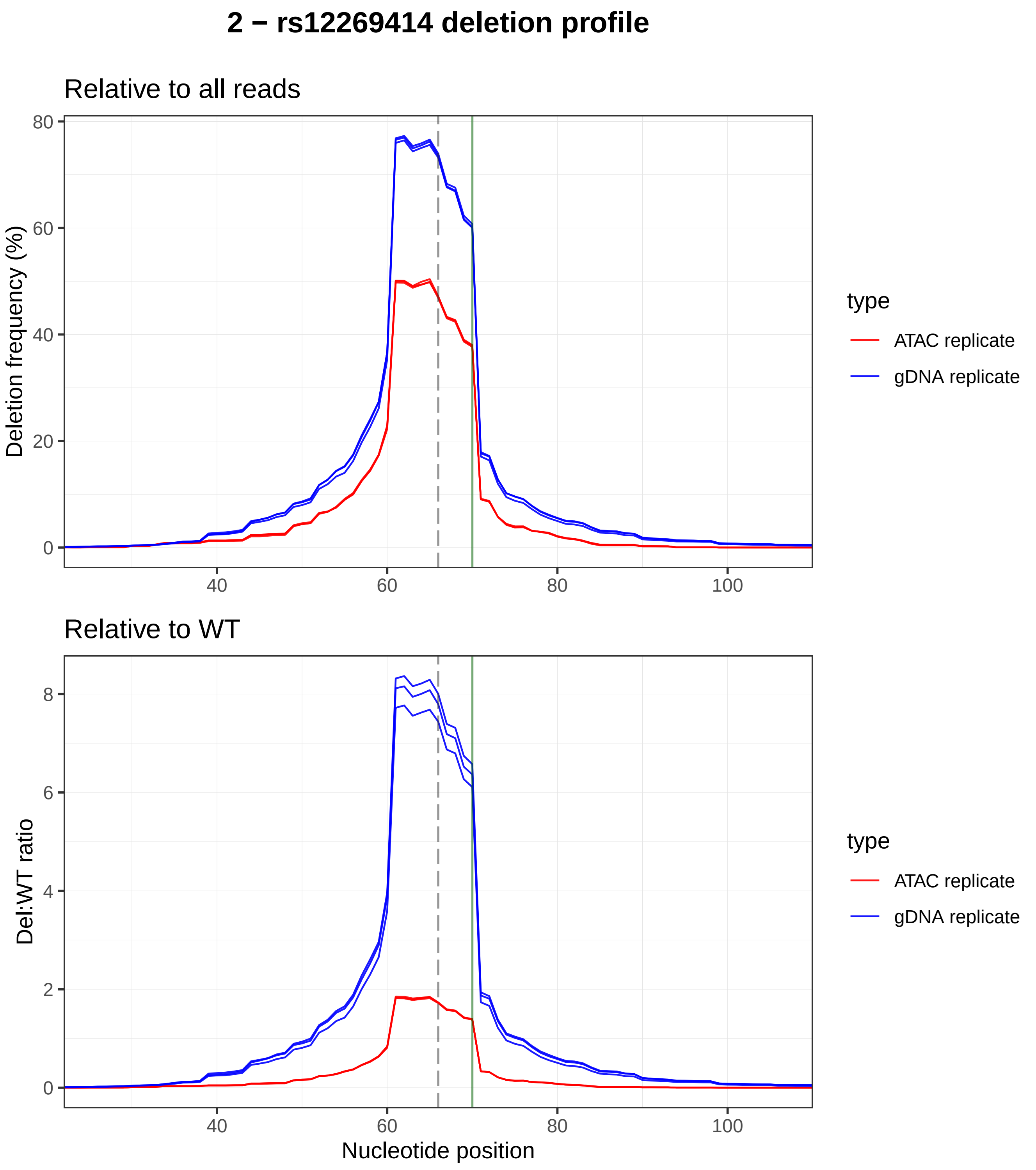
2 − rs12269414 deletion profile
Relative to all reads
Deletion frequency (%)
0
20
40
60
80
40
60
80
100
Relative to WT
Del:WT ratio
0
2
4
6
8
40
60
80
100
Nucleotide position
type
ATAC replicate
gDNA replicate
type
ATAC replicate
gDNA replicate

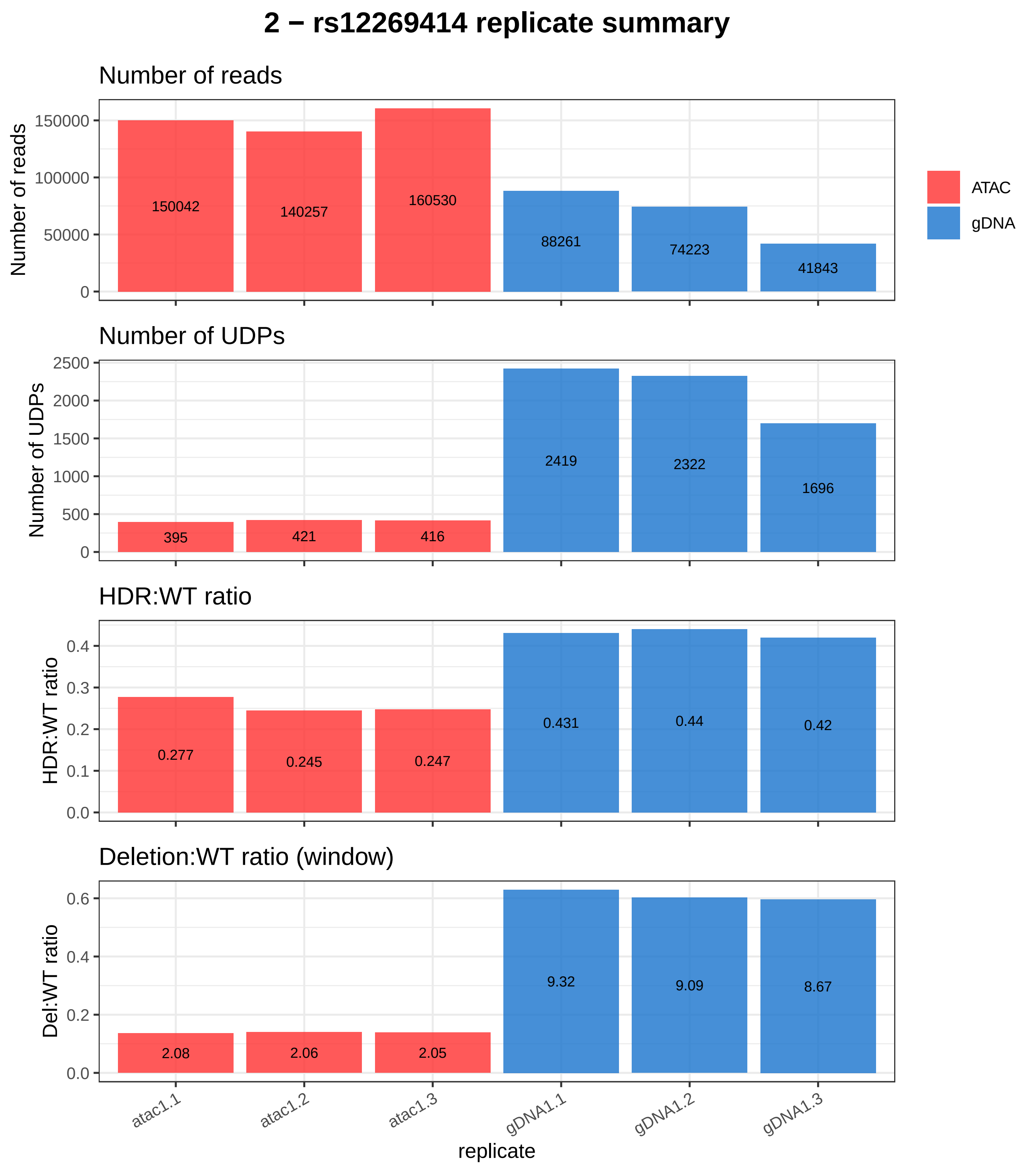
2 − rs12269414 replicate summary
Number of reads
Number of reads
150000
100000
50000
0
150042
140257
160530
88261
74223
41843
ATAC
gDNA
Number of UDPs
Number of UDPs
2500
2000
1500
1000
500
0
395
421
416
2419
2322
1696
HDR:WT ratio
HDR:WT ratio
0.4
0.3
0.2
0.1
0.0
0.277
0.245
0.247
0.431
0.44
0.42
Deletion:WT ratio (window)
Del:WT ratio
0.6
0.4
0.2
0.0
2.08
2.06
2.05
9.32
9.09
8.67
atac1.1
atac1.2
atac1.3
gDNA1.1
gDNA1.2
gDNA1.3
replicate

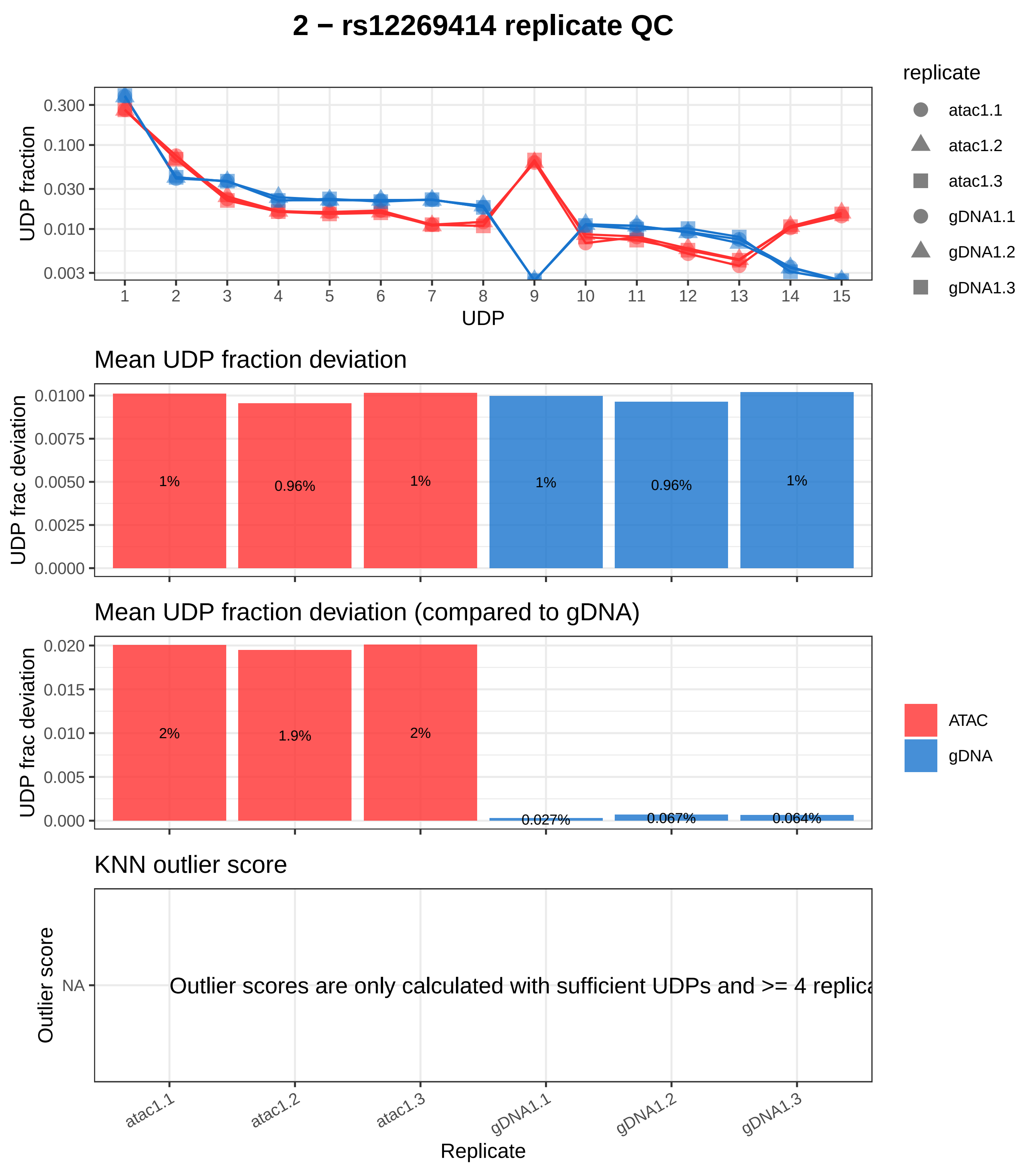

2 – rs12269414 replicate QC
UDP fraction
0.300
0.100
0.030
0.010
0.003
1 2 3 4 5 6 7 8 9 10 11 12 13 14 15
UDP
replicate
atac1.1
atac1.2
atac1.3
gDNA1.1
gDNA1.2
gDNA1.3
Mean UDP fraction deviation
UDP frac deviation
0.0100
0.0075
0.0050
0.0025
0.0000
1%
0.96%
1%
1%
0.96%
1%
Mean UDP fraction deviation (compared to gDNA)
UDP frac deviation
0.020
0.015
0.010
0.005
0.000
2%
1.9%
2%
0.027%
0.067%
0.064%
ATAC
gDNA
KNN outlier score
Outlier score
NA
Outlier scores are only calculated with sufficient UDPs and >= 4 replica
atac1.1
atac1.2
atac1.3
gDNA1.1
gDNA1.2
gDNA1.3
Replicate

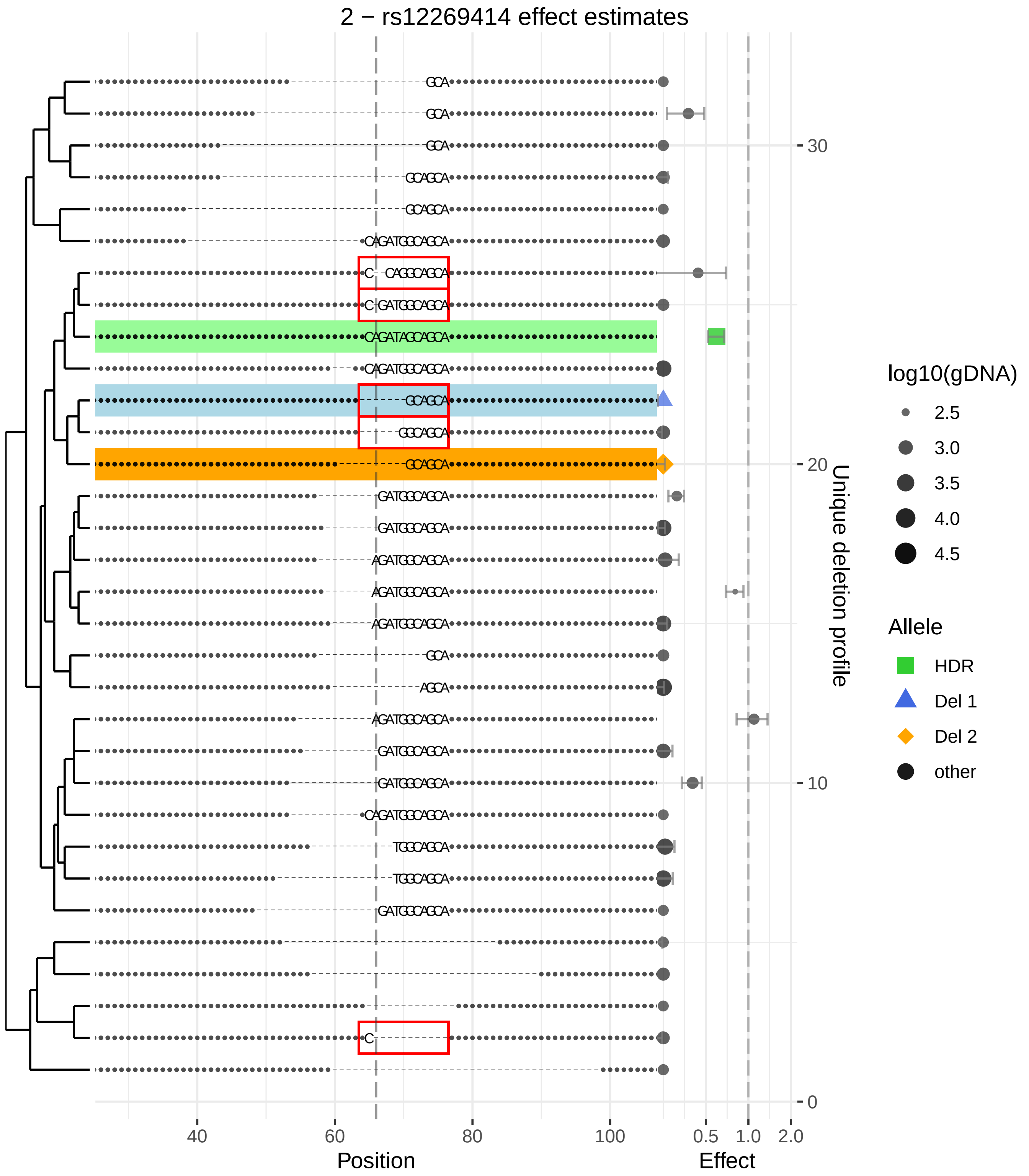
2 − rs12269414 effect estimates
GCA
GCA
GCA
GCAGCA
GCAGCA
CAGATGGCAGCA
C CAGGCAGCA
C GATGGCAGCA
CAGATAGCAGCA
CAGATGGCAGCA
GCAGCA
GGCAGCA
GCAGCA
GATGGCAGCA
GATGGCAGCA
AGATGGCAGCA
AGATGGCAGCA
AGATGGCAGCA
GCA
AGCA
AGATGGCAGCA
GATGGCAGCA
GATGGCAGCA
CAGATGGCAGCA
TGGCAGCA
TGGCAGCA
GATGGCAGCA
C
30
20
10
0
Unique deletion profile
40
60
80
100
Position
0.5
1.0
2.0
Effect
log10(gDNA)
2.5
3.0
3.5
4.0
4.5
Allele
HDR
Del 1
Del 2
other

3 − rs7729529 grep summary

Mean HDR frac gDNA: 44%, ATAC: 6.3%
Mean WT frac gDNA: 11%, ATAC: 1.9%

ATAC:gDNA ratio (HDR/WT): 0.797
95% CI: (0.726, 0.869),   p = 0.00536

replicate	atac1.1	atac1.2	atac1.3	gDNA1.1	gDNA1.2	gDNA1.3
type	ATAC	ATAC	ATAC	gDNA	gDNA	gDNA
num_reads	132843	150852	161773	53996	60299	43507
HDR reads	8572	9440	9771	23650	26677	18896
WT reads	2623	2849	3026	5615	6439	4787
HDR_WT_ratio	3.27	3.31	3.23	4.21	4.14	3.95
HDR_frac	6.45%	6.26%	6.04%	43.80%	44.24%	43.43%
WT_frac	1.97%	1.89%	1.87%	10.40%	10.68%	11.00%

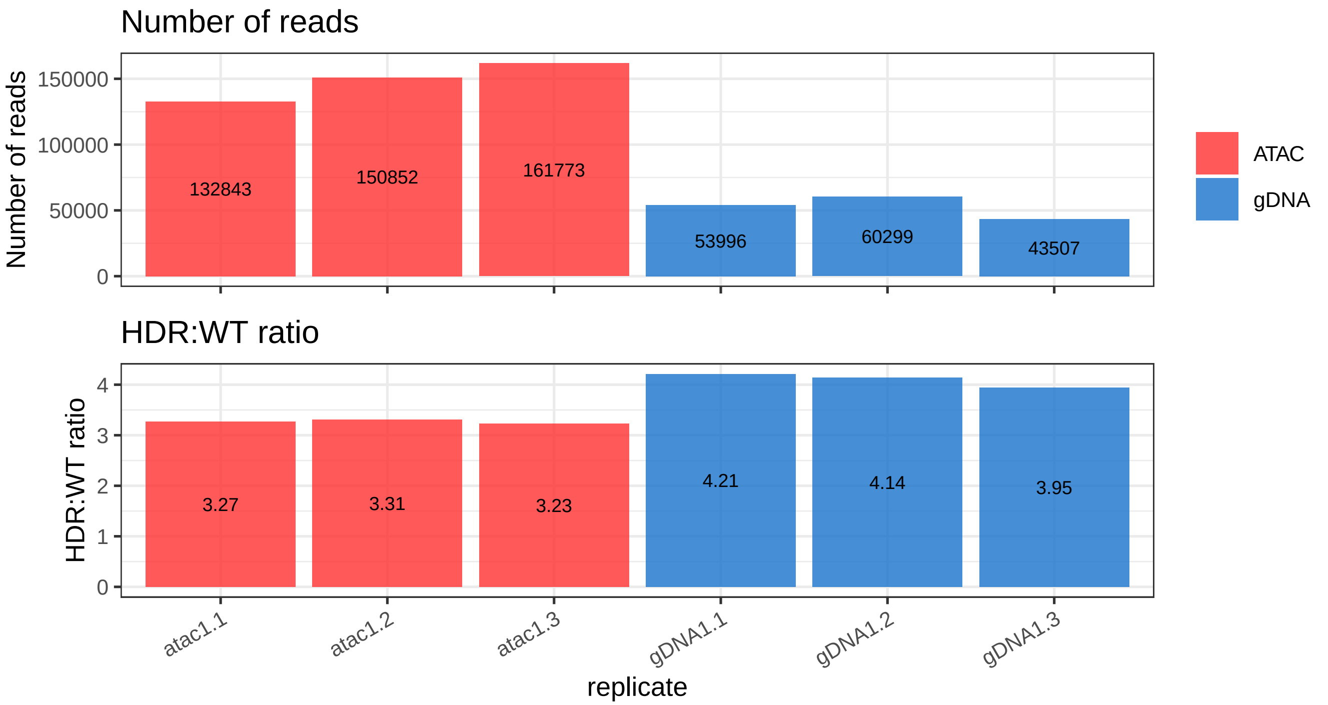

3 − rs7729529 analysis summary

Mean HDR frac gDNA: 45%, ATAC: 42%
Mean DEL frac gDNA: 43%, ATAC: 18%
Mean WT frac gDNA: 11%, ATAC: 13%

ATAC:gDNA ratio (HDR/WT): 0.763
95% CI: (0.693, 0.832), p = 0.00399

ATAC:gDNA ratio (DEL/WT) [118−130]: 0.394
95% CI: (0.373, 0.414), p = 3.24e−05
ATAC:gDNA ratio (DEL/WT) − Del 1: 0.507
95% CI: (0.41, 0.604), p = 0.00387
ATAC:gDNA ratio (DEL/WT) − Del 2: 0.357
95% CI: (0.324, 0.391), p = 4.48e−05

replicate	atac1.1	atac1.2	atac1.3	gDNA1.1	gDNA1.2	gDNA1.3
type	ATAC	ATAC	ATAC	gDNA	gDNA	gDNA
num_udps	435	448	478	1330	1447	1240
HDR_WT_ratio	3.16	3.22	3.14	4.28	4.21	4
DEL_WT_ratio	1.36	1.38	1.39	4.08	4	3.93
HDR_rate	42.30%	42.28%	41.71%	45.00%	45.14%	44.20%
DEL_rate	18.26%	18.15%	18.44%	42.96%	42.84%	43.43%
editing_rate	60.56%	60.43%	60.16%	87.96%	87.98%	87.64%
WT_rate	13.39%	13.11%	13.27%	10.52%	10.71%	11.04%
num_reads	132843	150852	161773	53996	60299	43507
HDR reads	8115	8941	9247	22541	25405	17933
WT reads	2568	2773	2942	5272	6029	4479
Deletion reads	3504	3838	4089	21519	24114	17620
excluded−insertion	460	548	569	2521	2982	2155
excluded−minoverlap	17	21	29	0	0	0
excluded−mismatches	5	2	5	13	5	4
excluded−nonspanning	113176	129135	139001	1370	1030	780
excluded−mult.deletions	0	0	0	0	0	0

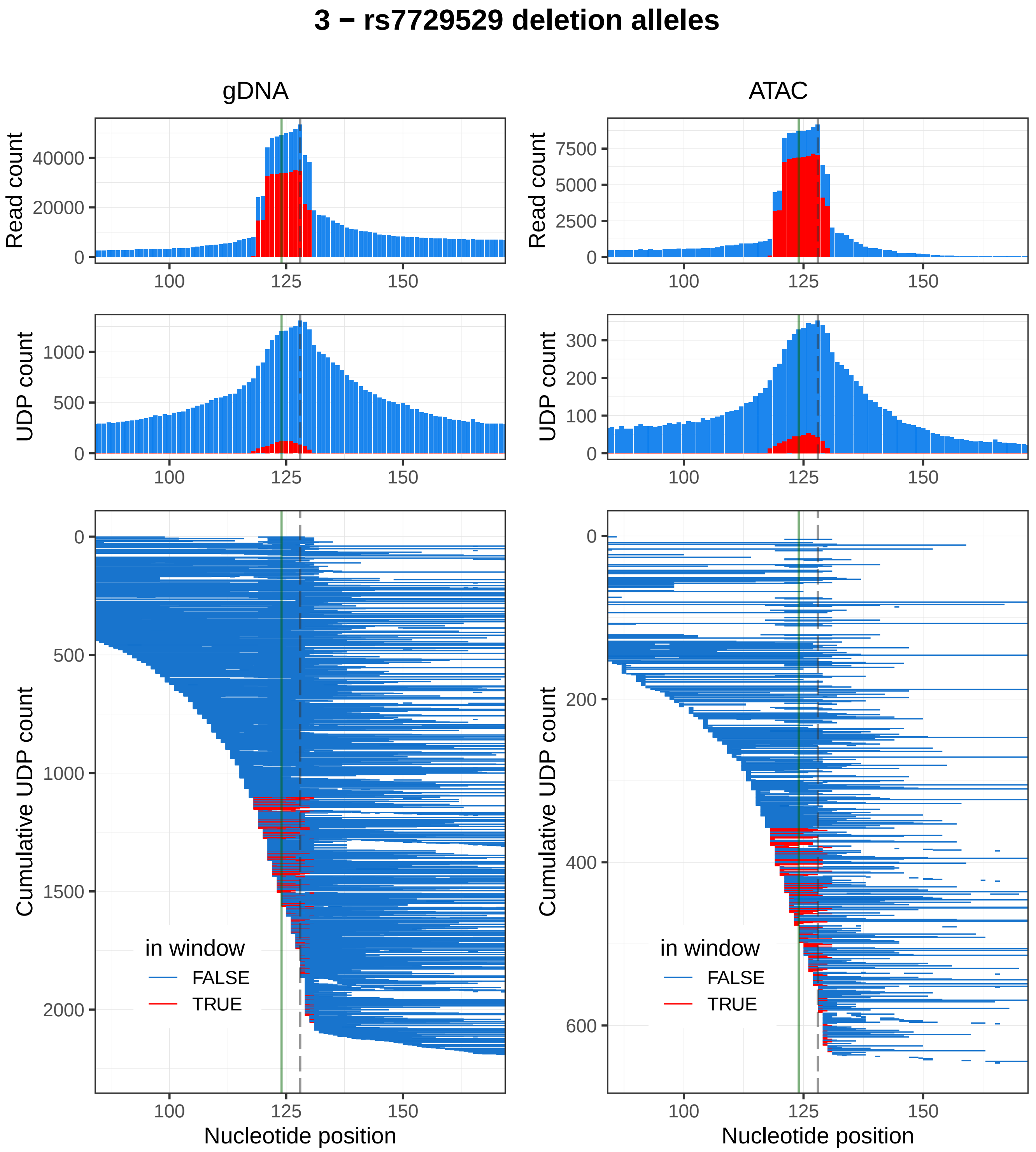
3 − rs7729529 deletion alleles
gDNA
ATAC
Read count
UDP count
Cumulative UDP count
Nucleotide position
in window
FALSE
TRUE
100
125
150
0
20000
40000
2500
5000
7500
500
1000
100
200
300
1500
2000
400
600

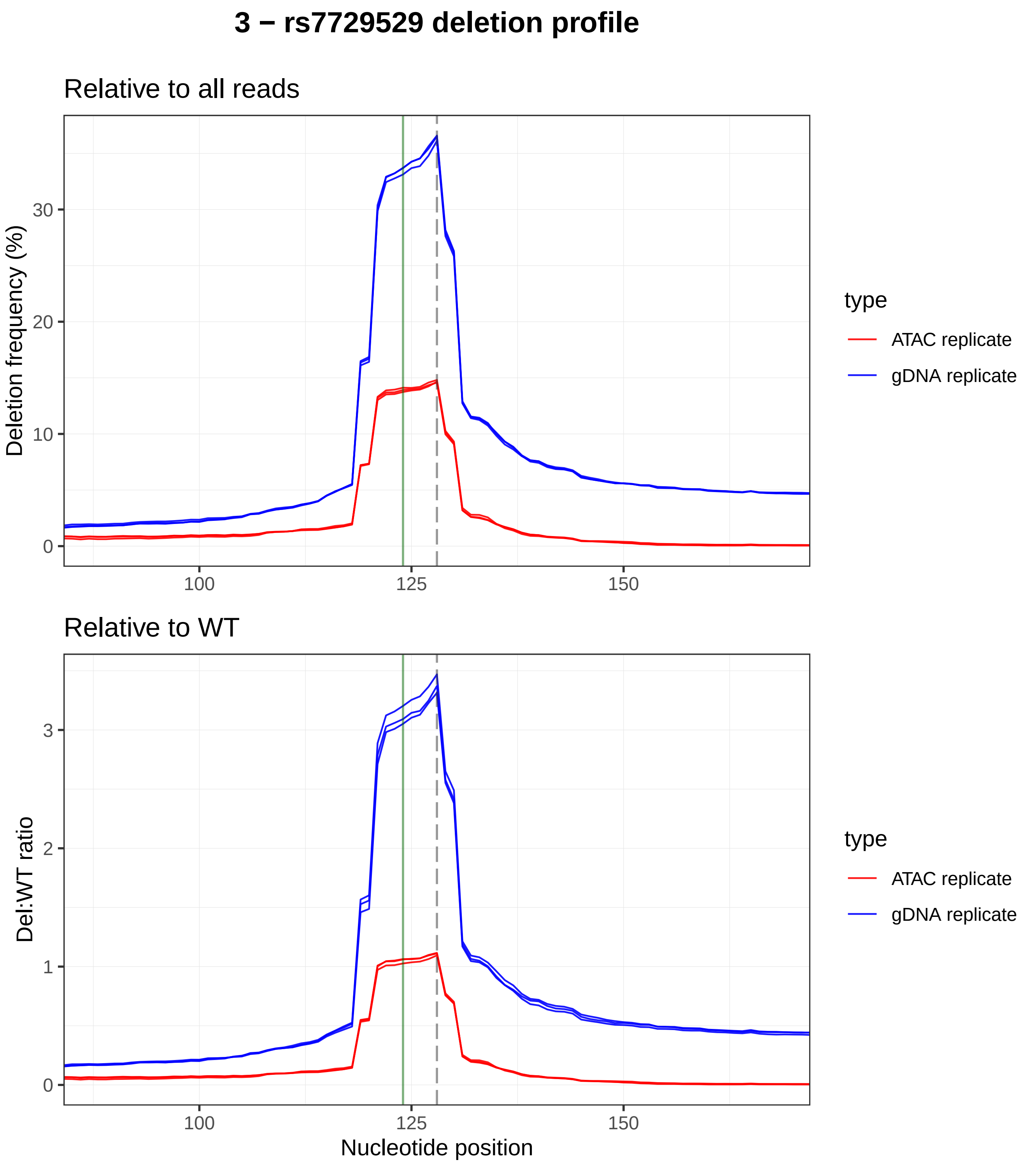

3 − rs7729529 deletion profile
Relative to all reads
Deletion frequency (%)
0
10
20
30
100
125
150
type
ATAC replicate
gDNA replicate
Relative to WT
Del:WT ratio
0
1
2
3
100
125
150
Nucleotide position
type
ATAC replicate
gDNA replicate

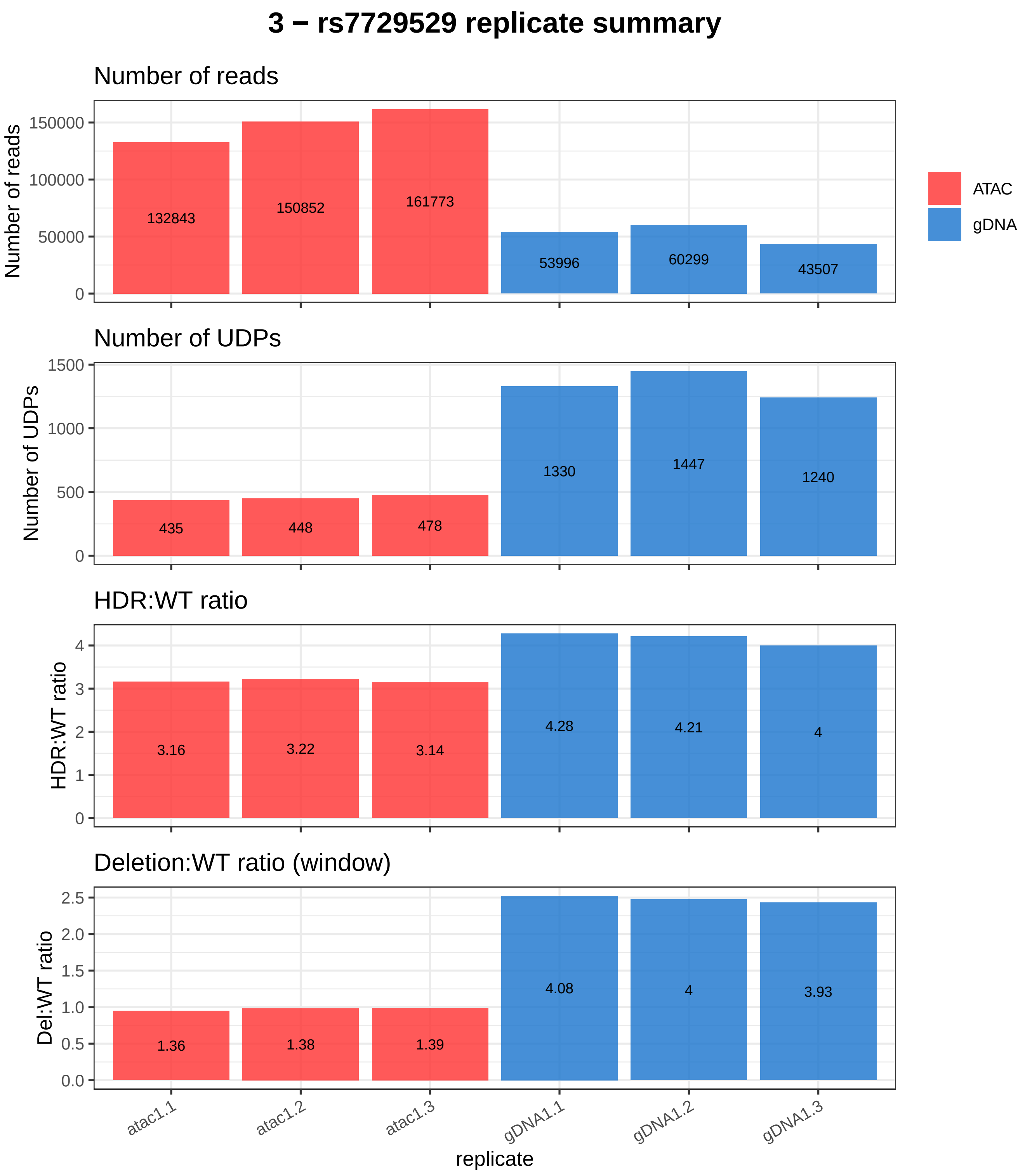
3 − rs7729529 replicate summary
Number of reads
Number of reads
150000
100000
50000
0
132843
150852
161773
53996
60299
43507
ATAC
gDNA
Number of UDPs
Number of UDPs
1500
1000
500
0
435
448
478
1330
1447
1240
HDR:WT ratio
HDR:WT ratio
4
3
2
1
0
3.16
3.22
3.14
4.28
4.21
4
Deletion:WT ratio (window)
Del:WT ratio
2.5
2.0
1.5
1.0
0.5
0.0
1.36
1.38
1.39
4.08
4
3.93
atac1.1
atac1.2
atac1.3
gDNA1.1
gDNA1.2
gDNA1.3
replicate

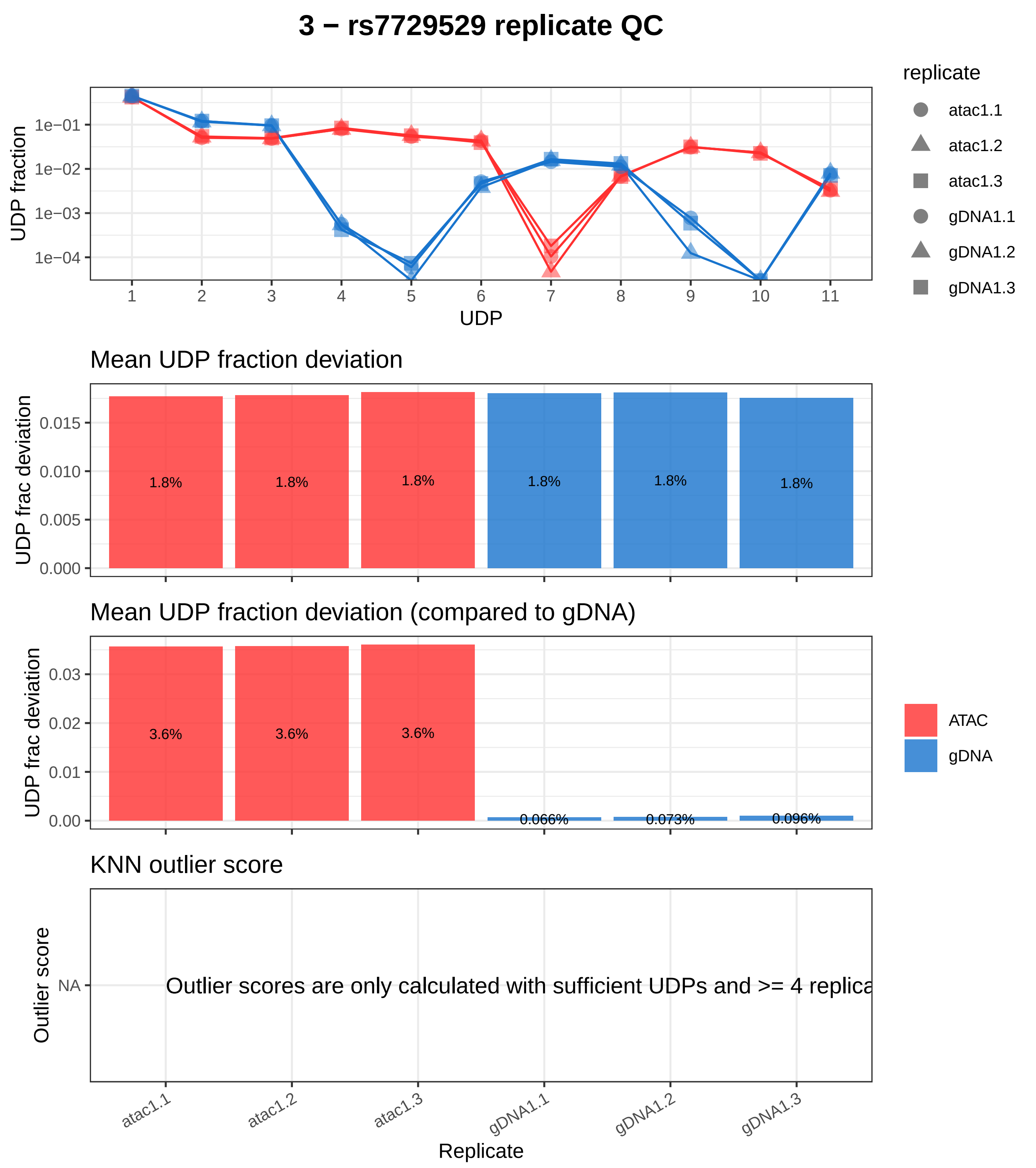
3 − rs7729529 replicate QC
UDP fraction
1e−01
1e−02
1e−03
1e−04
1 2 3 4 5 6 7 8 9 10 11
UDP
replicate
atac1.1
atac1.2
atac1.3
gDNA1.1
gDNA1.2
gDNA1.3
Mean UDP fraction deviation
UDP frac deviation
0.015
0.010
0.005
0.000
1.8%
1.8%
1.8%
1.8%
1.8%
1.8%
Mean UDP fraction deviation (compared to gDNA)
UDP frac deviation
0.03
0.02
0.01
0.00
3.6%
3.6%
3.6%
0.066%
0.073%
0.096%
ATAC
gDNA
KNN outlier score
Outlier score
NA
Outlier scores are only calculated with sufficient UDPs and >= 4 replica
atac1.1
atac1.2
atac1.3
gDNA1.1
gDNA1.2
gDNA1.3
Replicate

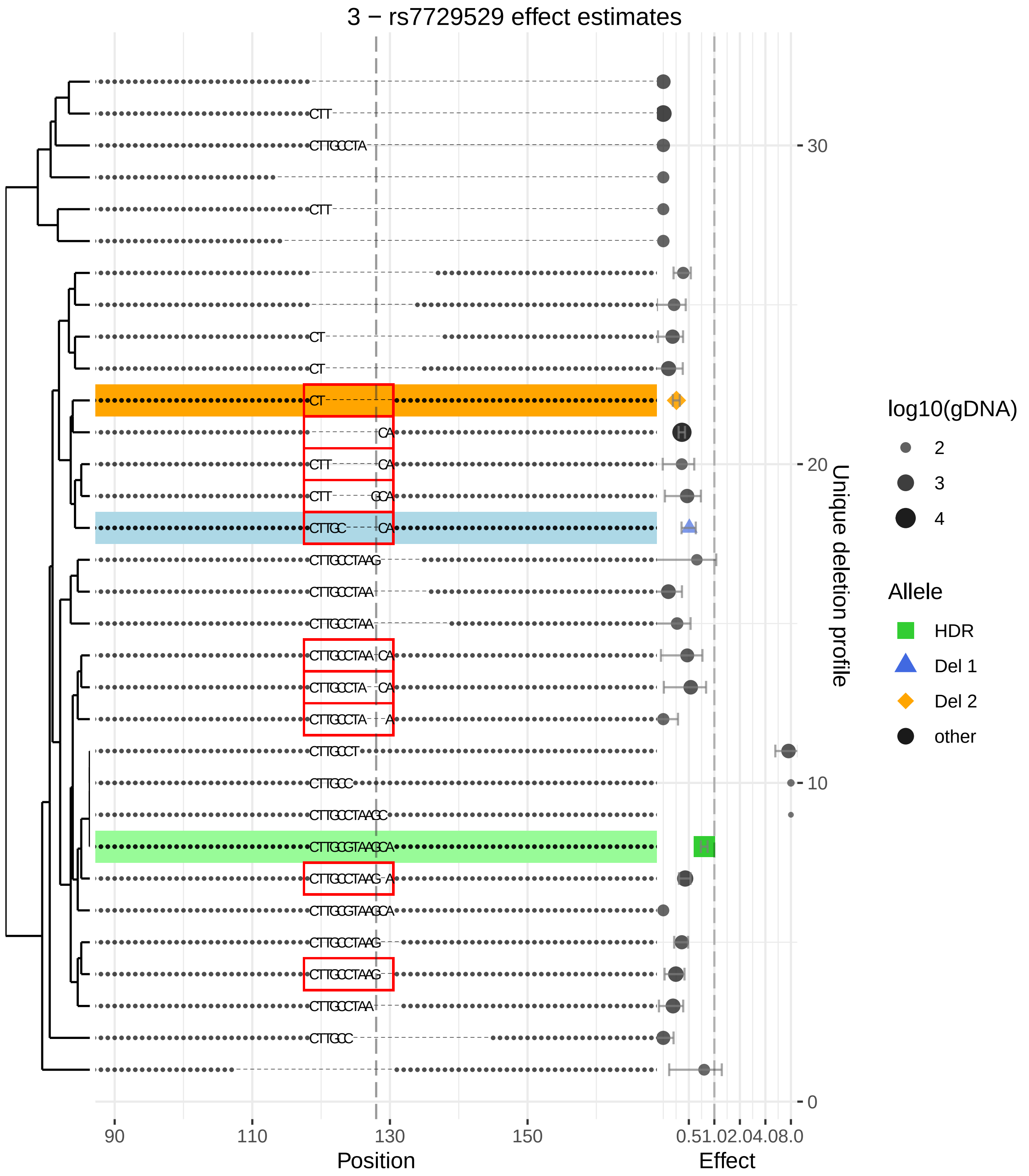

3 − rs7729529 effect estimates
CTT
CTTGCCTA
CTT
CT
CT
CT
CA
CTT
CA
CTT
GCA
CTTGC
CA
CTTGCCTAAG
CTTGCCTAA
CTTGCCTAA
CTTGCCTAA
CA
CTTGCCTA
CA
CTTGCCTA
A
CTTGCCT
CTTGCC
CTTGCCTAAGC
CTTGCGTAAGCA
CTTGCCTAAG
A
CTTGCGTAAGCA
CTTGCCTAAG
CTTGCCTAAG
CTTGCCTAA
CTTGCC
H
0
10
20
30
Unique deletion profile
90
110
130
150
Position
0.5
1.0
2.0
4.0
8.0
Effect
log10(gDNA)
2
3
4
Allele
HDR
Del 1
Del 2
other

4 − rs6700034 grep summary

Mean HDR frac gDNA: 4.4%, ATAC: 0.79%
Mean WT frac gDNA: 20%, ATAC: 3.4%

ATAC:gDNA ratio (HDR/WT): 1.03
95% CI: (0.938, 1.11),   p = 0.418

replicate	atac1.1	atac1.2	atac1.3	gDNA1.1	gDNA1.2	gDNA1.3
type	ATAC	ATAC	ATAC	gDNA	gDNA	gDNA
num_reads	149585	143817	129001	55728	78731	44584
HDR reads	1254	1112	966	2306	3763	1877
WT reads	5230	5024	4254	10382	16428	8539
HDR_WT_ratio	0.24	0.221	0.227	0.222	0.229	0.22
HDR_frac	0.84%	0.77%	0.75%	4.14%	4.78%	4.21%
WT_frac	3.50%	3.49%	3.30%	18.63%	20.87%	19.15%

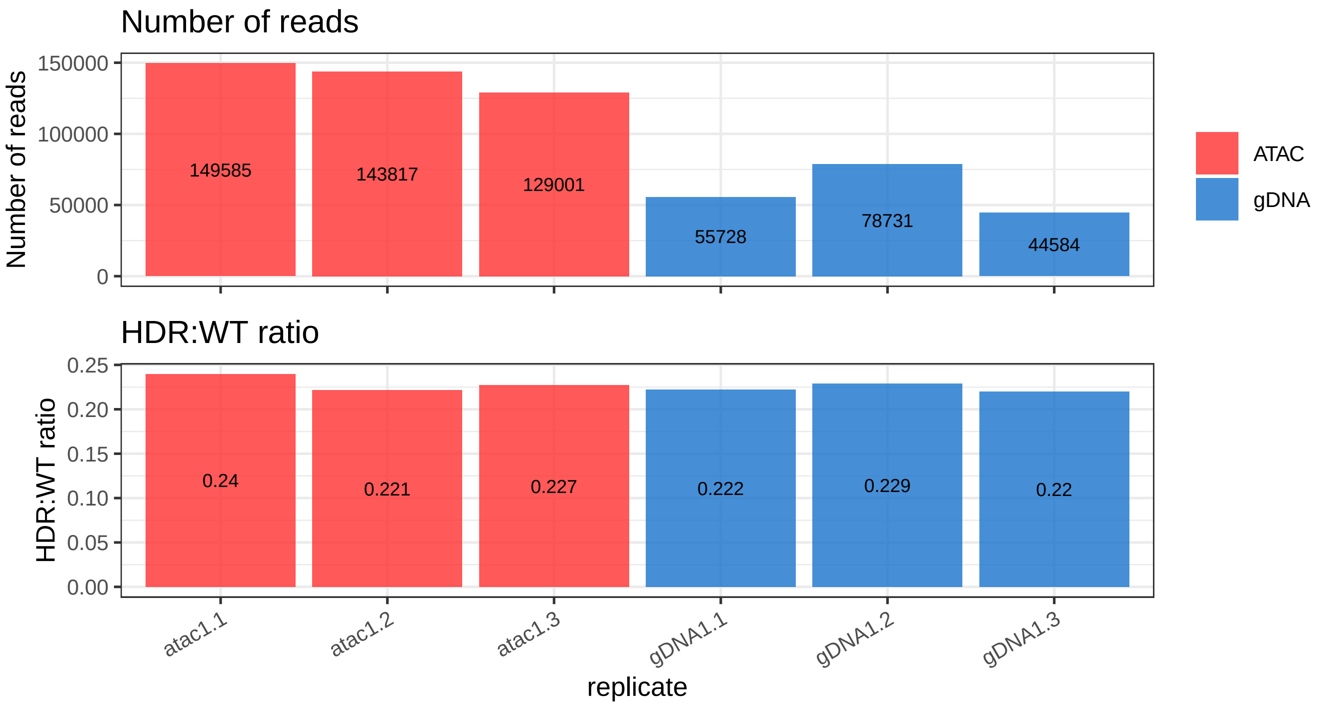

4 − rs6700034 analysis summary

Mean HDR frac gDNA: 4.8%, ATAC: 6.9%
Mean DEL frac gDNA: 71%, ATAC: 52%
Mean WT frac gDNA: 23%, ATAC: 31%

ATAC:gDNA ratio (HDR/WT): 1.08
95% CI: (0.987, 1.17), p = 0.0774

ATAC:gDNA ratio (DEL/WT) [129−141]: 0.735
95% CI: (0.69, 0.779), p = 0.000224
ATAC:gDNA ratio (DEL/WT) − Del 1: 0.914
95% CI: (0.684, 1.14), p = 0.383
ATAC:gDNA ratio (DEL/WT) − Del 2: 0.5
95% CI: (0.455, 0.545), p = 0.000423

replicate	atac1.1	atac1.2	atac1.3	gDNA1.1	gDNA1.2	gDNA1.3
type	ATAC	ATAC	ATAC	gDNA	gDNA	gDNA
num_udps	554	532	506	1465	1829	1348
HDR_WT_ratio	0.233	0.215	0.22	0.205	0.211	0.203
DEL_WT_ratio	1.73	1.64	1.66	3.05	3.09	2.9
HDR_rate	7.08%	6.77%	6.90%	4.77%	4.86%	4.91%
DEL_rate	52.63%	51.71%	52.13%	71.07%	71.21%	70.10%
editing_rate	59.71%	58.48%	59.03%	75.84%	76.07%	75.00%
WT_rate	30.46%	31.54%	31.40%	23.29%	23.05%	24.15%
num_reads	149585	143817	129001	55728	78731	44584
HDR reads	1209	1071	928	2104	3431	1714
WT reads	5198	4990	4221	10272	16269	8439
Deletion reads	8981	8181	7008	31350	50250	24490
excluded−insertion	660	618	529	3322	5012	2426
excluded−minoverlap	13	17	17	56	110	65
excluded−mismatches	7	8	7	1	1	1
excluded−nonspanning	131839	127352	115005	8236	3040	7154
excluded−mult.deletions	0	0	0	0	0	0

4 − rs6700034 deletion alleles

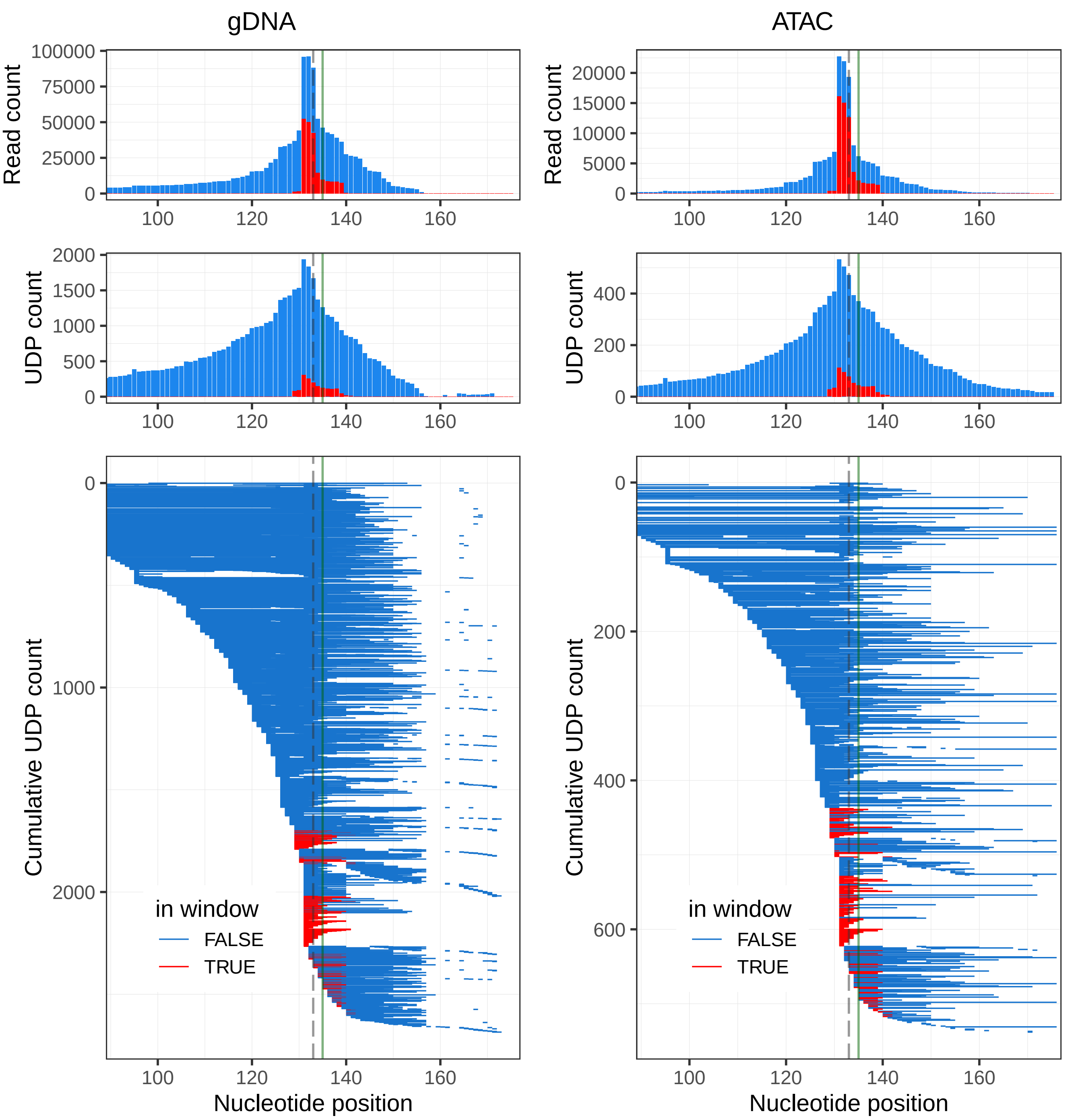

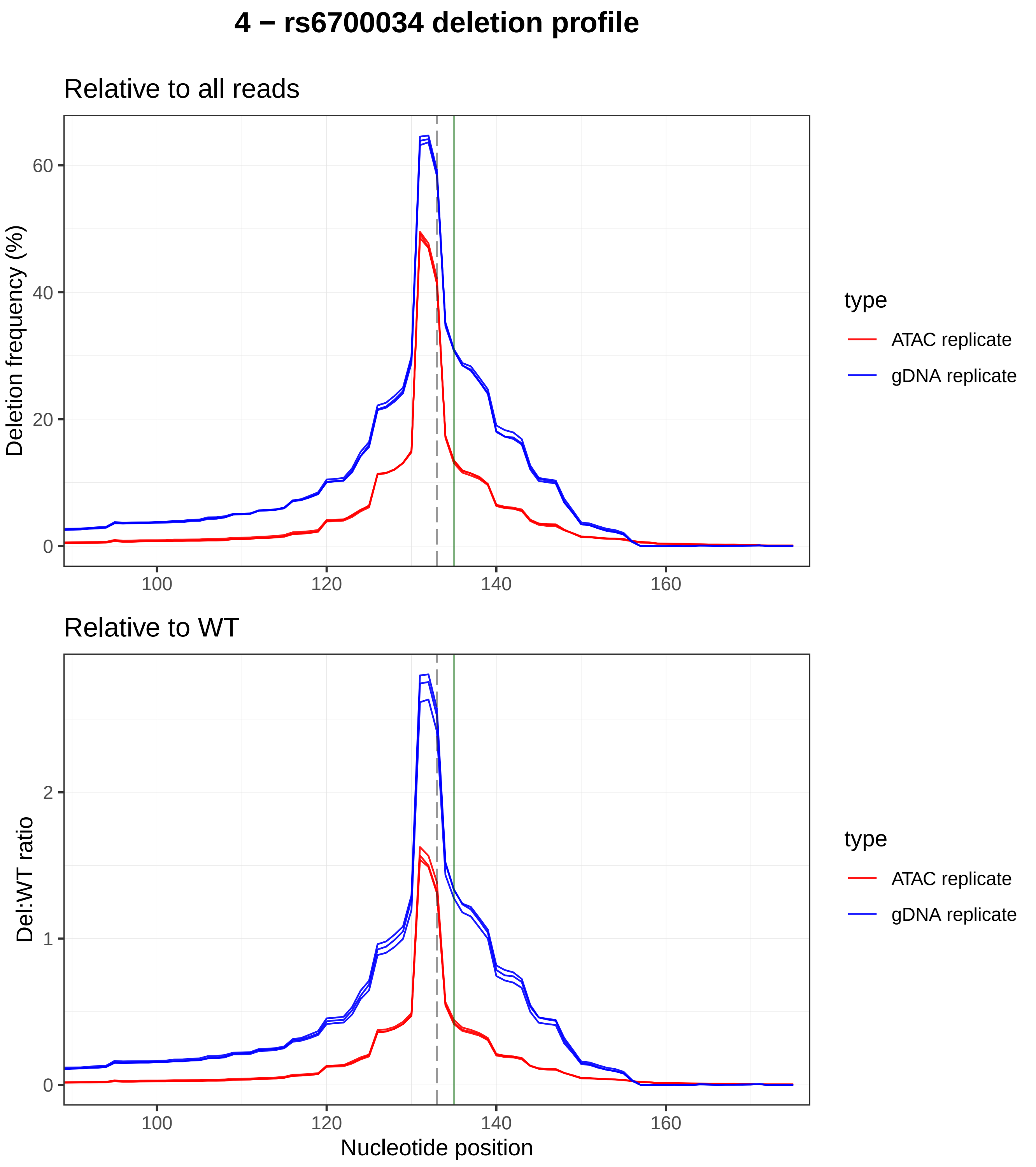

4 − rs6700034 deletion profile
Relative to all reads
Deletion frequency (%)
60
40
20
0
100
120
140
160
type
ATAC replicate
gDNA replicate
Relative to WT
Del:WT ratio
2
1
0
100
120
140
160
Nucleotide position
type
ATAC replicate
gDNA replicate

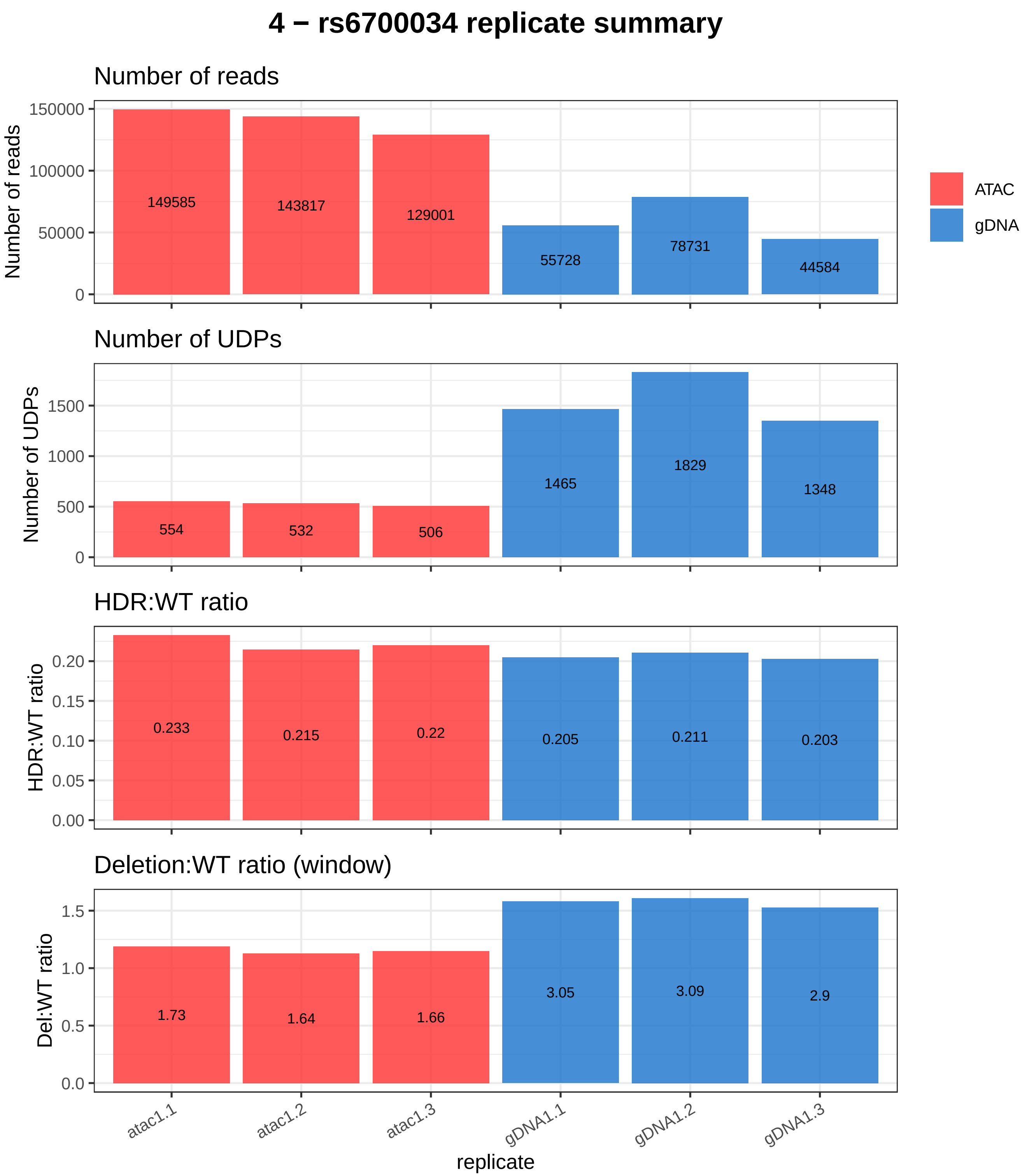
4 − rs6700034 replicate summary
Number of reads
Number of reads
150000
100000
50000
0
149585
143817
129001
55728
78731
44584
ATAC
gDNA
Number of UDPs
Number of UDPs
1500
1000
500
0
554
532
506
1465
1829
1348
HDR:WT ratio
HDR:WT ratio
0.20
0.15
0.10
0.05
0.00
0.233
0.215
0.22
0.205
0.211
0.203
Deletion:WT ratio (window)
Del:WT ratio
1.5
1.0
0.5
0.0
1.73
1.64
1.66
3.05
3.09
2.9
atac1.1
atac1.2
atac1.3
gDNA1.1
gDNA1.2
gDNA1.3
replicate

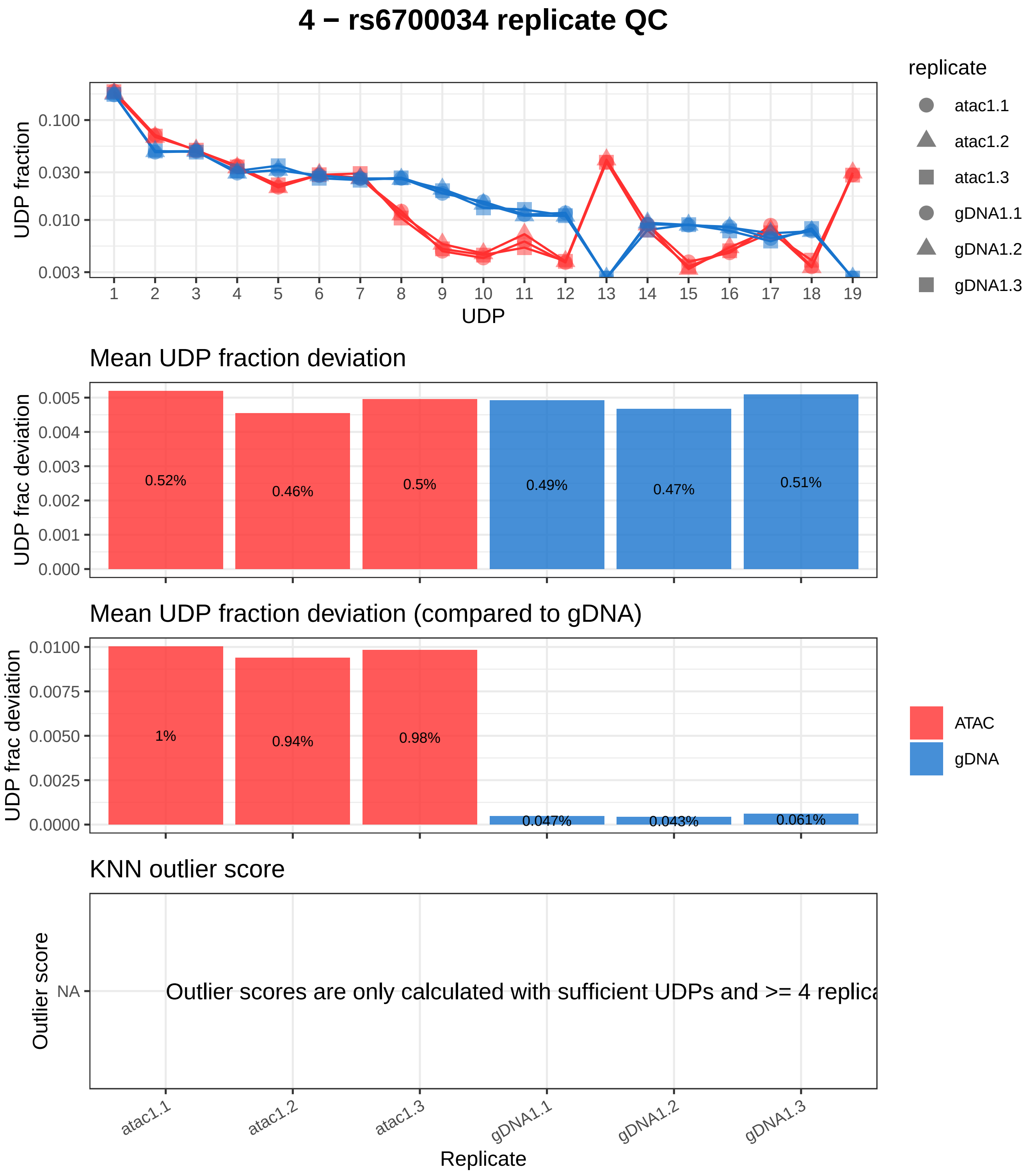

4 − rs6700034 replicate QC
UDP fraction
0.100
0.030
0.010
0.003
1 2 3 4 5 6 7 8 9 10 11 12 13 14 15 16 17 18 19
UDP
replicate
atac1.1
atac1.2
atac1.3
gDNA1.1
gDNA1.2
gDNA1.3
Mean UDP fraction deviation
UDP frac deviation
0.005
0.004
0.003
0.002
0.001
0.000
0.52%
0.46%
0.5%
0.49%
0.47%
0.51%
Mean UDP fraction deviation (compared to gDNA)
UDP frac deviation
0.0100
0.0075
0.0050
0.0025
0.0000
1%
0.94%
0.98%
0.047%
0.043%
0.061%
ATAC
gDNA
KNN outlier score
Outlier score
NA
Outlier scores are only calculated with sufficient UDPs and >= 4 replica
atac1.1
atac1.2
atac1.3
gDNA1.1
gDNA1.2
gDNA1.3
Replicate

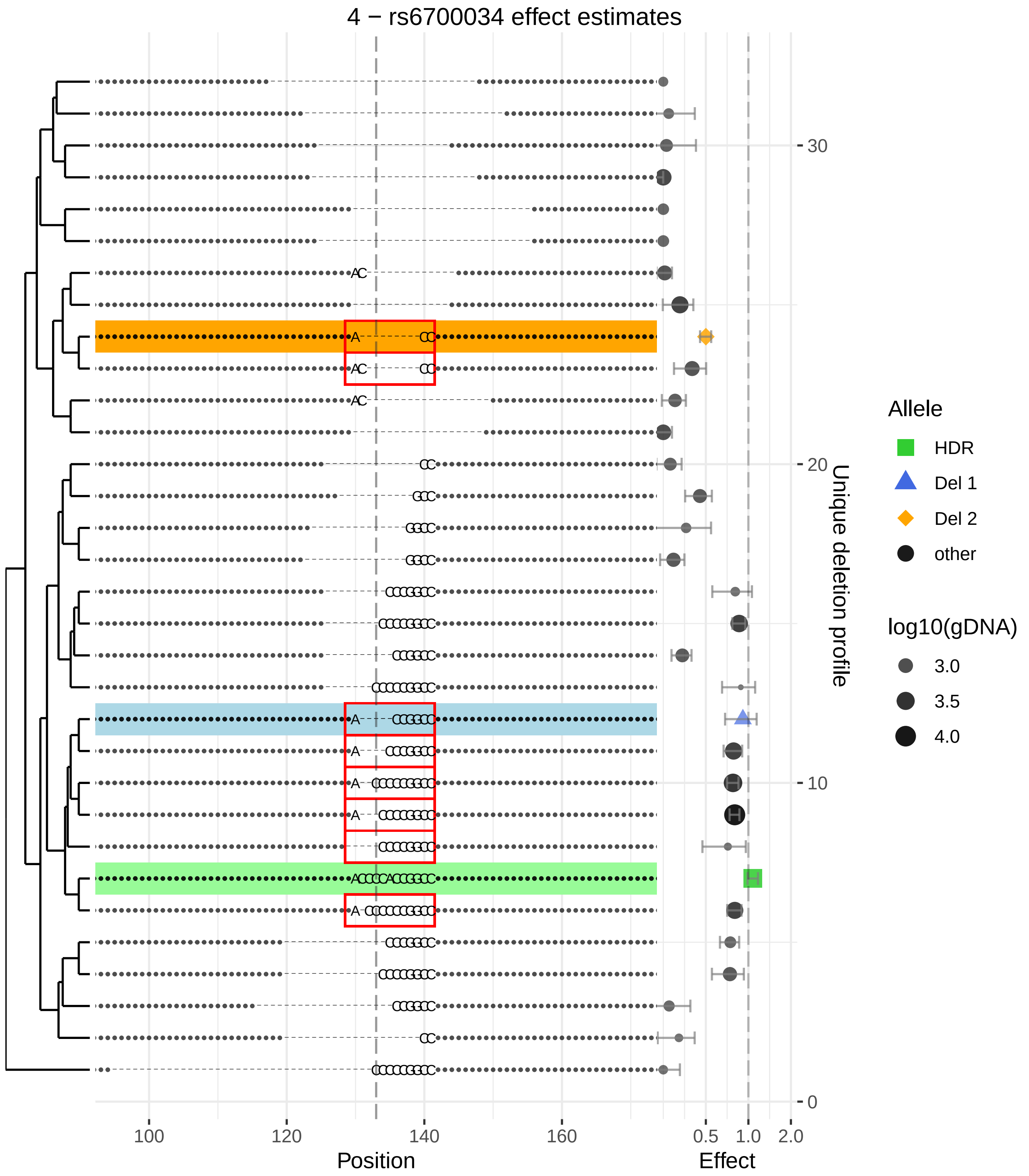
4 − rs6700034 effect estimates
Position
100
120
140
160
Effect
0.5
1.0
2.0
Unique deletion profile
0
10
20
30
Allele
HDR
Del 1
Del 2
other
log10(gDNA)
3.0
3.5
4.0

5 − rs8011143 grep summary

Mean HDR frac gDNA: 37%, ATAC: 2.2%
Mean WT frac gDNA: 1.6%, ATAC: 0.11%

ATAC:gDNA ratio (HDR/WT): 0.87
95% CI: (0.646, 1.09),   p = 0.108

replicate	atac1.1	atac1.2	atac1.3	gDNA1.1	gDNA1.2	gDNA1.3
type	ATAC	ATAC	ATAC	gDNA	gDNA	gDNA
num_reads	205071	179189	208632	64434	63679	49400
HDR reads	4455	4065	4689	23498	23489	18100
WT reads	245	210	213	1008	1016	822
HDR_WT_ratio	18.2	19.4	22	23.3	23.1	22
HDR_frac	2.17%	2.27%	2.25%	36.47%	36.89%	36.64%
WT_frac	0.12%	0.12%	0.10%	1.56%	1.60%	1.66%

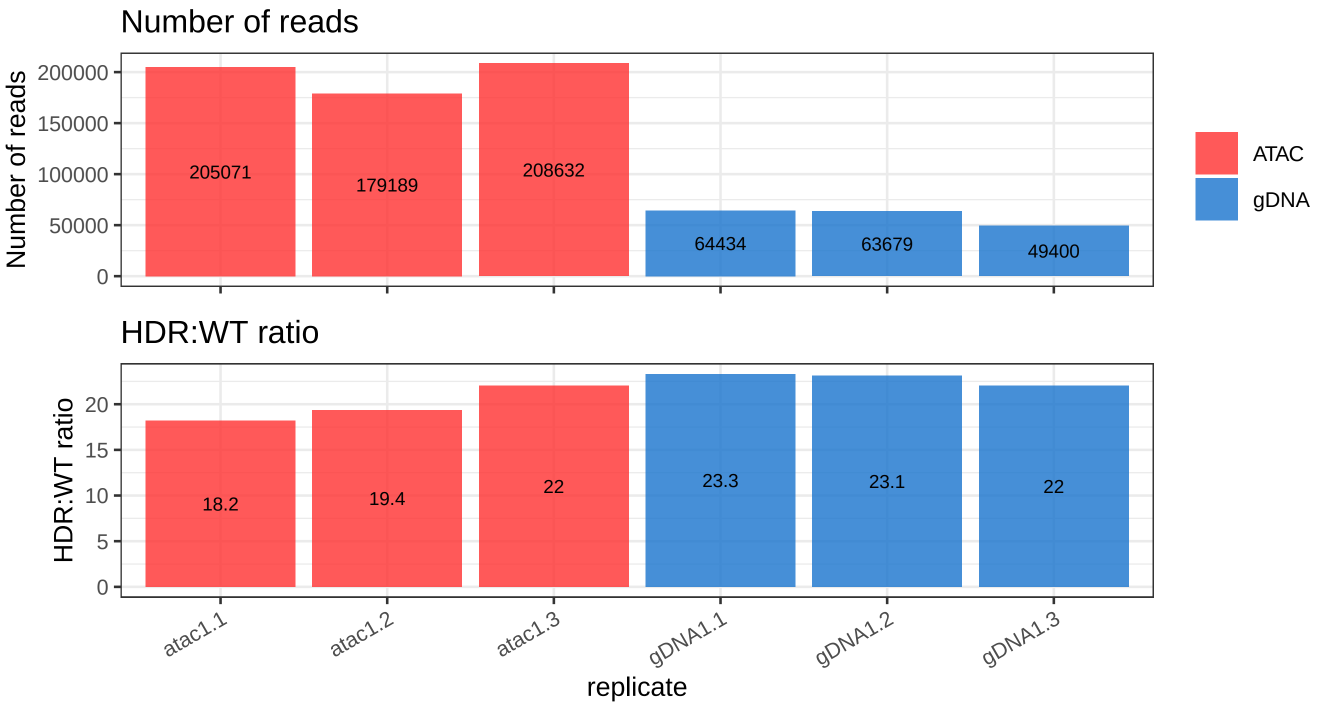

5 − rs8011143 analysis summary

Mean HDR frac gDNA: 45%, ATAC: 40%
Mean DEL frac gDNA: 51%, ATAC: 52%
Mean WT frac gDNA: 1.8%, ATAC: 1.8%

ATAC:gDNA ratio (HDR/WT): 0.905
95% CI: (0.768, 1.04),   p = 0.142

ATAC:gDNA ratio (DEL/WT) [84−96]: 1.3
95% CI: (1.01, 1.59),   p = 0.0484
ATAC:gDNA ratio (DEL/WT) − Del 1: 1.59
95% CI: (0.933, 2.25),   p = 0.0678
ATAC:gDNA ratio (DEL/WT) − Del 2: 2.29
95% CI: (0.377, 4.21),   p = 0.098

replicate	atac1.1	atac1.2	atac1.3	gDNA1.1	gDNA1.2	gDNA1.3
type	ATAC	ATAC	ATAC	gDNA	gDNA	gDNA
num_udps	559	531	565	2155	2081	1873
HDR_WT_ratio	20.7	22.8	24.3	25.7	25.8	23.6
DEL_WT_ratio	26.7	30.6	30.7	29.3	28.6	26.8
HDR_rate	39.87%	39.22%	40.57%	45.16%	45.80%	45.21%
DEL_rate	51.38%	52.62%	51.18%	51.52%	50.82%	51.32%
editing_rate	91.25%	91.85%	91.75%	96.68%	96.62%	96.53%
WT_rate	1.92%	1.72%	1.67%	1.76%	1.78%	1.92%
num_reads	205071	179189	208632	64434	63679	49400
HDR reads	4207	3882	4426	22403	22509	17252
WT reads	203	170	182	871	874	732
Deletion reads	5422	5208	5584	25557	24975	19585
excluded−insertion	3006	2903	3070	14455	14005	10971
excluded−minoverlap	8	4	3	0	0	0
excluded−mismatches	1	0	2	2	1	0
excluded−nonspanning	191504	166385	194647	369	530	269
excluded−mult.deletions	0	0	0	0	0	0

5 − rs8011143 deletion alleles

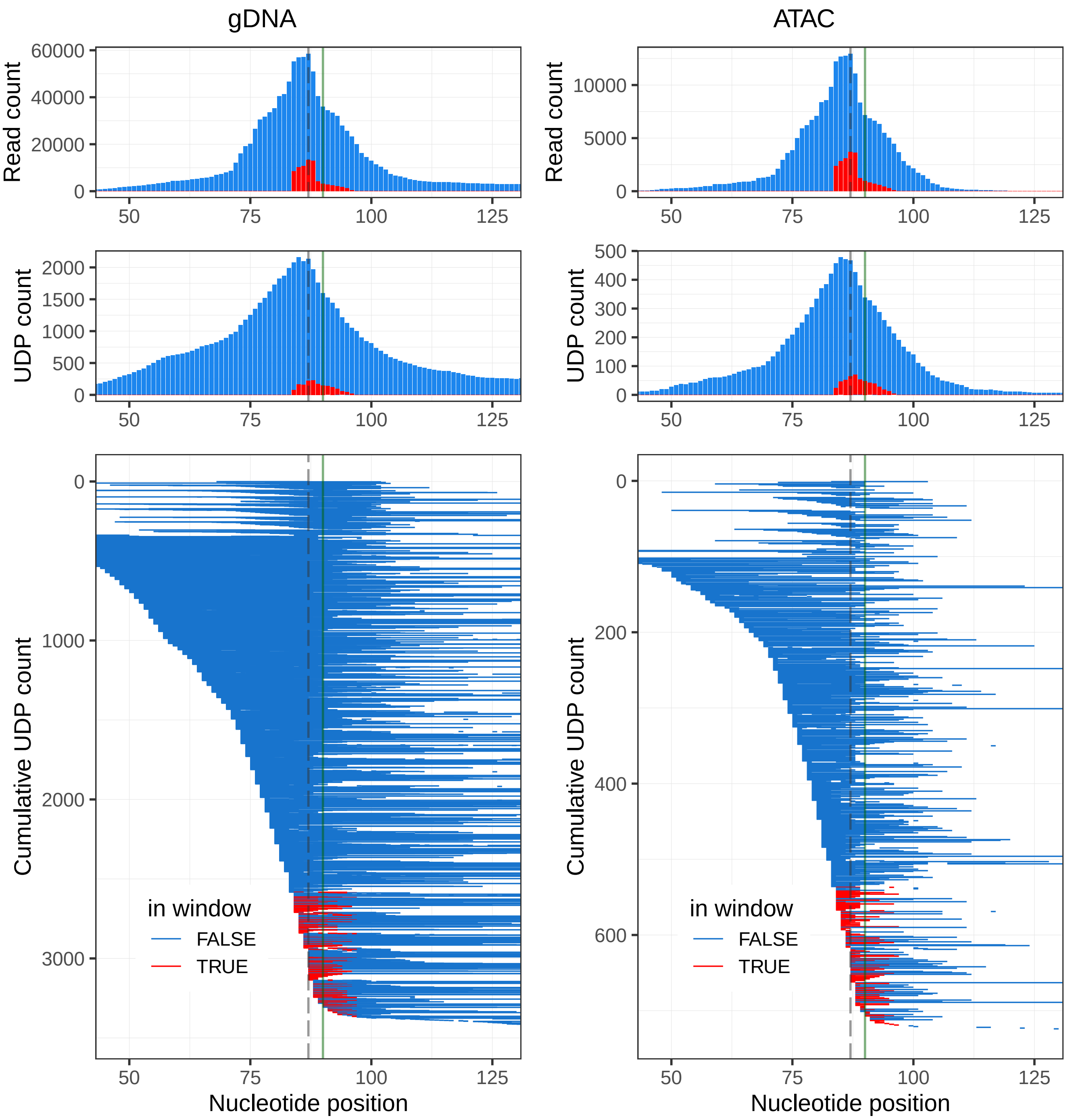

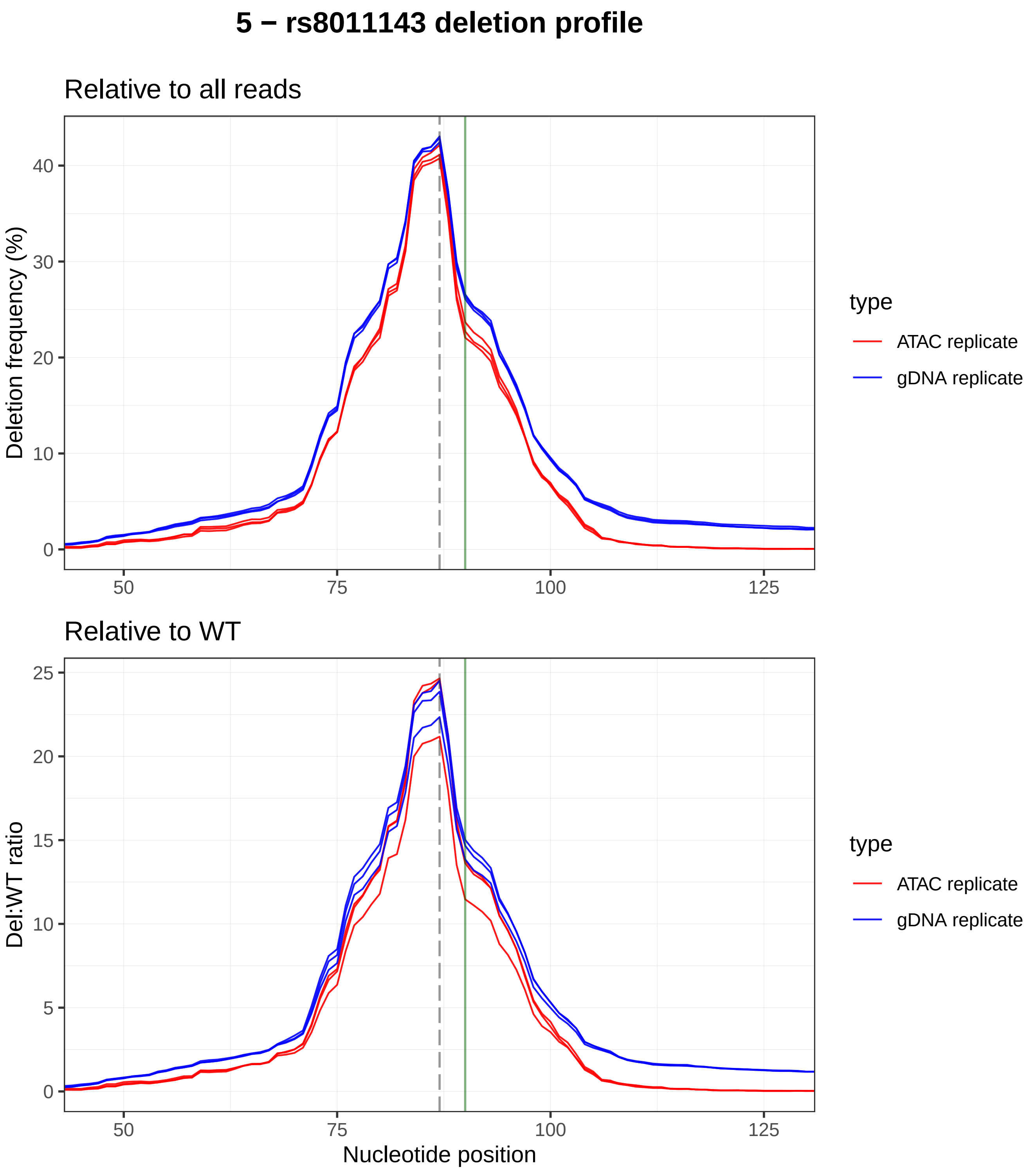
5 − rs8011143 deletion profile
Relative to all reads
Deletion frequency (%)
0
10
20
30
40
50
75
100
125
type
ATAC replicate
gDNA replicate
Relative to WT
Del:WT ratio
0
5
10
15
20
25
50
75
100
125
Nucleotide position
type
ATAC replicate
gDNA replicate

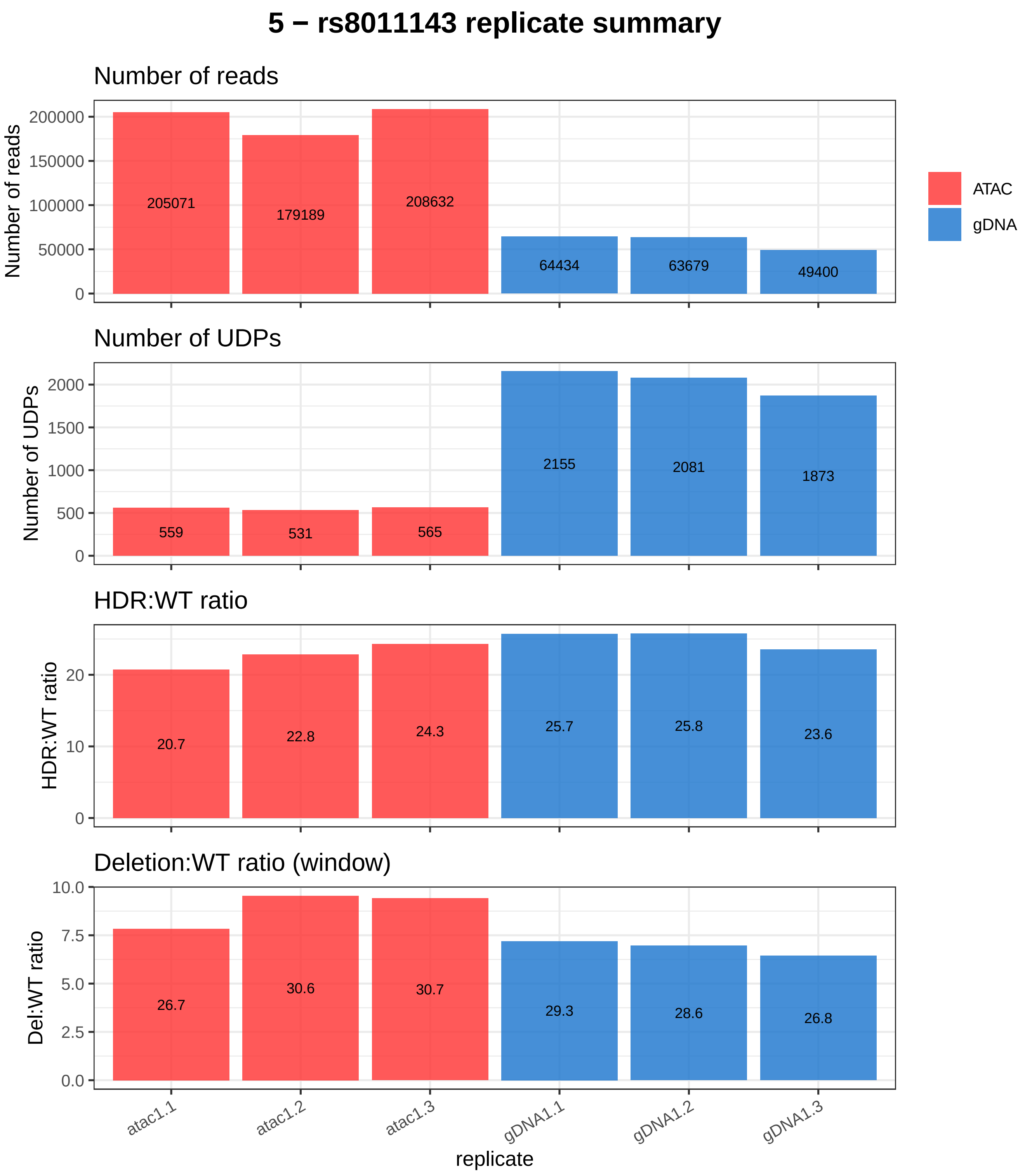
5 − rs8011143 replicate summary
Number of reads
Number of reads
205071
179189
208632
64434
63679
49400
ATAC
gDNA
Number of UDPs
Number of UDPs
559
531
565
2155
2081
1873
HDR:WT ratio
HDR:WT ratio
20.7
22.8
24.3
25.7
25.8
23.6
Deletion:WT ratio (window)
Del:WT ratio
26.7
30.6
30.7
29.3
28.6
26.8
atac1.1
atac1.2
atac1.3
gDNA1.1
gDNA1.2
gDNA1.3
replicate

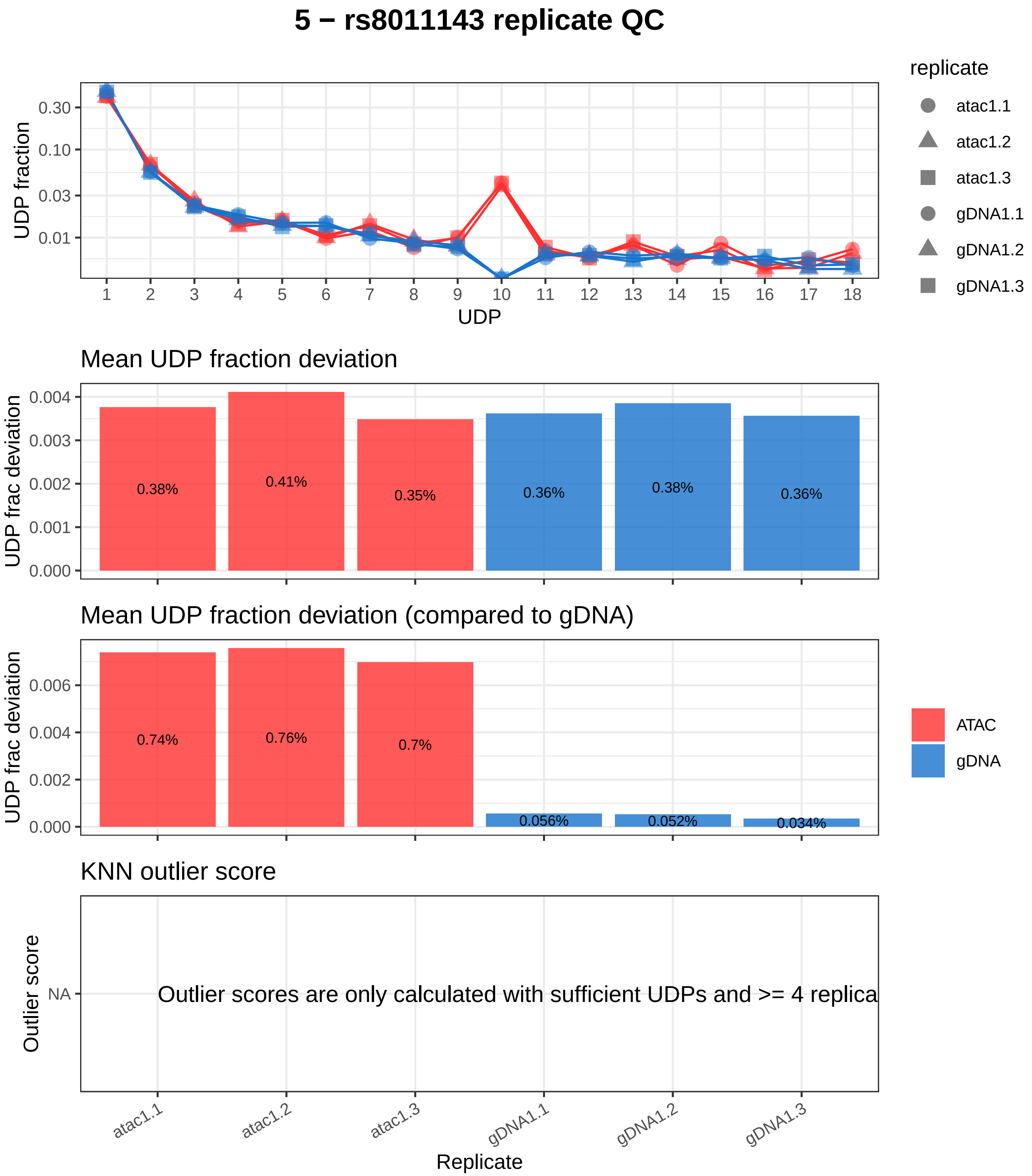

5 − rs8011143 replicate QC
UDP fraction
0.30
0.10
0.03
0.01
UDP
replicate
atac1.1
atac1.2
atac1.3
gDNA1.1
gDNA1.2
gDNA1.3
Mean UDP fraction deviation
UDP frac deviation
0.38%
0.41%
0.35%
0.36%
0.38%
0.36%
Mean UDP fraction deviation (compared to gDNA)
0.74%
0.76%
0.7%
0.056%
0.052%
0.034%
ATAC
gDNA
KNN outlier score
Outlier score
NA
Outlier scores are only calculated with sufficient UDPs and >= 4 replica
Replicate

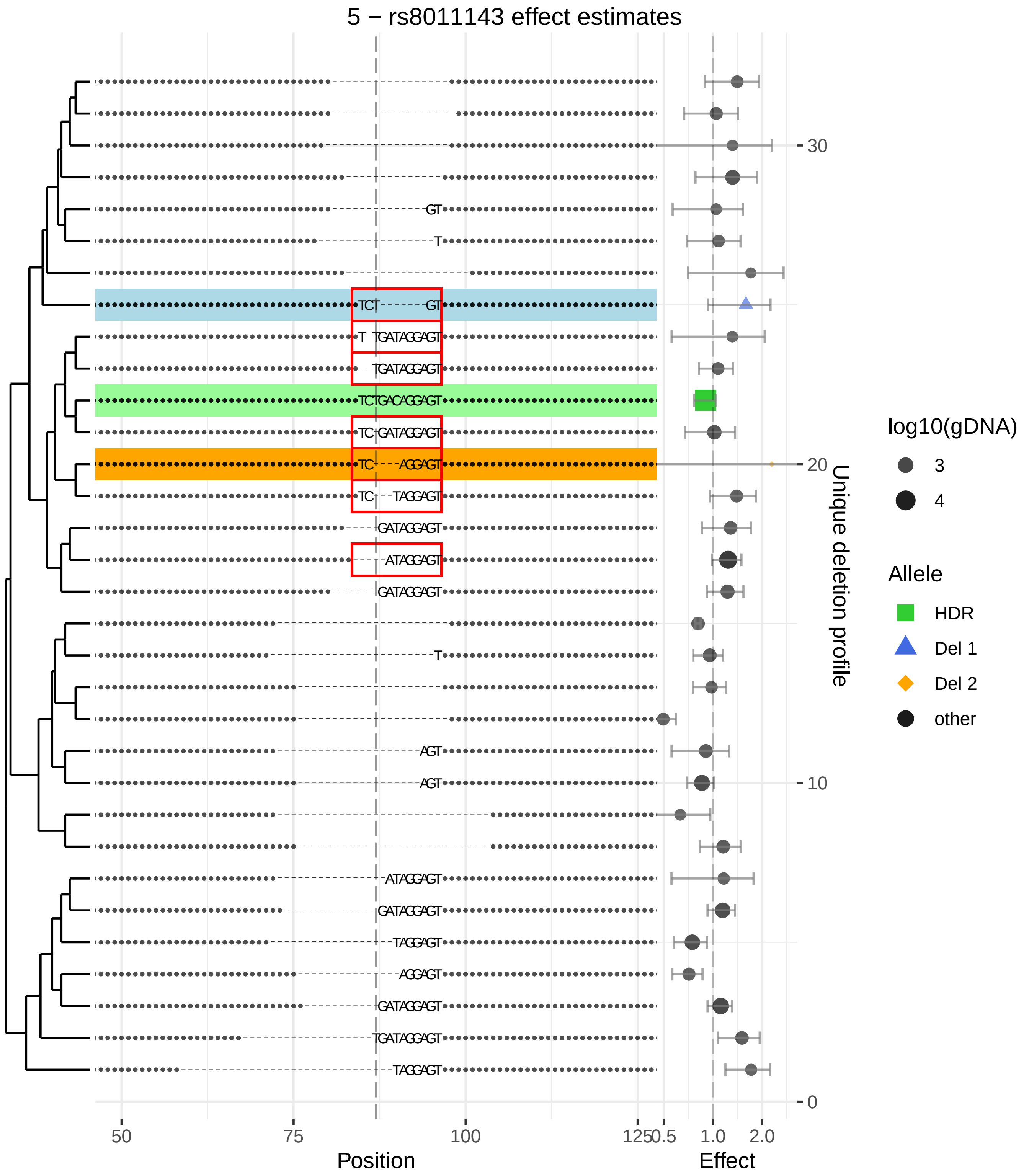
5 − rs8011143 effect estimates
TCT
GT
T
GT
T TGATAGGAGT
TGATAGGAGT
TCTGACAGGAGT
TC GATAGGAGT
TC AGGAGT
TC TAGGAGT
GATAGGAGT
ATAGGAGT
GATAGGAGT
T
AGT
AGT
ATAGGAGT
GATAGGAGT
TAGGAGT
AGGAGT
GATAGGAGT
TGATAGGAGT
TAGGAGT
50
75
100
125
0.5
1.0
2.0
Position
Effect
30
20
10
0
Unique deletion profile
log10(gDNA)
3
4
Allele
HDR
Del 1
Del 2
other

6 − rs4757506 grep summary

Mean HDR frac gDNA: 1.6%, ATAC: 0.36%
Mean WT frac gDNA: 77%, ATAC: 7.9%

ATAC:gDNA ratio (HDR/WT): 2.17
95% CI: (2.07, 2.27),   p = 1.59e−05

replicate	atac1.2	atac1.3	gDNA1.1	gDNA1.2	gDNA1.3
type	ATAC	ATAC	gDNA	gDNA	gDNA
num_reads	176492	171493	60754	70665	53424
HDR reads	653	607	983	1177	846
WT reads	14376	13287	47038	54738	41282
HDR_WT_ratio	0.0454	0.0457	0.0209	0.0215	0.0205
HDR_frac	0.37%	0.35%	1.62%	1.67%	1.58%
WT_frac	8.15%	7.75%	77.42%	77.46%	77.27%

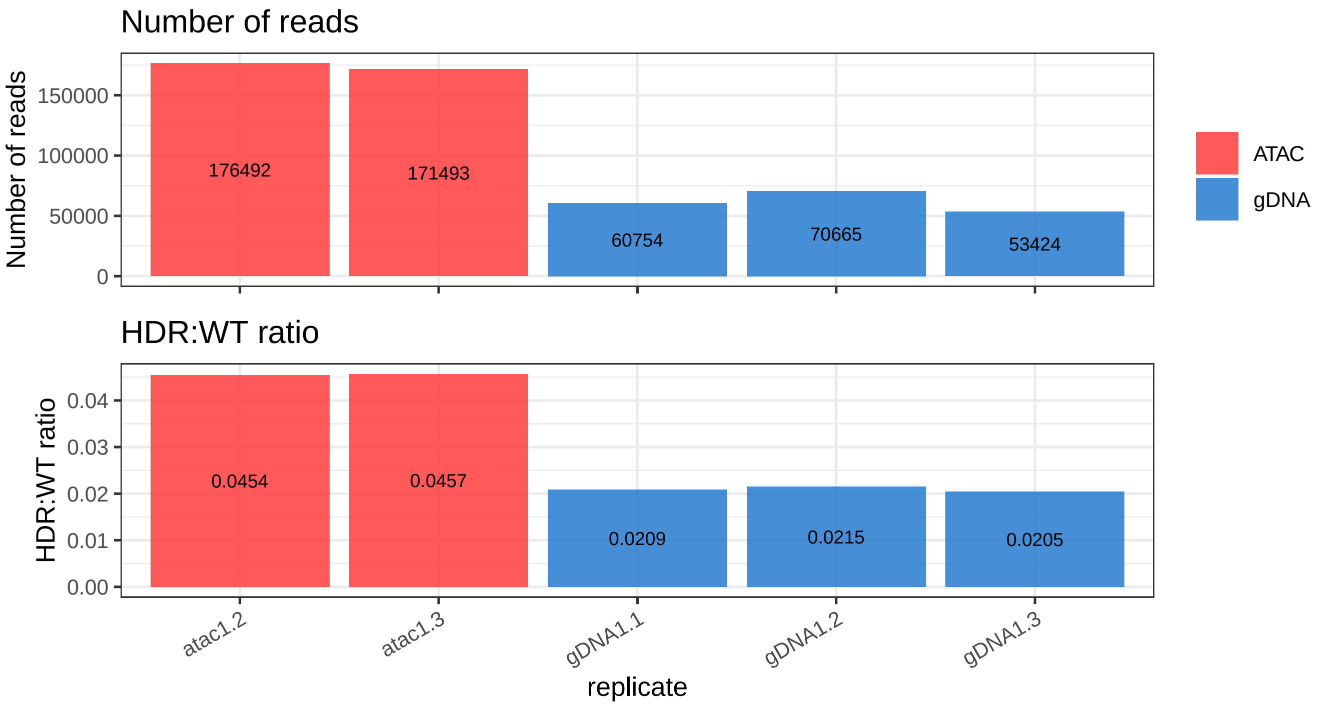

6 − rs4757506 analysis summary

Mean HDR frac gDNA: 1.7%, ATAC: 3.1%
Mean DEL frac gDNA: 34%, ATAC: 14%
Mean WT frac gDNA: 64%, ATAC: 62%

ATAC:gDNA ratio (HDR/WT): 1.88
95% CI: (1.8, 1.96),   p = 1.56e−05

ATAC:gDNA ratio (DEL/WT) [70−82]: 0.498
95% CI: (0.307, 0.688),   p = 0.0235
ATAC:gDNA ratio (DEL/WT) − Del 1: 0.419
95% CI: (0.219, 0.618),   p = 0.00771
ATAC:gDNA ratio (DEL/WT) − Del 2: 0.56
95% CI: (0.21, 0.91),   p = 0.0301

replicate	atac1.2	atac1.3	gDNA1.1	gDNA1.2	gDNA1.3
type	ATAC	ATAC	gDNA	gDNA	gDNA
num_udps	429	404	1168	1213	1063
HDR_WT_ratio	0.0504	0.0508	0.0268	0.0276	0.0264
DEL_WT_ratio	0.222	0.238	0.528	0.517	0.529
HDR_rate	3.16%	3.13%	1.71%	1.77%	1.68%
DEL_rate	13.91%	14.70%	33.67%	33.20%	33.75%
editing_rate	17.07%	17.84%	35.38%	34.97%	35.43%
WT_rate	62.57%	61.66%	63.78%	64.17%	63.81%
num_reads	176492	171493	60754	70665	53424
HDR reads	572	530	814	973	699
WT reads	11338	10426	30384	35301	26503
Deletion reads	2521	2486	16039	18264	14019
excluded−insertion	2476	2275	12652	14952	11277
excluded−minoverlap	1	0	0	0	0
excluded−mismatches	1	3	1	0	0
excluded−nonspanning	155894	152307	461	703	610
excluded−mult.deletions	0	0	0	0	0

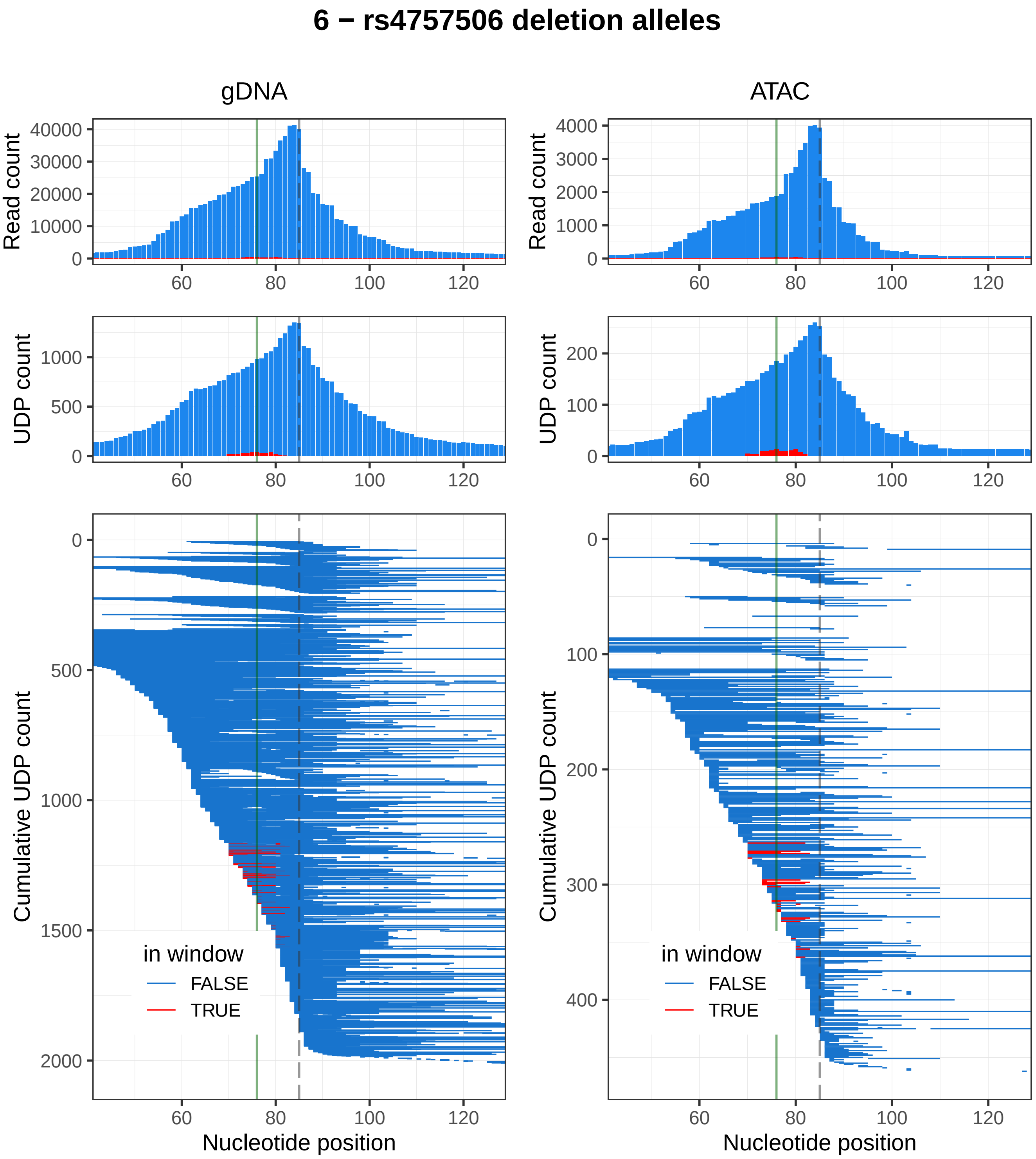
6 − rs4757506 deletion alleles
gDNA
ATAC
Read count
UDP count
Cumulative UDP count
Nucleotide position
in window
FALSE
TRUE
40000
30000
20000
10000
0
4000
3000
2000
1000
1000
500
200
100
1500
2000
400
300
60
80
100
120

6 − rs4757506 deletion profile

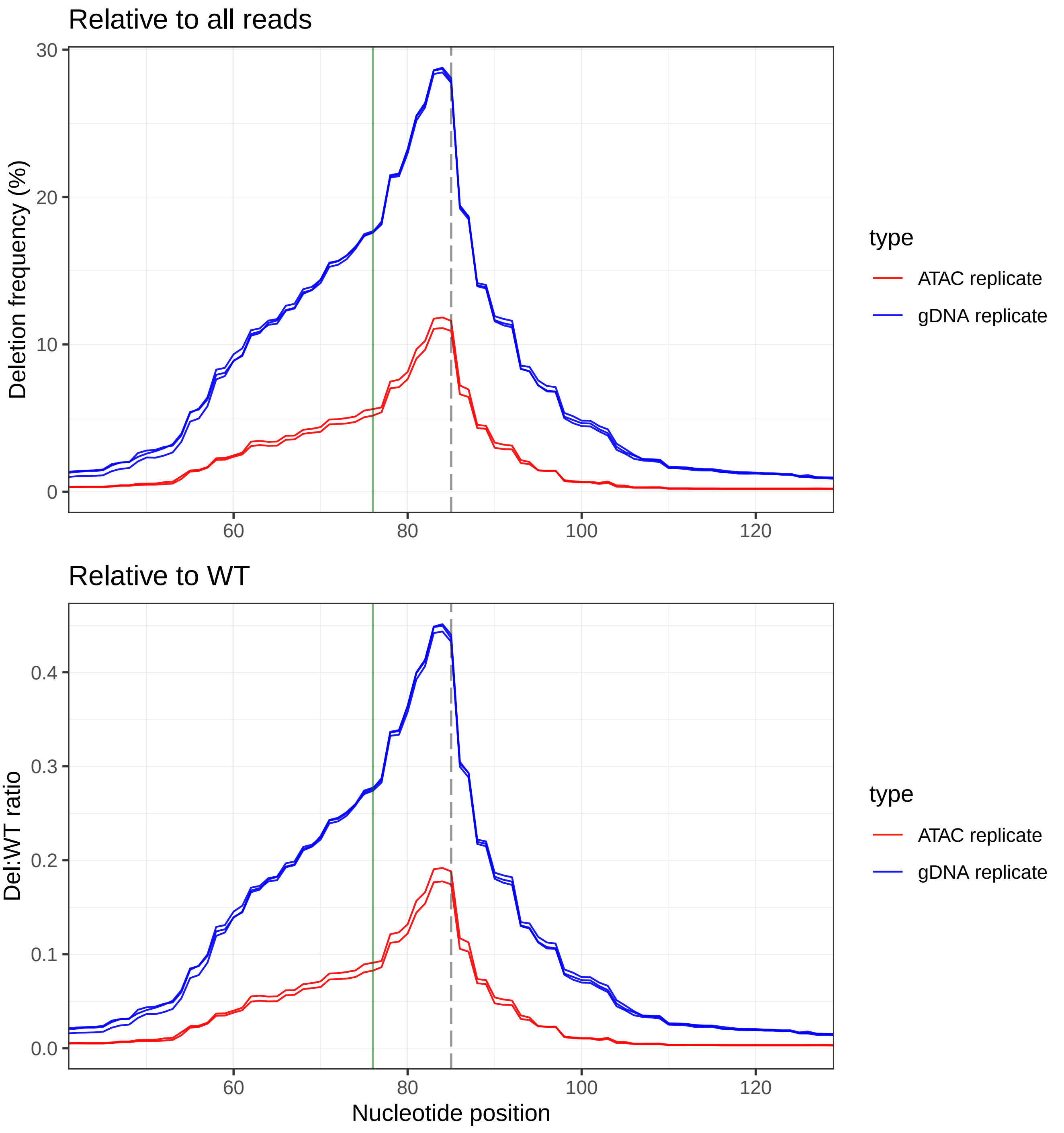

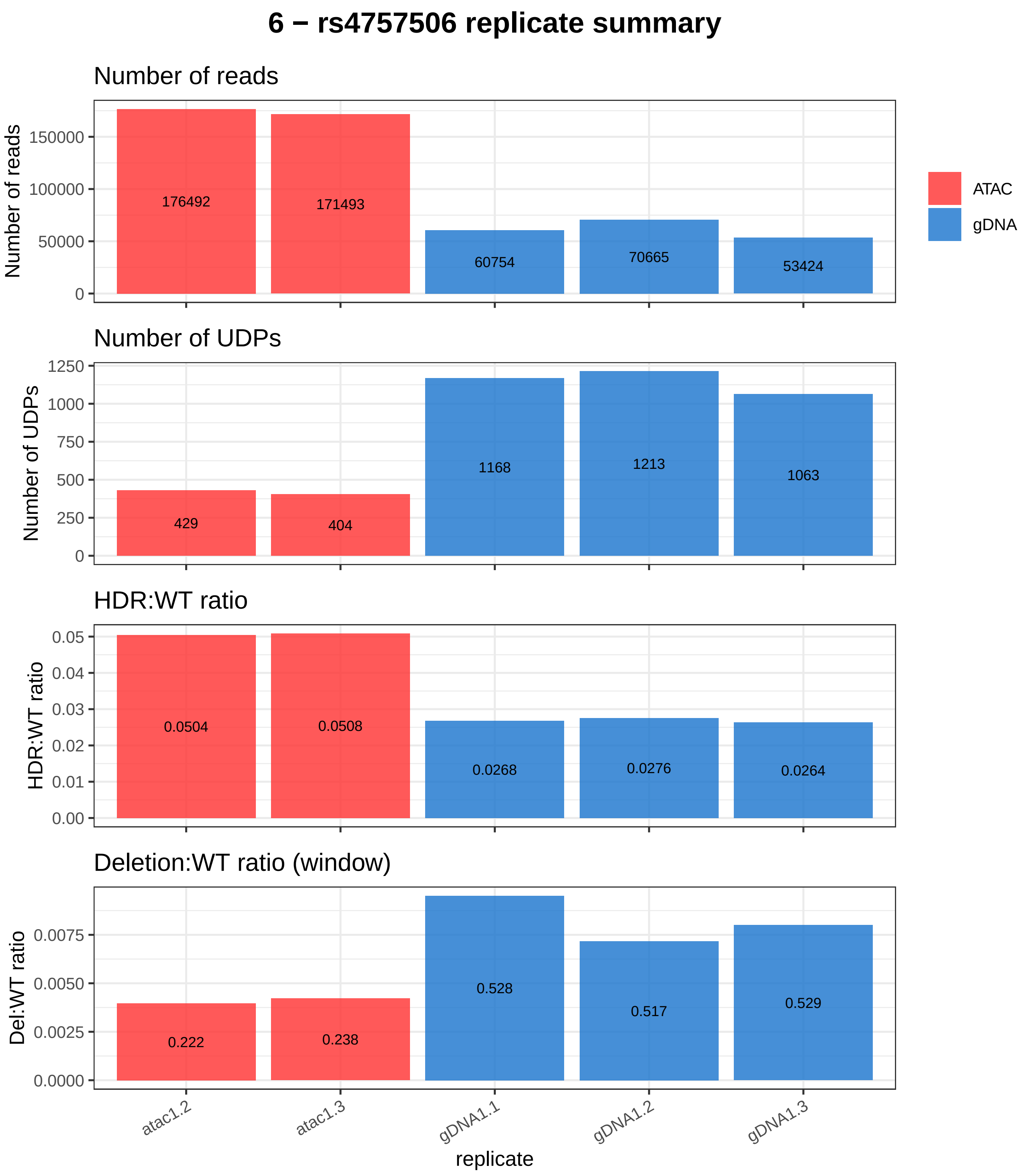
6 − rs4757506 replicate summary
Number of reads
Number of reads
0
50000
100000
150000
176492
171493
60754
70665
53424
ATAC
gDNA
Number of UDPs
Number of UDPs
0
250
500
750
1000
1250
429
404
1168
1213
1063
HDR:WT ratio
HDR:WT ratio
0.00
0.01
0.02
0.03
0.04
0.05
0.0504
0.0508
0.0268
0.0276
0.0264
Deletion:WT ratio (window)
Del:WT ratio
0.0000
0.0025
0.0050
0.0075
0.222
0.238
0.528
0.517
0.529
atac1.2
atac1.3
gDNA1.1
gDNA1.2
gDNA1.3
replicate

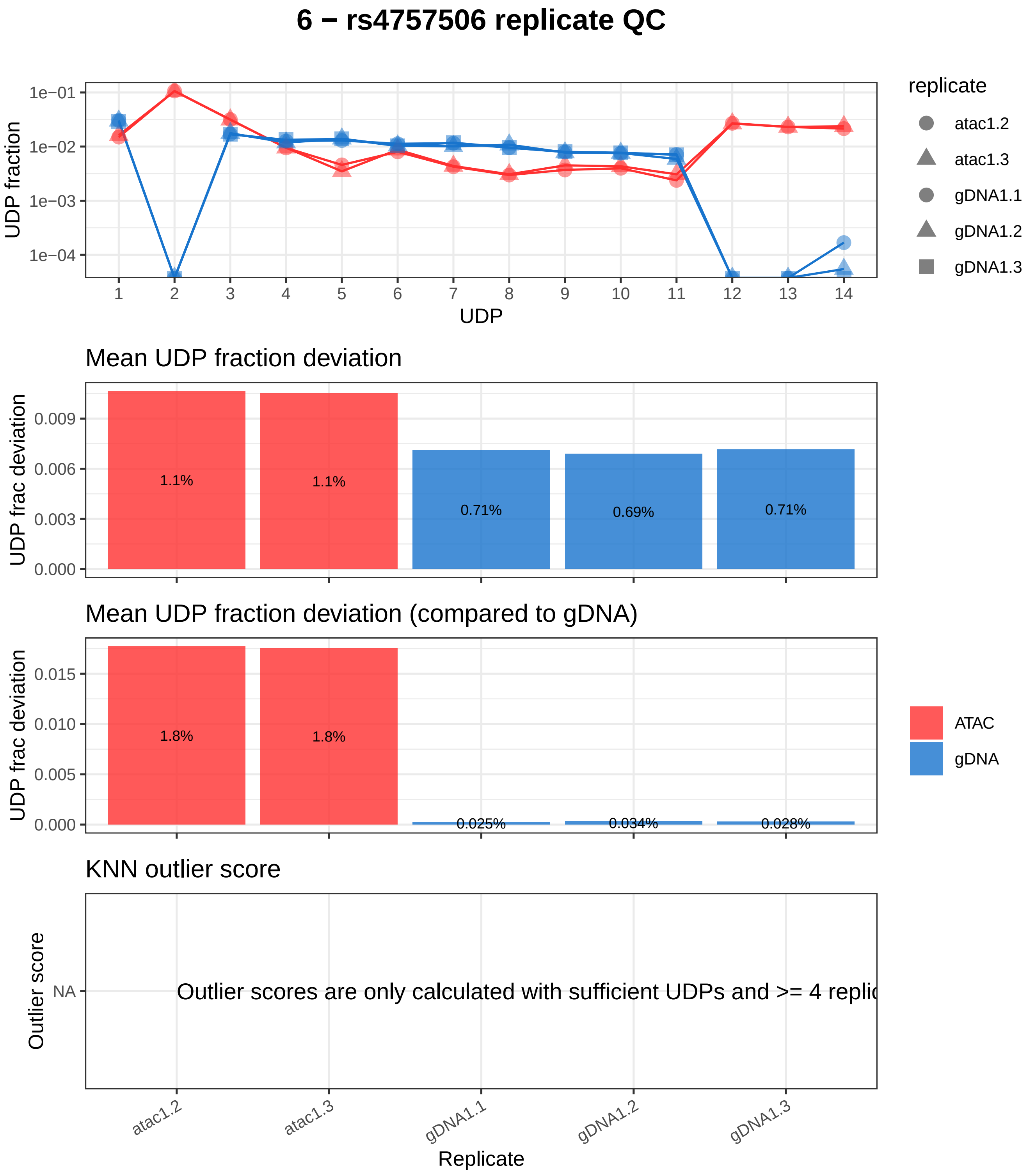

6 − rs4757506 replicate QC
UDP fraction
1e−01
1e−02
1e−03
1e−04
1 2 3 4 5 6 7 8 9 10 11 12 13 14
UDP
replicate
atac1.2
atac1.3
gDNA1.1
gDNA1.2
gDNA1.3
Mean UDP fraction deviation
UDP frac deviation
0.009
0.006
0.003
0.000
1.1%
1.1%
0.71%
0.69%
0.71%
Mean UDP fraction deviation (compared to gDNA)
UDP frac deviation
0.015
0.010
0.005
0.000
1.8%
1.8%
0.025%
0.034%
0.028%
ATAC
gDNA
KNN outlier score
Outlier score
NA
Outlier scores are only calculated with sufficient UDPs and >= 4 replic
atac1.2
atac1.3
gDNA1.1
gDNA1.2
gDNA1.3
Replicate

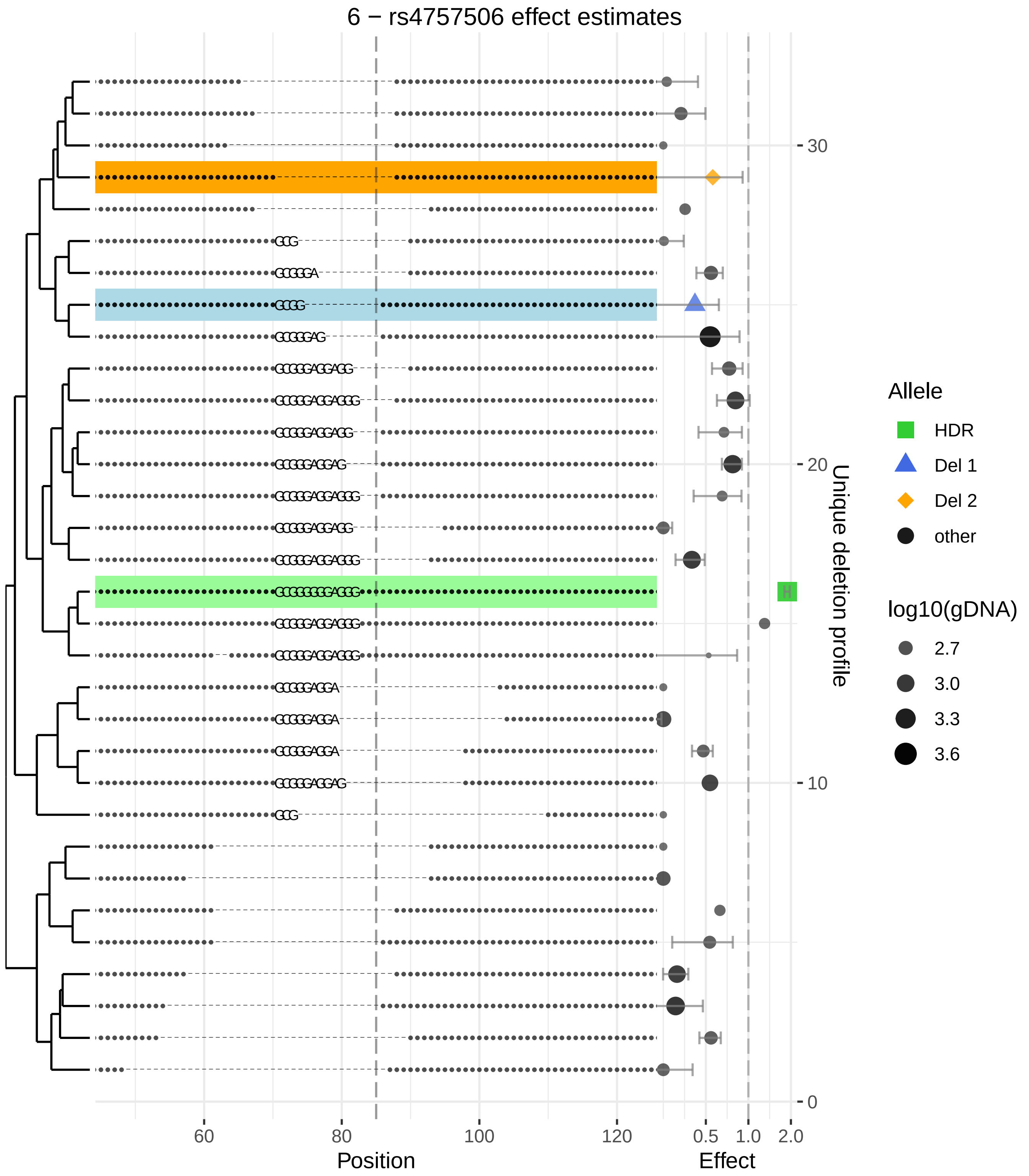

6 − rs4757506 effect estimates
Position
Effect
Unique deletion profile
Allele
HDR
Del 1
Del 2
other
log10(gDNA)
2.7
3.0
3.3
3.6
60
80
100
120
0.5
1.0
2.0
0
10
20
30

7 − rs185220 grep summary

Mean HDR frac gDNA: 3.3%, ATAC: 0.65%
Mean WT frac gDNA: 54%, ATAC: 9.7%

ATAC:gDNA ratio (HDR/WT): 1.09
95% CI: (1.03, 1.14),   p = 0.0125

replicate	atac1.1	atac1.2	atac1.3	gDNA1.1	gDNA1.2	gDNA1.3
type	ATAC	ATAC	ATAC	gDNA	gDNA	gDNA
num_reads	187140	162015	141124	52084	51371	42466
HDR reads	1200	1086	892	1775	1676	1421
WT reads	17915	15874	13646	28436	27524	23149
HDR_WT_ratio	0.067	0.0684	0.0654	0.0624	0.0609	0.0614
HDR_frac	0.64%	0.67%	0.63%	3.41%	3.26%	3.35%
WT_frac	9.57%	9.80%	9.67%	54.60%	53.58%	54.51%

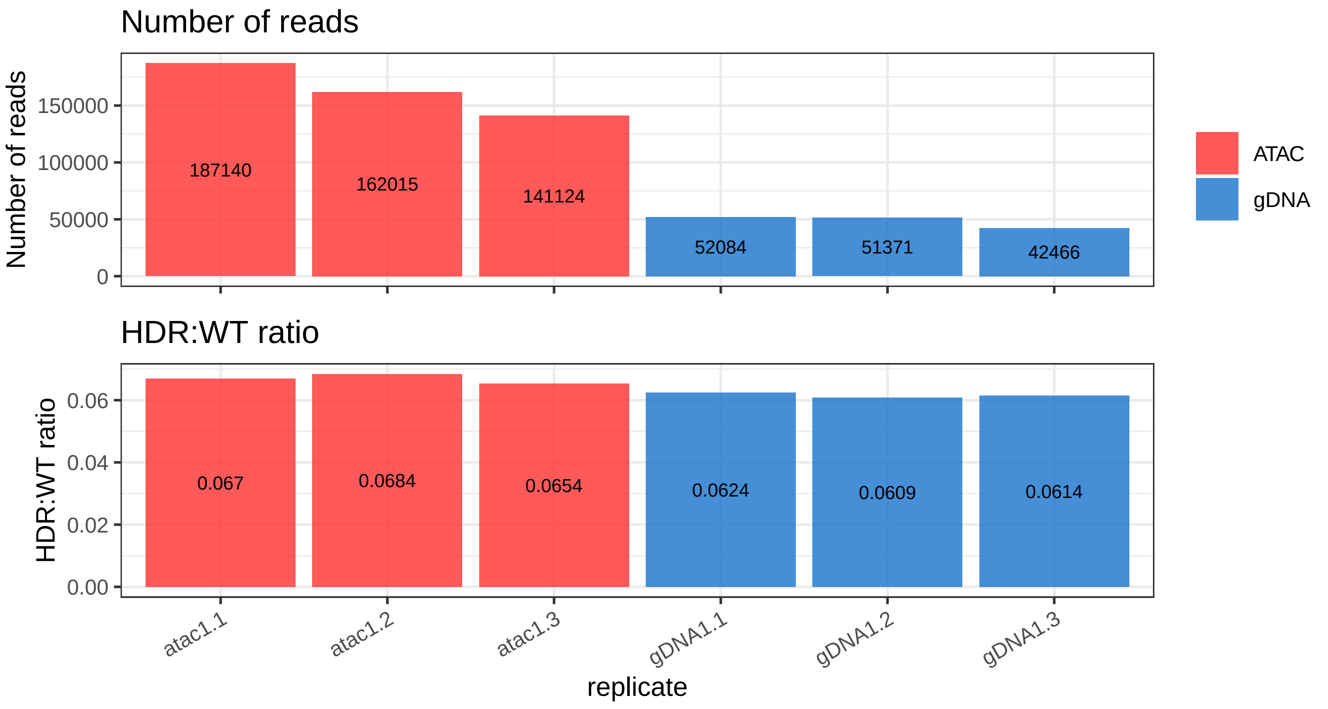

7 − rs185220 analysis summary

Mean HDR frac gDNA: 1.9%, ATAC: 3.3%
Mean DEL frac gDNA: 38%, ATAC: 31%
Mean WT frac gDNA: 59%, ATAC: 62%

ATAC:gDNA ratio (HDR/WT): 1.63
95% CI: (1.43, 1.83),   p = 0.00246

ATAC:gDNA ratio (DEL/WT) [73−85]: 1.21
95% CI: (1.16, 1.27),   p = 0.000554
ATAC:gDNA ratio (DEL/WT) − Del 1: 1.2
95% CI: (1.12, 1.28),   p = 0.00461
ATAC:gDNA ratio (DEL/WT) − Del 2: 1.49
95% CI: (1.42, 1.55),   p = 0.000443

replicate	atac1.1	atac1.2	atac1.3	gDNA1.1	gDNA1.2	gDNA1.3
type	ATAC	ATAC	ATAC	gDNA	gDNA	gDNA
num_udps	1001	968	857	2431	2347	2150
HDR_WT_ratio	0.0548	0.0551	0.0509	0.0335	0.0329	0.0321
DEL_WT_ratio	0.51	0.509	0.501	0.657	0.66	0.644
HDR_rate	3.39%	3.41%	3.18%	1.96%	1.93%	1.90%
DEL_rate	31.49%	31.52%	31.36%	38.55%	38.66%	38.10%
editing_rate	34.88%	34.94%	34.54%	40.51%	40.59%	40.00%
WT_rate	61.78%	61.90%	62.61%	58.66%	58.60%	59.17%
num_reads	187140	162015	141124	52084	51371	42466
HDR reads	966	863	685	933	888	728
WT reads	17624	15652	13468	27884	26968	22677
Deletion reads	8984	7971	6746	18324	17790	14603
excluded−insertion	2293	2053	1733	4199	4192	3579
excluded−minoverlap	1	1	0	0	0	0
excluded−mismatches	3	3	4	16	12	7
excluded−nonspanning	156314	134673	117875	335	1146	552
excluded−mult.deletions	0	0	0	0	0	0

7 − rs185220 deletion alleles

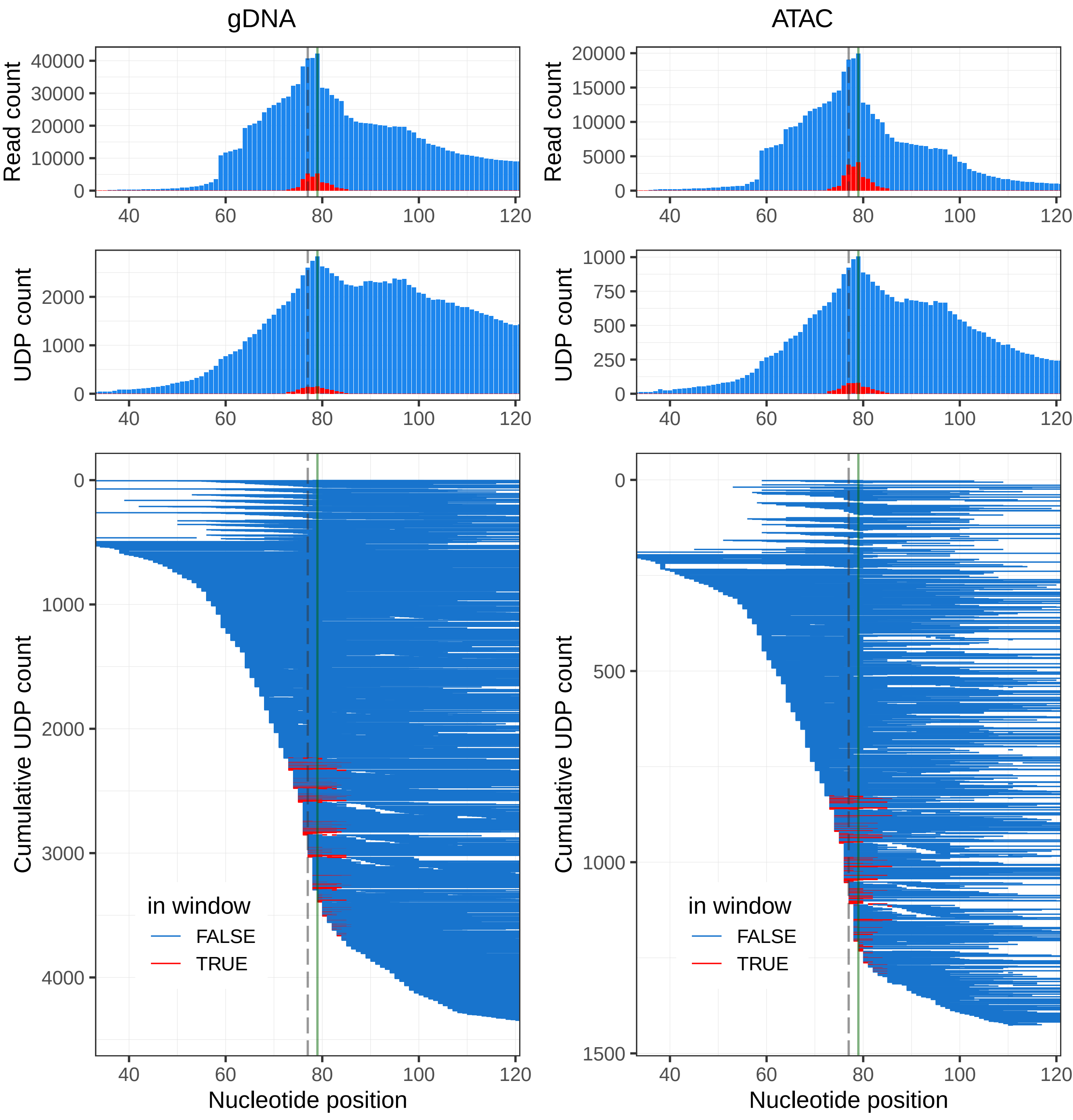

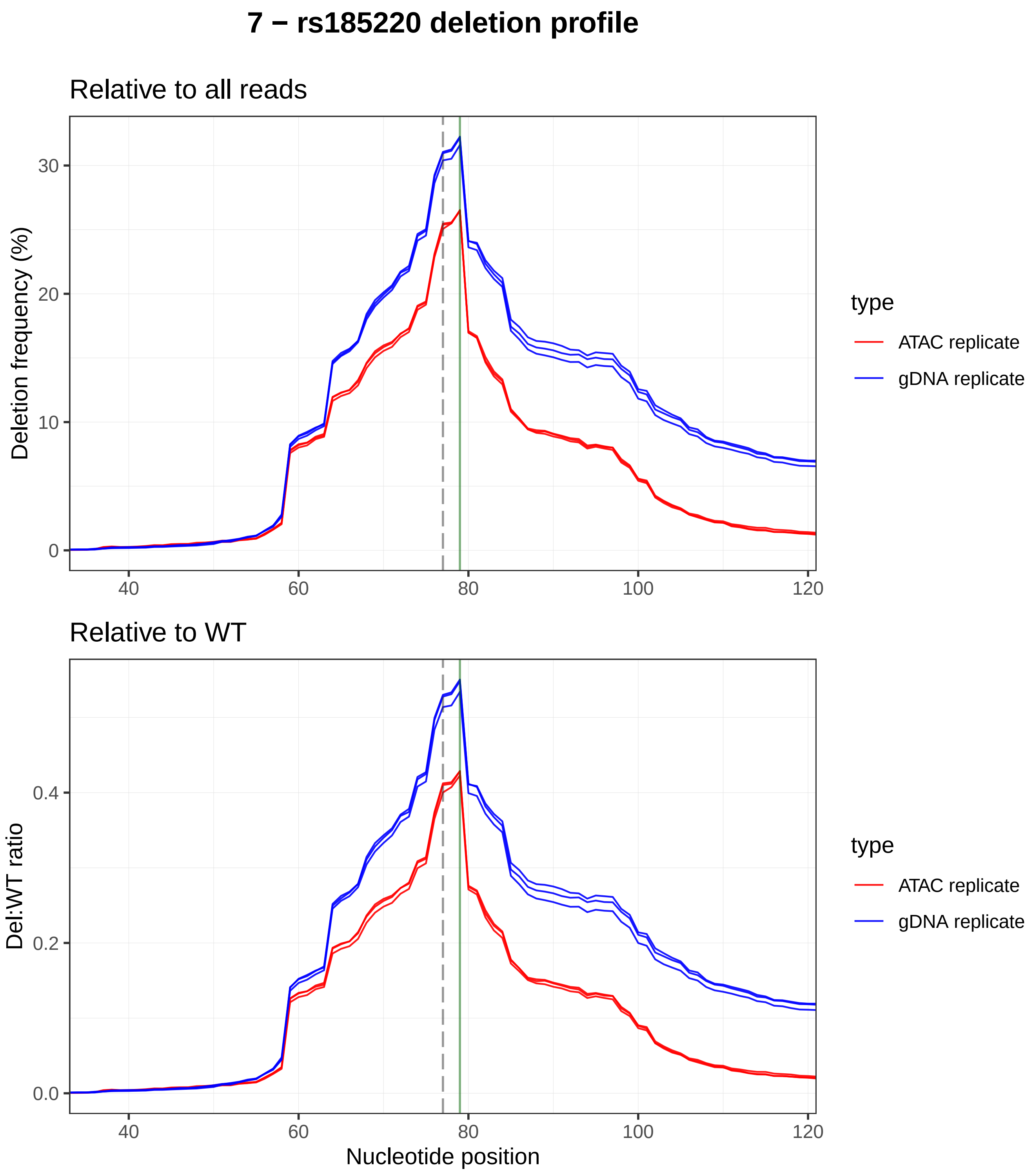
7 – rs185220 deletion profile
Relative to all reads
Deletion frequency (%)
0
10
20
30
40
60
80
100
120
type
ATAC replicate
gDNA replicate
Relative to WT
Del:WT ratio
0.0
0.2
0.4
Nucleotide position

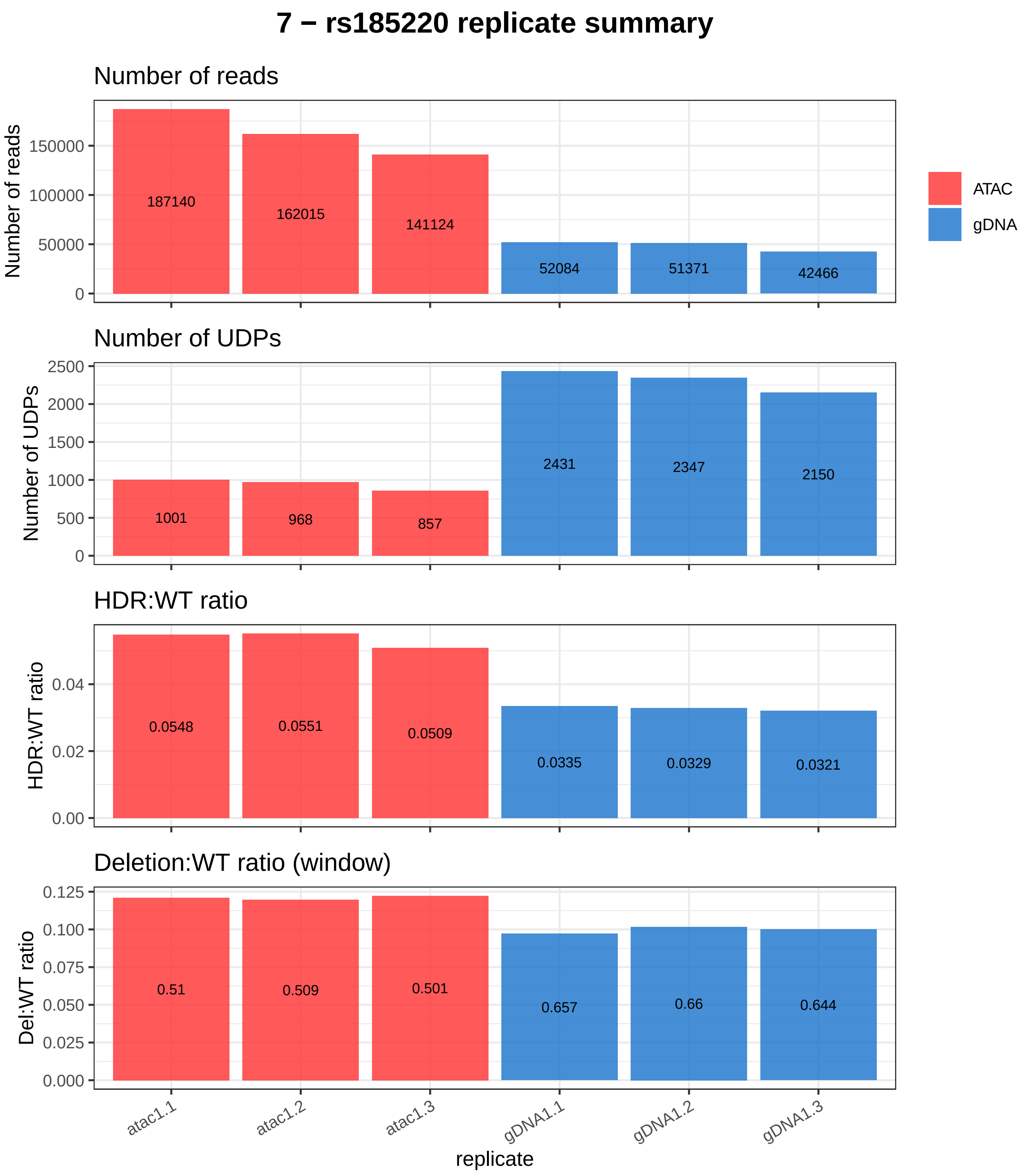
7 − rs185220 replicate summary
Number of reads
Number of reads
150000
100000
50000
0
187140
162015
141124
52084
51371
42466
ATAC
gDNA
Number of UDPs
Number of UDPs
2500
2000
1500
1000
500
0
1001
968
857
2431
2347
2150
HDR:WT ratio
HDR:WT ratio
0.04
0.02
0.00
0.0548
0.0551
0.0509
0.0335
0.0329
0.0321
Deletion:WT ratio (window)
Del:WT ratio
0.125
0.100
0.075
0.050
0.025
0.000
0.51
0.509
0.501
0.657
0.66
0.644
atac1.1
atac1.2
atac1.3
gDNA1.1
gDNA1.2
gDNA1.3
replicate

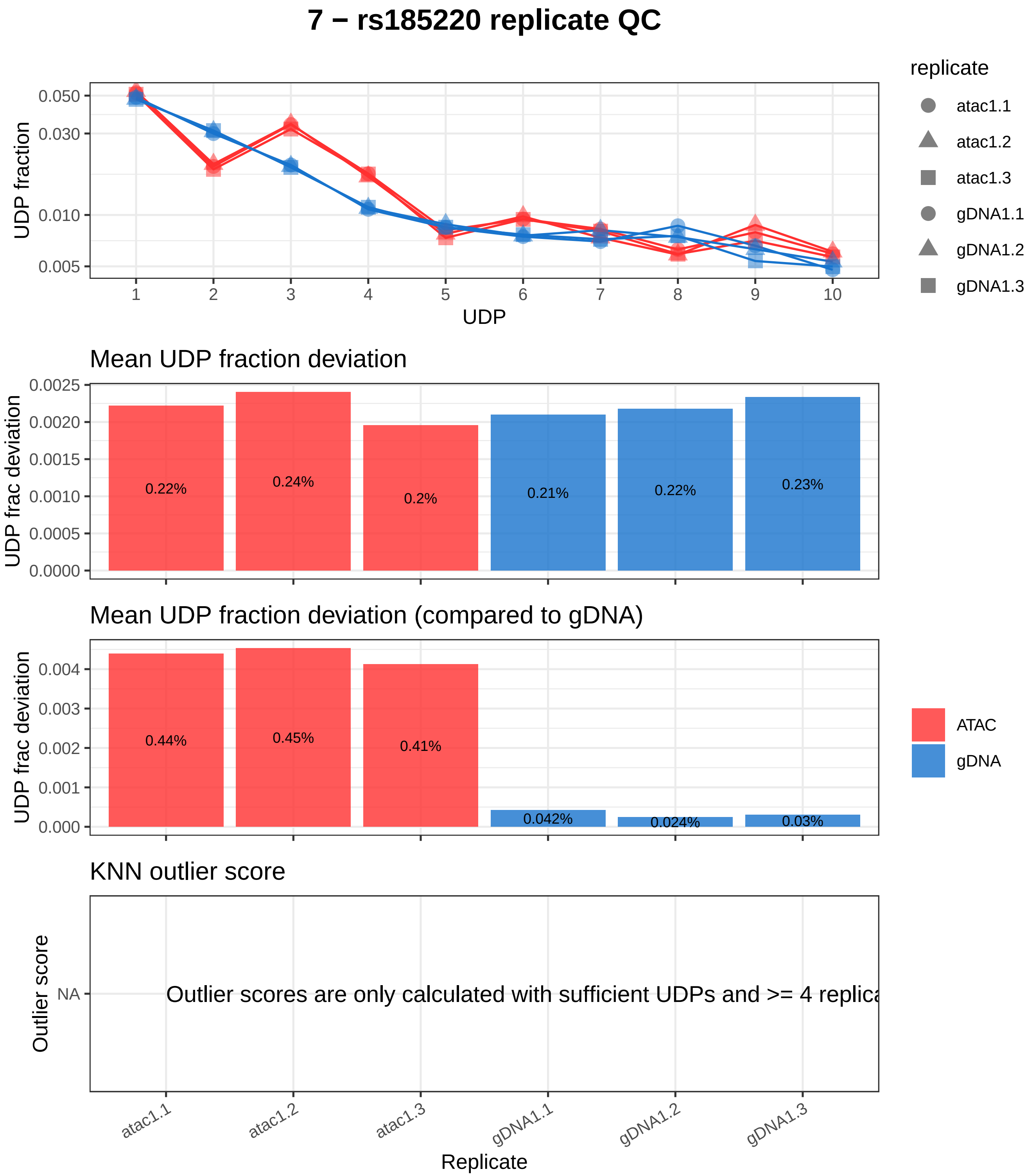

7 − rs185220 replicate QC
UDP fraction
0.050
0.030
0.010
0.005
1
2
3
4
5
6
7
8
9
10
UDP
replicate
atac1.1
atac1.2
atac1.3
gDNA1.1
gDNA1.2
gDNA1.3
Mean UDP fraction deviation
UDP frac deviation
0.0025
0.0020
0.0015
0.0010
0.0005
0.0000
0.22%
0.24%
0.2%
0.21%
0.22%
0.23%
Mean UDP fraction deviation (compared to gDNA)
0.004
0.003
0.002
0.001
0.000
0.44%
0.45%
0.41%
0.042%
0.024%
0.03%
ATAC
gDNA
KNN outlier score
Outlier score
NA
Outlier scores are only calculated with sufficient UDPs and >= 4 replica
atac1.1
atac1.2
atac1.3
gDNA1.1
gDNA1.2
gDNA1.3
Replicate

7 − rs185220 effect estimates

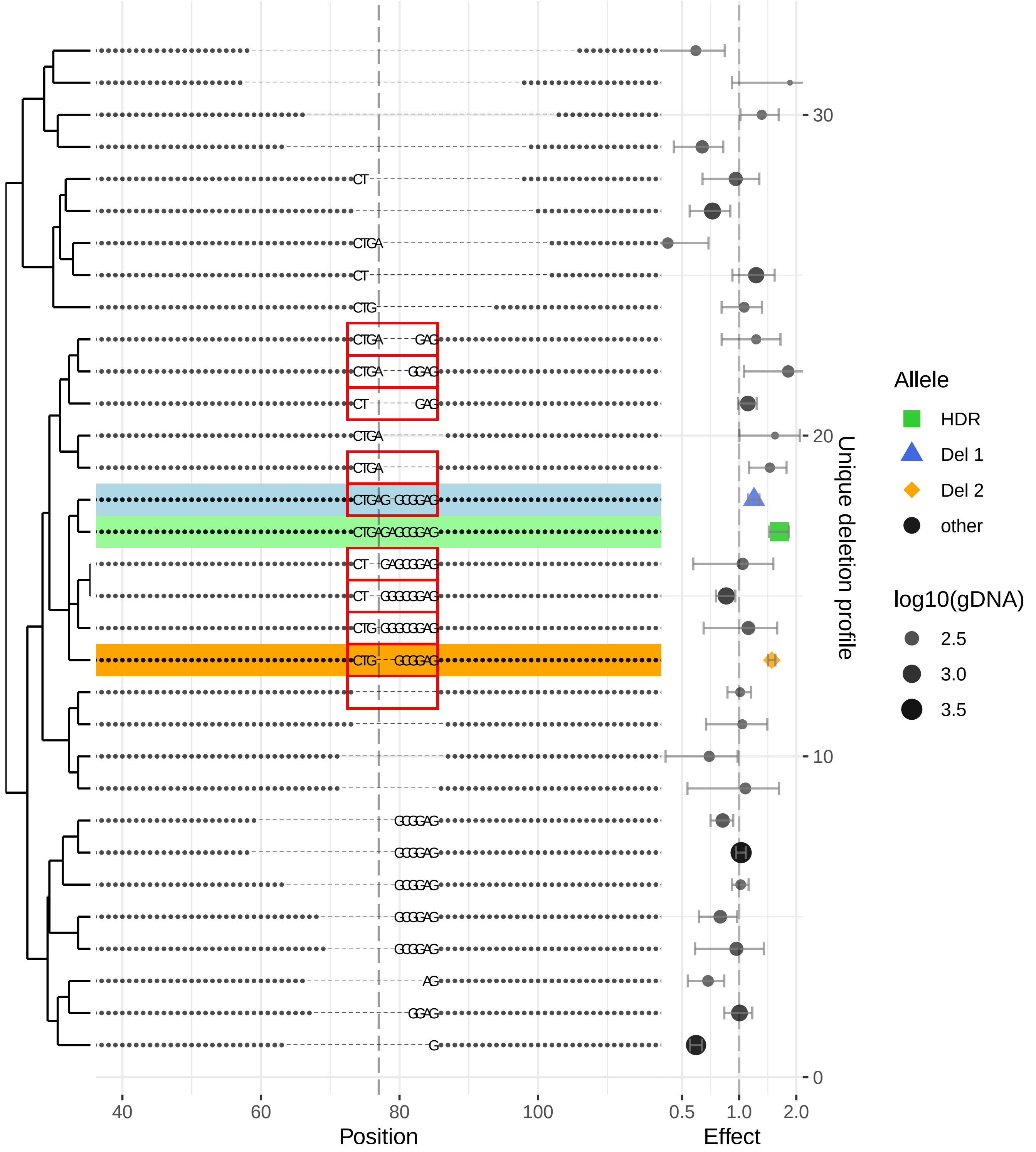

8 − rs141252451 grep summary

Mean HDR frac gDNA: 4.4%, ATAC: 0.19%
Mean WT frac gDNA: 31%, ATAC: 6.2%

ATAC:gDNA ratio (HDR/WT): 0.218
95% CI: (0.185, 0.252),   p = 7.83e−06

replicate	atac1.1	atac1.2	atac1.3	gDNA1.1	gDNA1.2	gDNA1.3
type	ATAC	ATAC	ATAC	gDNA	gDNA	gDNA
num_reads	208522	218853	213910	55110	94991	49930
HDR reads	440	417	374	2505	4055	2248
WT reads	13112	13502	13056	17186	29328	15812
HDR_WT_ratio	0.0336	0.0309	0.0286	0.146	0.138	0.142
HDR_frac	0.21%	0.19%	0.17%	4.55%	4.27%	4.50%
WT_frac	6.29%	6.17%	6.10%	31.18%	30.87%	31.67%

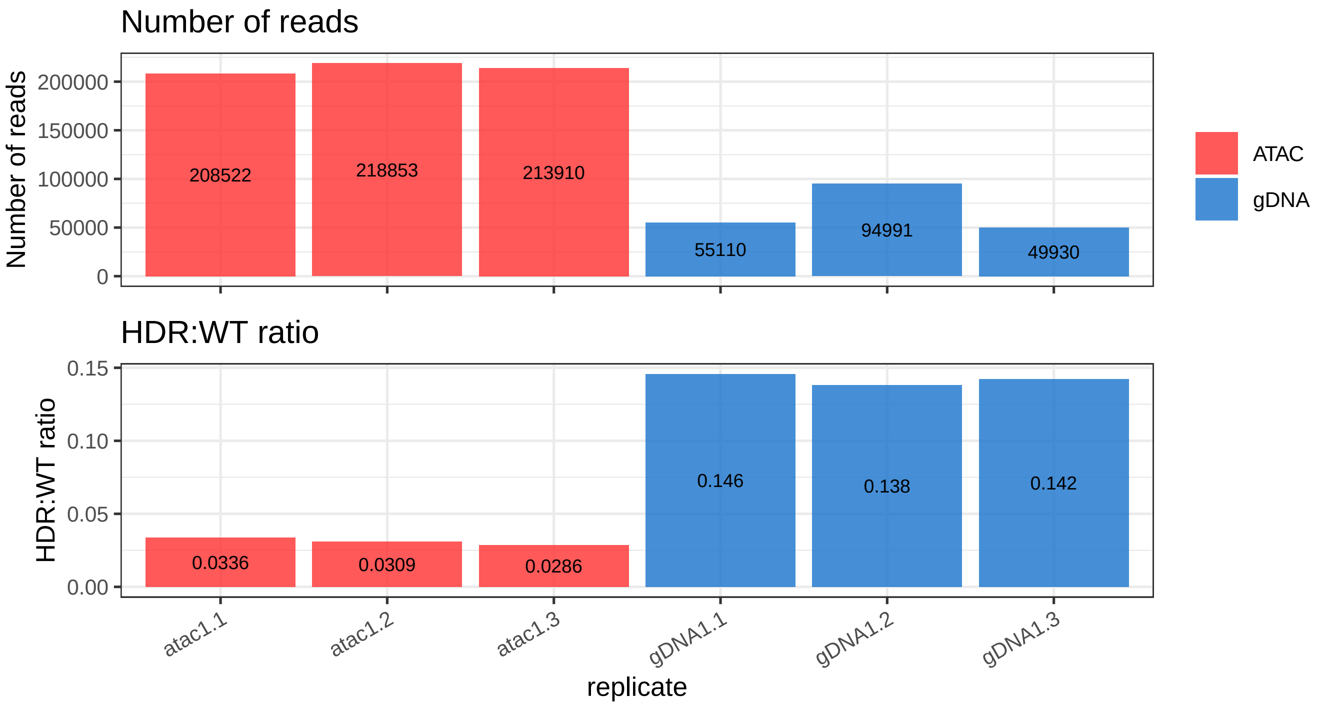

8 − rs141252451 analysis summary

Mean HDR frac gDNA: 4.1%, ATAC: 1.3%
Mean DEL frac gDNA: 63%, ATAC: 37%
Mean WT frac gDNA: 32%, ATAC: 37%

ATAC:gDNA ratio (HDR/WT): 0.287
95% CI: (0.248, 0.325),   p = 3.82e−05

ATAC:gDNA ratio (DEL/WT) [79−91]: 0.842
95% CI: (0.81, 0.873),   p = 0.000327
ATAC:gDNA ratio (DEL/WT) − Del 1: 0.818
95% CI: (0.746, 0.89),   p = 0.00416
ATAC:gDNA ratio (DEL/WT) − Del 2: 0.75
95% CI: (0.696, 0.803),   p = 0.001

replicate	atac1.1	atac1.2	atac1.3	gDNA1.1	gDNA1.2	gDNA1.3
type	ATAC	ATAC	ATAC	gDNA	gDNA	gDNA
num_udps	361	348	360	1606	2029	1549
HDR_WT_ratio	0.0384	0.036	0.0337	0.129	0.121	0.126
DEL_WT_ratio	0.991	0.955	0.993	1.95	1.99	1.92
HDR_rate	1.42%	1.36%	1.25%	4.18%	3.87%	4.13%
DEL_rate	36.75%	36.03%	36.97%	63.12%	63.72%	62.69%
editing_rate	38.17%	37.38%	38.23%	67.30%	67.59%	66.82%
WT_rate	37.07%	37.72%	37.23%	32.34%	31.94%	32.71%
num_reads	208522	218853	213910	55110	94991	49930
HDR reads	408	394	358	2206	3522	1972
WT reads	10629	10950	10627	17054	29053	15633
Deletion reads	10536	10457	10554	33279	57960	29958
excluded−insertion	471	501	457	2300	3849	2045
excluded−minoverlap	0	2	0	0	0	0
excluded−mismatches	0	1	2	0	6	6
excluded−nonspanning	179378	189323	184906	83	179	91
excluded−mult.deletions	0	0	0	0	0	0

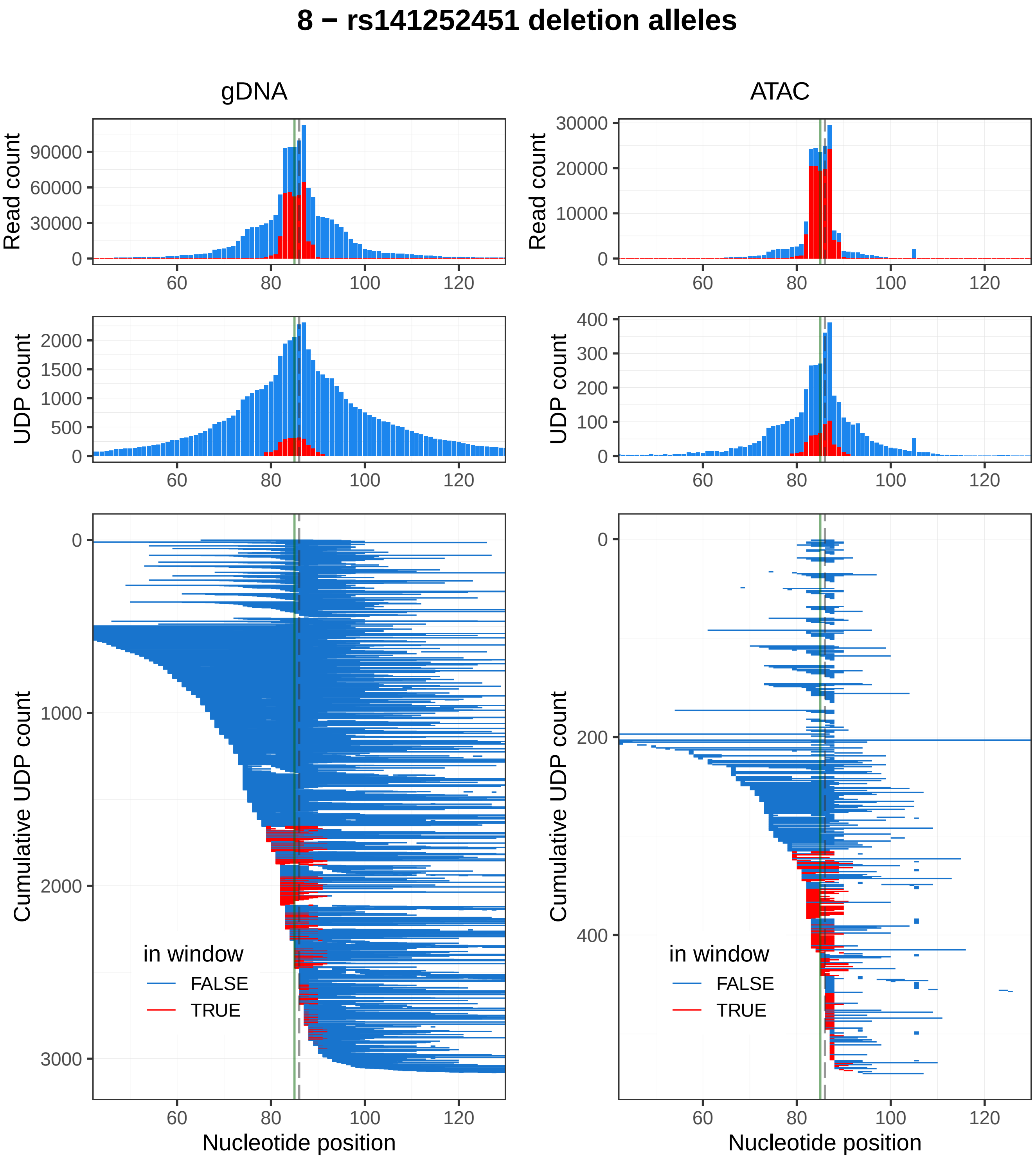

8 − rs141252451 deletion alleles
gDNA
ATAC
Read count
UDP count
Cumulative UDP count
Nucleotide position
in window
FALSE
TRUE

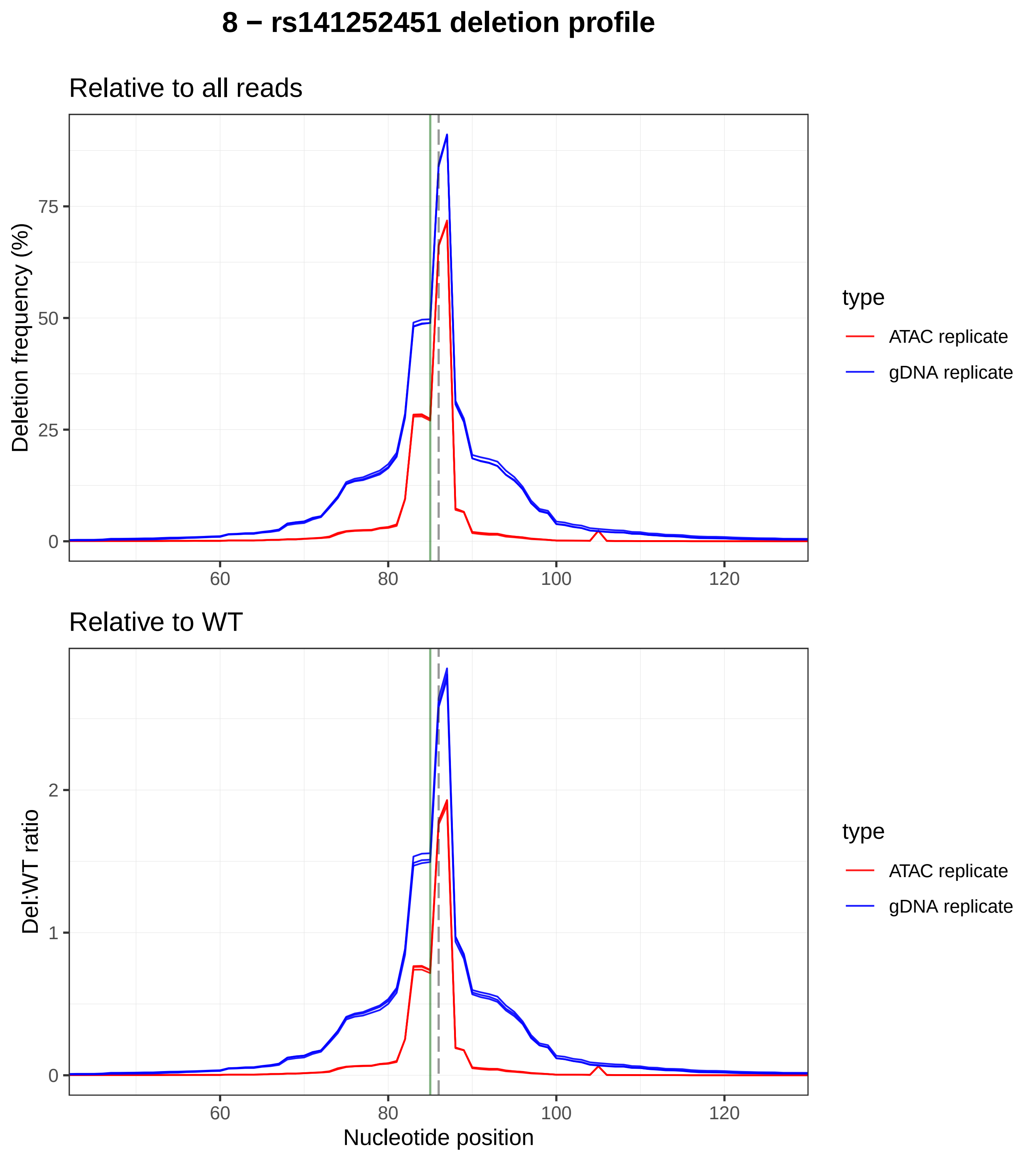
8 − rs141252451 deletion profile
Relative to all reads
Deletion frequency (%)
0
25
50
75
60
80
100
120
type
ATAC replicate
gDNA replicate
Relative to WT
Del:WT ratio
0
1
2
60
80
100
120
Nucleotide position
type
ATAC replicate
gDNA replicate

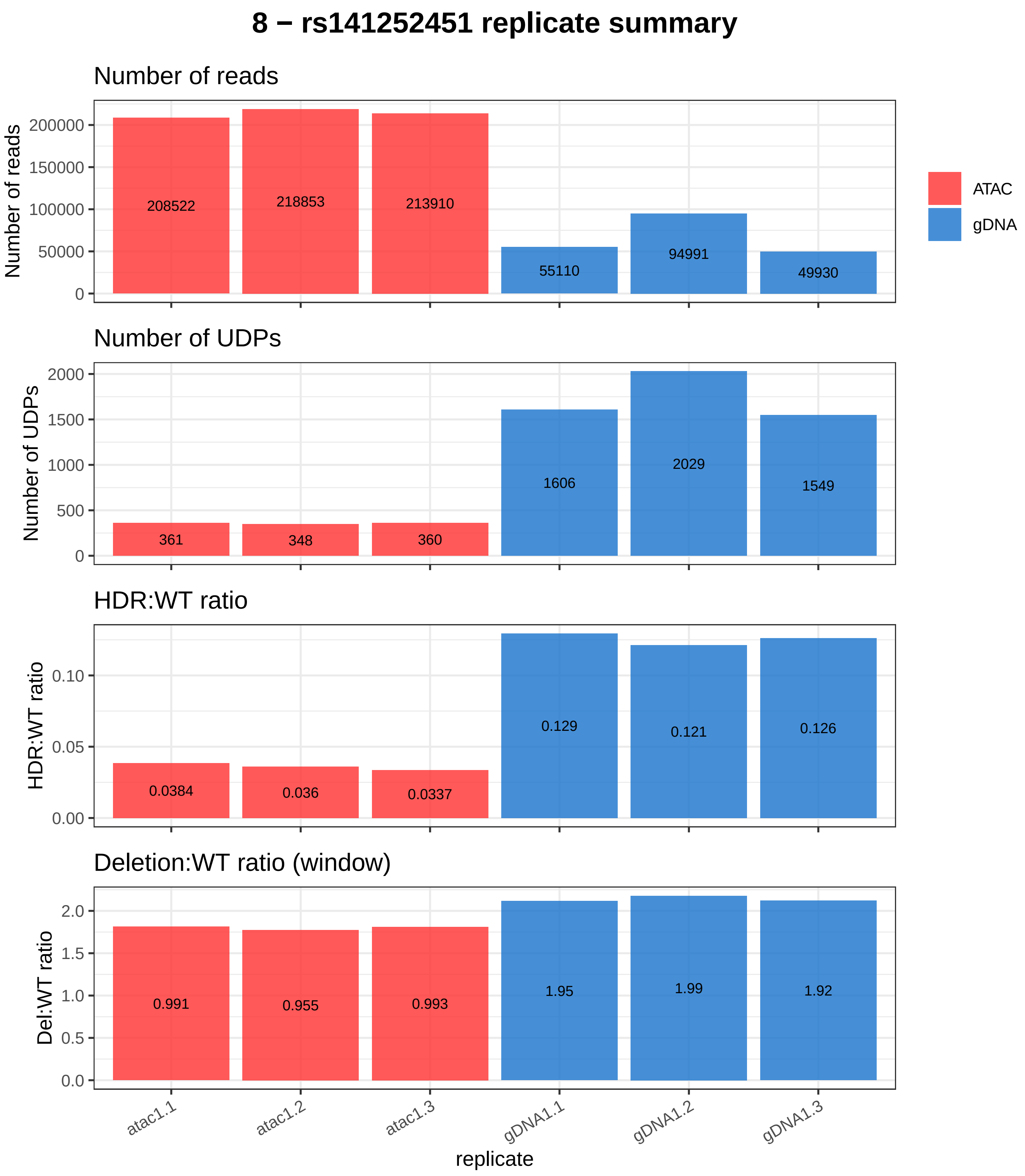
8 − rs141252451 replicate summary
Number of reads
Number of reads
208522
218853
213910
55110
94991
49930
ATAC
gDNA
Number of UDPs
Number of UDPs
361
348
360
1606
2029
1549
HDR:WT ratio
HDR:WT ratio
0.0384
0.036
0.0337
0.129
0.121
0.126
Deletion:WT ratio (window)
Del:WT ratio
0.991
0.955
0.993
1.95
1.99
1.92
atac1.1
atac1.2
atac1.3
gDNA1.1
gDNA1.2
gDNA1.3
replicate

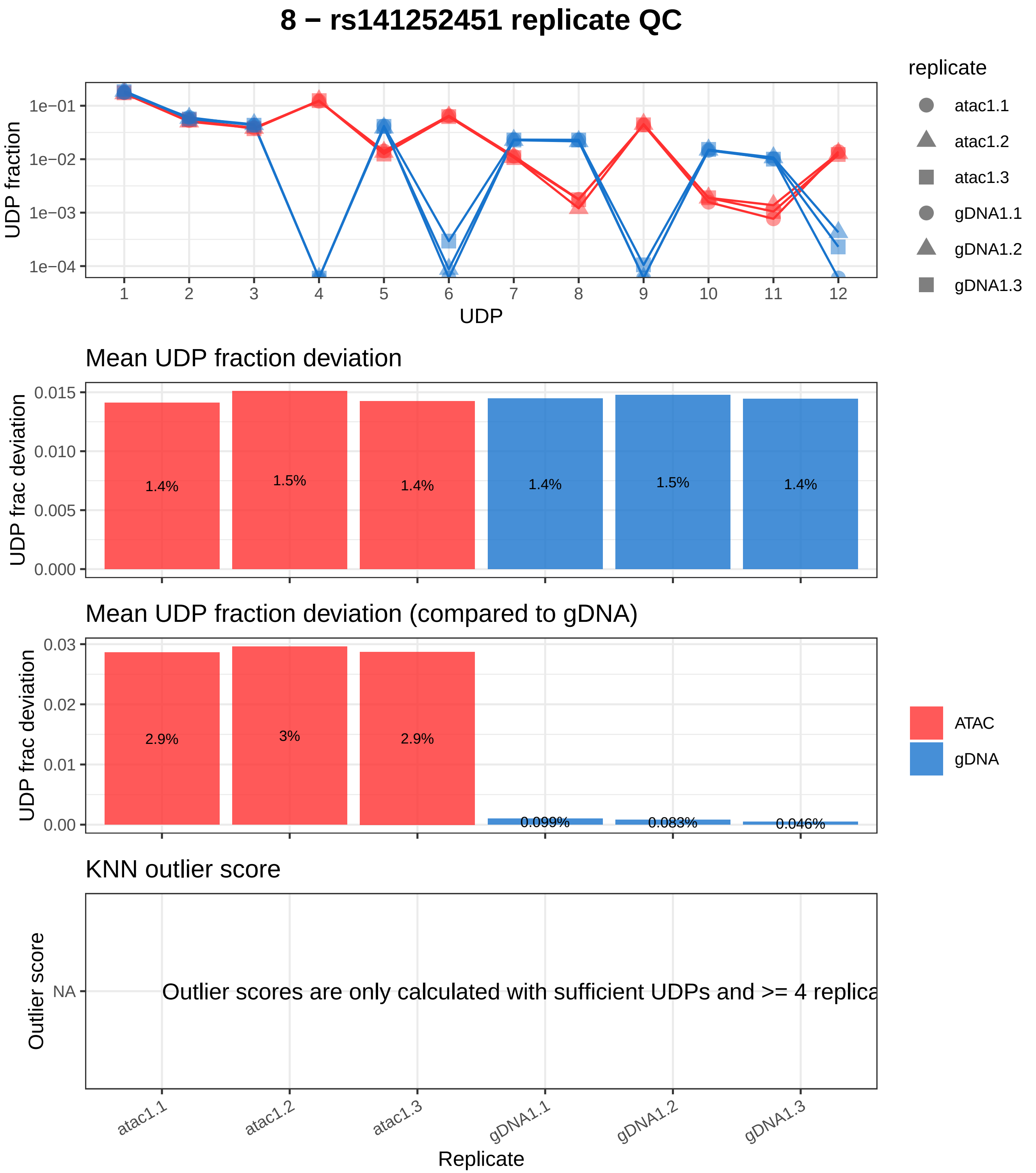
8 − rs141252451 replicate QC
UDP fraction
UDP
replicate
atac1.1
atac1.2
atac1.3
gDNA1.1
gDNA1.2
gDNA1.3
Mean UDP fraction deviation
UDP frac deviation
1.4%
1.5%
1.4%
1.4%
1.5%
1.4%
Mean UDP fraction deviation (compared to gDNA)
2.9%
3%
2.9%
0.099%
0.083%
0.046%
ATAC
gDNA
KNN outlier score
Outlier score
NA
Outlier scores are only calculated with sufficient UDPs and >= 4 replica
Replicate

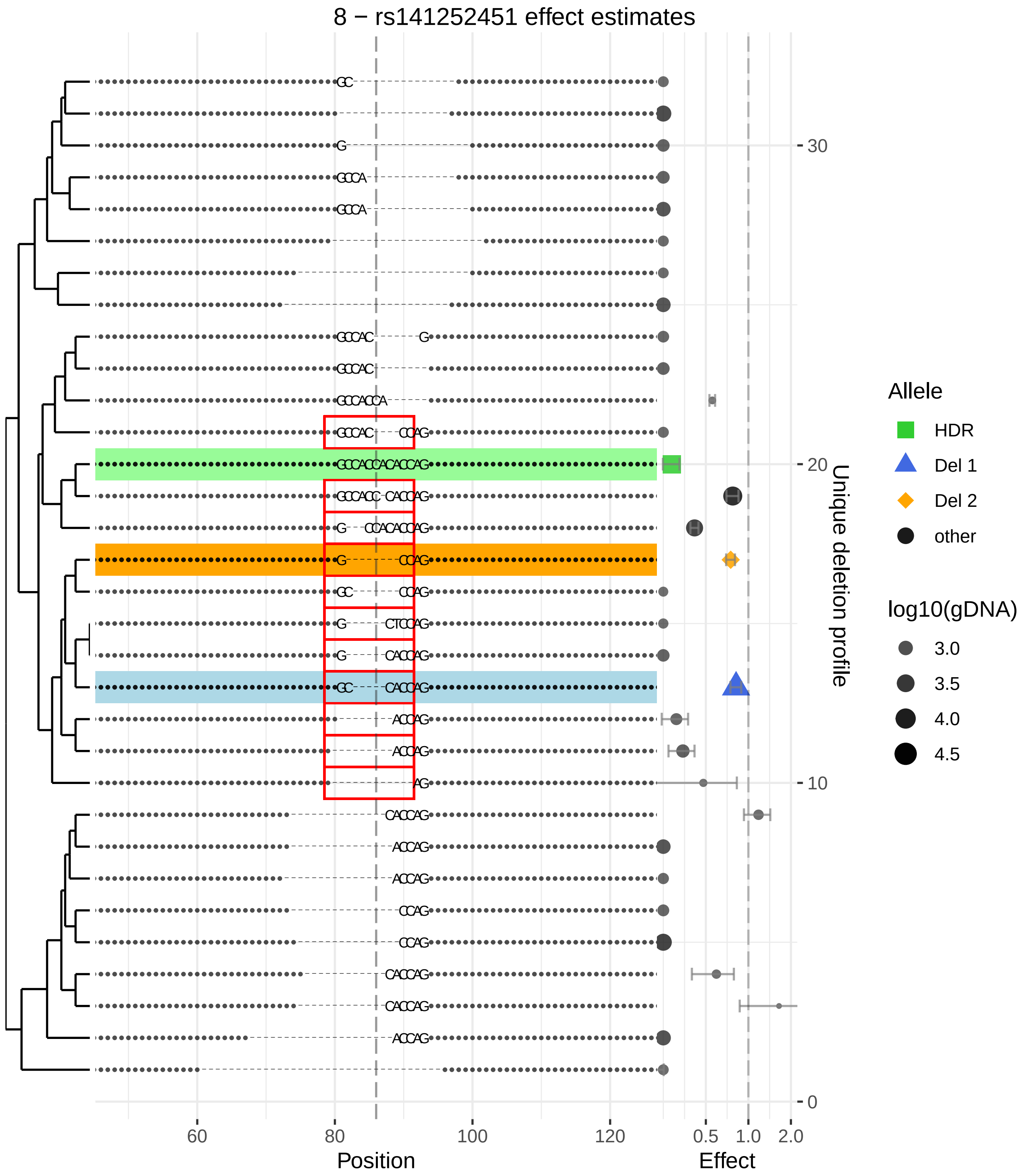

8 − rs141252451 effect estimates
Position
Effect
Unique deletion profile
Allele
HDR
Del 1
Del 2
other
log10(gDNA)
3.0
3.5
4.0
4.5
60
80
100
120
0.5
1.0
2.0
0
10
20
30

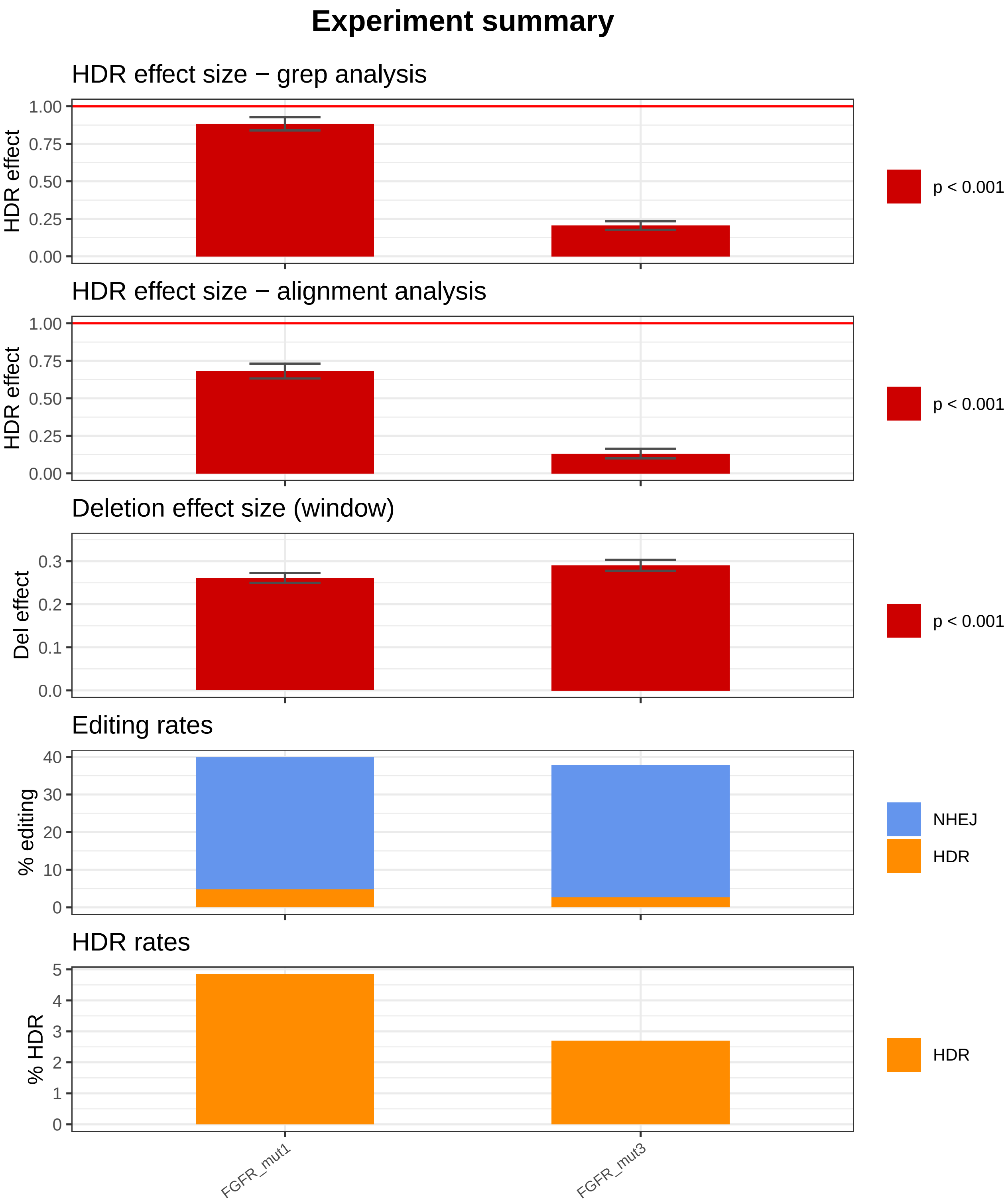
Experiment summary
HDR effect size − grep analysis
HDR effect
1.00
0.75
0.50
0.25
0.00
p < 0.001
HDR effect size − alignment analysis
HDR effect
1.00
0.75
0.50
0.25
0.00
p < 0.001
Deletion effect size (window)
Del effect
0.3
0.2
0.1
0.0
p < 0.001
Editing rates
% editing
40
30
20
10
0
NHEJ
HDR
HDR rates
% HDR
5
4
3
2
1
0
HDR
FGFR_mut1
FGFR_mut3

FGFR_mut1 grep summary

Mean HDR frac gDNA: 5%, ATAC: 0.078%
Mean WT frac gDNA: 53%, ATAC: 0.94%

ATAC:gDNA ratio (HDR/WT): 0.884
95% CI: (0.839, 0.928),   p = 0.000121

[illegible]	gDNA2_3_1	gDNA2_1_2	gDNA2_2_2	gDNA2_3_2	gDNA2_1_3	gDNA2_2_3	gDNA2_3_3	ATAC2_1_1	ATAC2_2_1	ATAC2_3_1	ATAC2_1_2	ATAC2_2_2	ATAC2_3_2	[illegible]
	gDNA	gDNA	gDNA	gDNA	gDNA	gDNA	gDNA	ATAC	ATAC	ATAC	ATAC	ATAC	ATAC	
	129563	88421	71282	92935	92062	118785	141527	385523	406512	399759	463317	445638	376497	[illegible]
	6331	4367	3568	4376	4658	5959	7038	276	325	318	310	385	318	
	67873	47143	38307	49660	47863	62930	73565	3552	3694	4041	3790	4522	3556	
	0.0933	0.0926	0.0931	0.0881	0.0973	0.0947	0.0957	0.0777	0.088	0.0787	0.0818	0.0851	0.0894	[illegible]
	4.89%	4.94%	5.01%	4.71%	5.06%	5.02%	4.97%	0.07%	0.08%	0.08%	0.07%	0.09%	0.08%	
	52.39%	53.32%	53.74%	53.44%	51.99%	52.98%	51.98%	0.92%	0.91%	1.01%	0.82%	1.01%	0.94%	

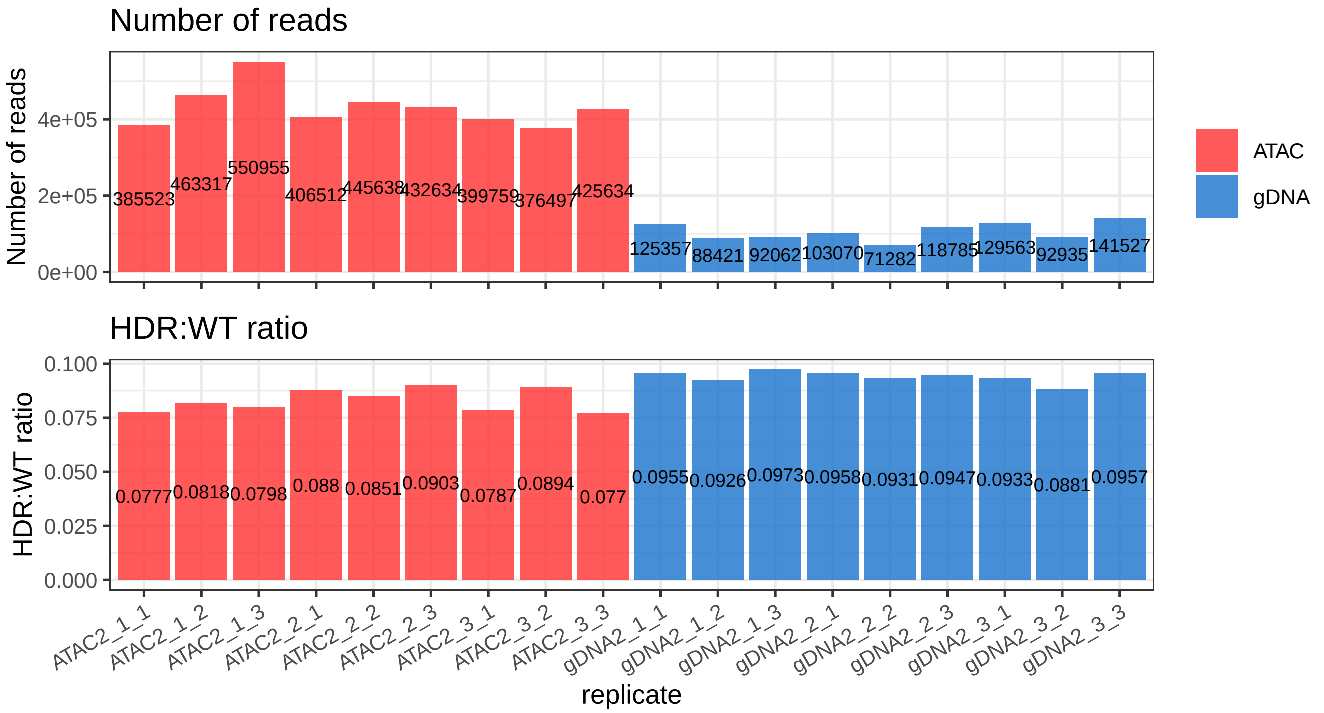

FGFR_mut1 analysis summary

Mean HDR frac gDNA: 4.8%, ATAC: 3%
Mean DEL frac gDNA: 35%, ATAC: 14%
Mean WT frac gDNA: 56%, ATAC: 51%

ATAC:gDNA ratio (HDR/WT): 0.682
95% CI: (0.632, 0.731),   p = 2.44e−08

ATAC:gDNA ratio (DEL/WT) [109−121]: 0.261
95% CI: (0.25, 0.273),   p = 4.86e−22
ATAC:gDNA ratio (DEL/WT) − Del 1: 0.17
95% CI: (0.101, 0.239),   p = 3.22e−12
ATAC:gDNA ratio (DEL/WT) − Del 2: 0.133
95% CI: (0.0951, 0.17),   p = 9.2e−17

DNA2_2_1	gDNA2_3_1	gDNA2_1_2	gDNA2_2_2	gDNA2_3_2	gDNA2_1_3	gDNA2_2_3	gDNA2_3_3	ATAC2_1_1	ATAC2_2_1	ATAC2_3_1	ATAC2_1_2	ATAC2_2_2	ATAC2
gDNA	gDNA	gDNA	gDNA	gDNA	gDNA	gDNA	gDNA	ATAC	ATAC	ATAC	ATAC	ATAC	ATA
1798	2123	1855	1540	1874	1913	1924	2186	315	341	361	329	353	35
0.0868	0.0863	0.0848	0.0851	0.0806	0.0893	0.0862	0.0889	0.0519	0.0617	0.0608	0.0624	0.0633	0.05
0.615	0.639	0.608	0.589	0.611	0.656	0.618	0.65	0.127	0.309	0.441	0.129	0.267	0.4
4.90%	4.81%	4.82%	4.88%	4.57%	4.91%	4.86%	4.92%	2.79%	3.20%	2.85%	3.19%	3.33%	2.52
34.72%	35.64%	34.54%	33.76%	34.67%	36.12%	34.84%	35.92%	6.84%	16.01%	20.66%	6.59%	14.01%	20.2
39.62%	40.45%	39.36%	38.64%	39.24%	41.03%	39.69%	40.84%	9.63%	19.21%	23.51%	9.78%	17.33%	22.7
56.43%	55.74%	56.82%	57.32%	56.77%	55.04%	56.36%	55.29%	53.79%	51.87%	46.89%	51.15%	52.53%	45.8
103070	129563	88421	71282	92935	92062	118785	141527	385523	406512	399759	463317	445638	3764
4291	5297	3591	2926	3612	3879	4934	5957	114	136	149	141	175	11
49420	61359	42370	34369	44820	43453	57246	66990	2195	2206	2449	2259	2764	20
30408	39233	25756	20246	27372	28513	35385	43523	279	681	1079	291	737	92
15213	18593	13382	11060	13583	12808	16656	19227	86	63	87	81	82	5
0	0	0	0	0	0	1	0	2499	2937	3589	2768	3326	310
62	87	71	64	81	68	104	116	924	942	1000	931	1155	105
220	796	400	194	318	237	446	1033	377933	398317	389860	455121	435813	3677
0	0	0	0	0	0	0	0	0	0	0	0	0	0

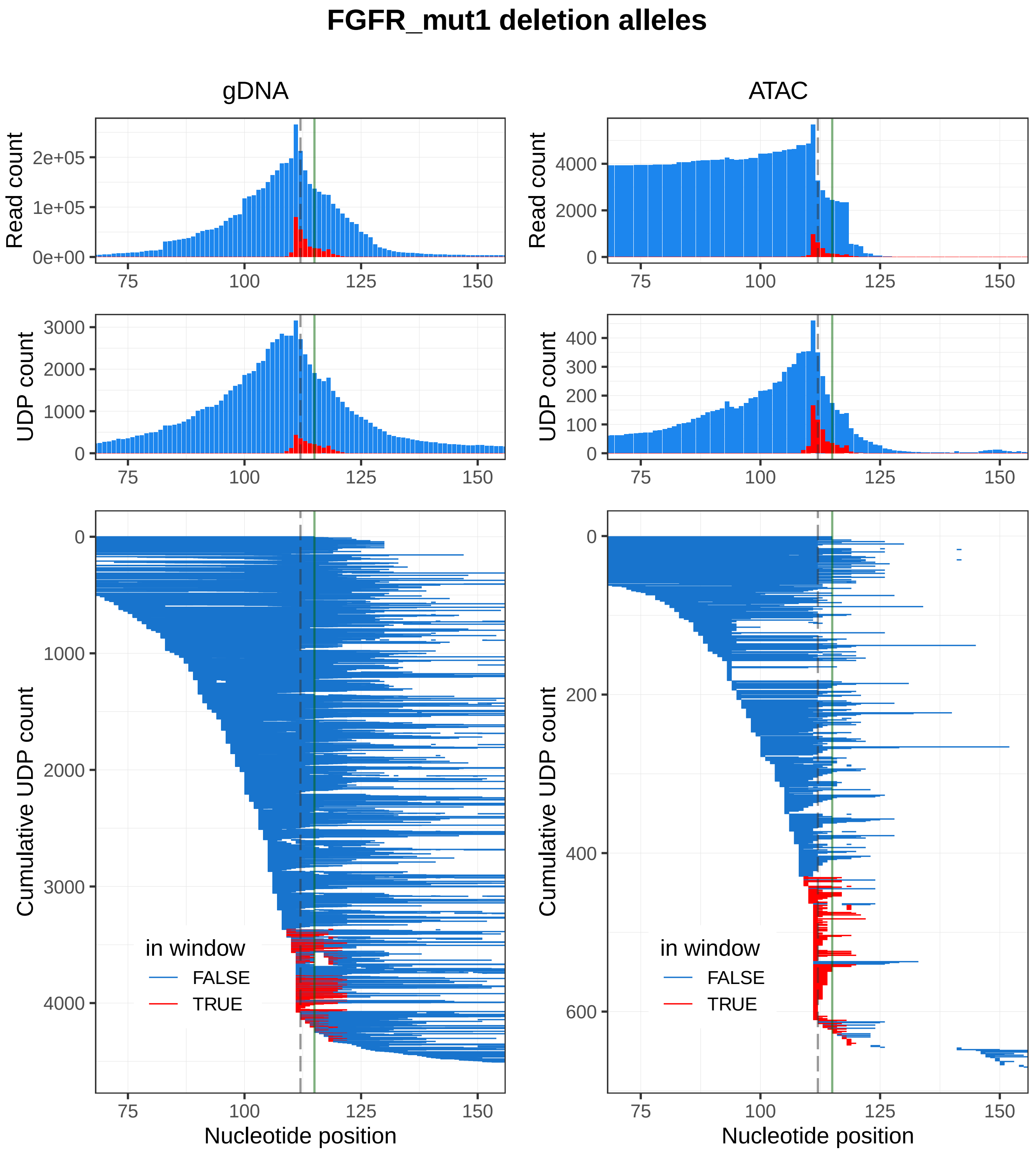

FGFR_mut1 deletion alleles
gDNA
ATAC
Read count
UDP count
Cumulative UDP count
Nucleotide position
in window
FALSE
TRUE
0e+00
1e+05
2e+05
0
2000
4000
1000
3000
100
200
300
400
600
75
100
125
150

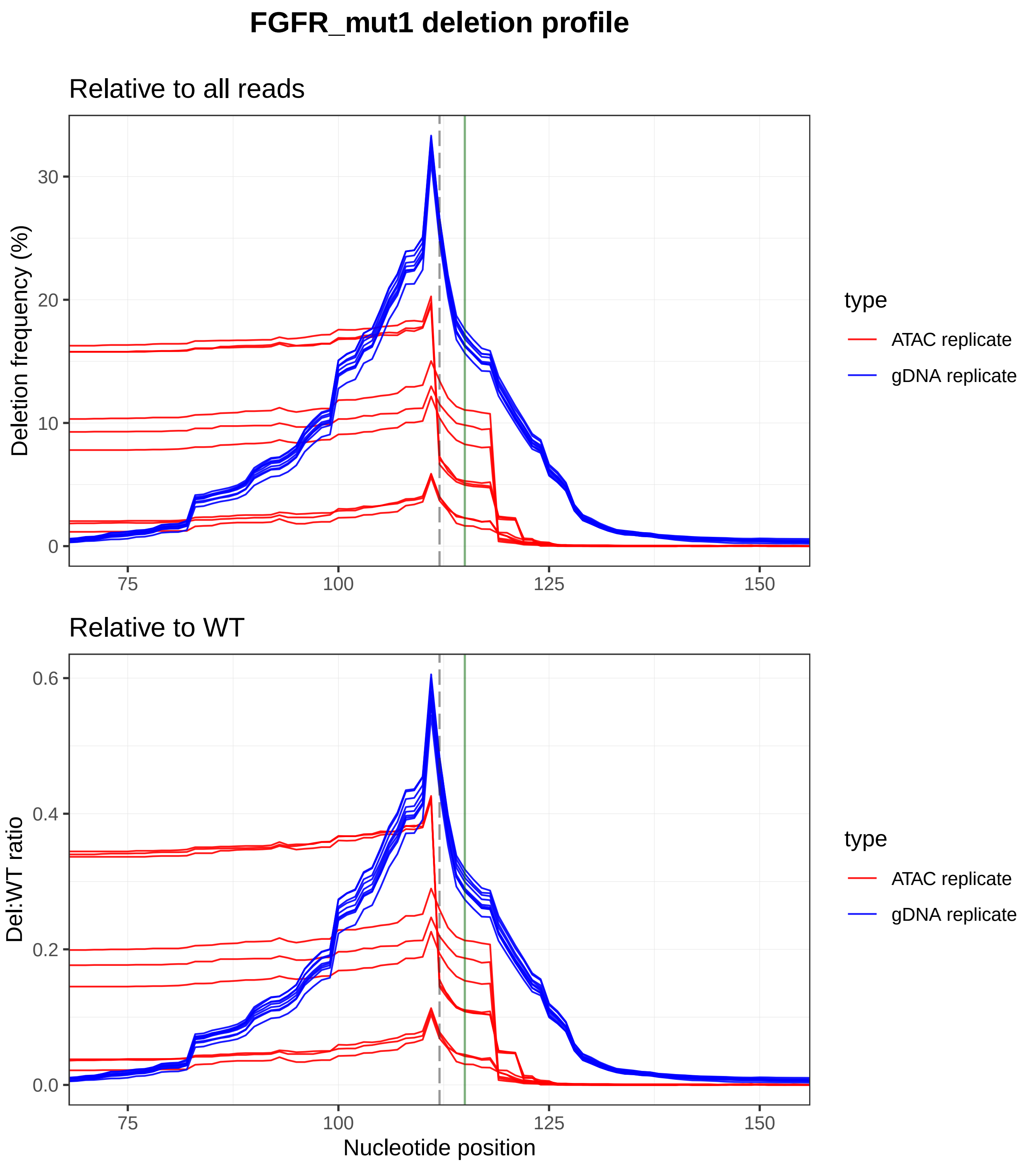
FGFR_mut1 deletion profile
Relative to all reads
Deletion frequency (%)
0
10
20
30
75
100
125
150
type
ATAC replicate
gDNA replicate
Relative to WT
Del:WT ratio
0.0
0.2
0.4
0.6
75
100
125
150
Nucleotide position
type
ATAC replicate
gDNA replicate

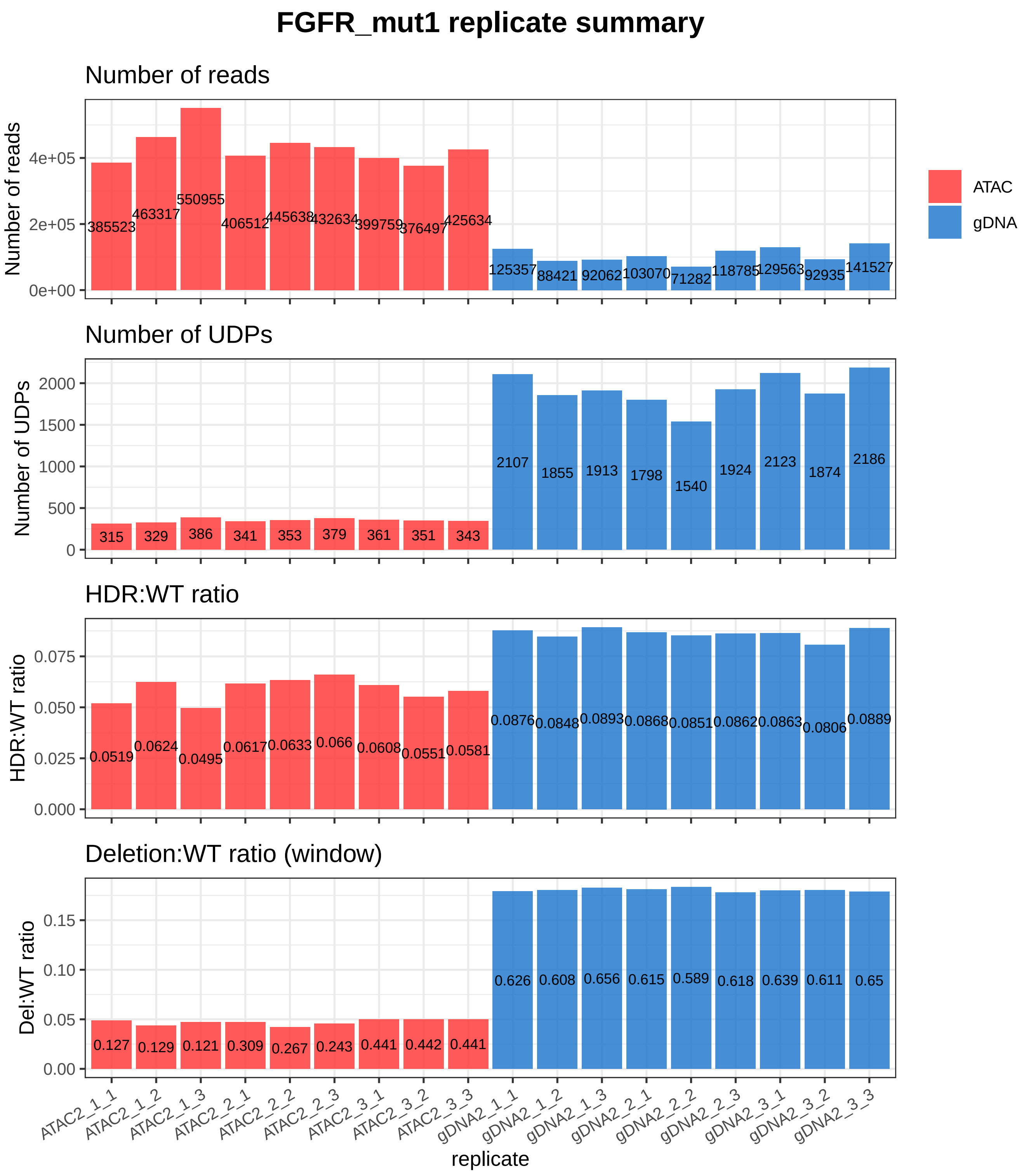
FGFR_mut1 replicate summary
Number of reads
385523
463317
550955
406512
445638
432634
399759
376497
425634
125357
88421
92062
103070
71282
118785
129563
92935
141527
Number of UDPs
315
329
386
341
353
379
361
351
343
2107
1855
1913
1798
1540
1924
2123
1874
2186
HDR:WT ratio
0.0519
0.0624
0.0495
0.0617
0.0633
0.066
0.0608
0.0551
0.0581
0.0876
0.0848
0.0893
0.0868
0.0851
0.0862
0.0863
0.0806
0.0889
Deletion:WT ratio (window)
0.127
0.129
0.121
0.309
0.267
0.243
0.441
0.442
0.441
0.626
0.608
0.656
0.615
0.589
0.618
0.639
0.611
0.65
Del:WT ratio
replicate
ATAC2_1_1
ATAC2_1_2
ATAC2_1_3
ATAC2_2_1
ATAC2_2_2
ATAC2_2_3
ATAC2_3_1
ATAC2_3_2
ATAC2_3_3
gDNA2_1_1
gDNA2_1_2
gDNA2_1_3
gDNA2_2_1
gDNA2_2_2
gDNA2_2_3
gDNA2_3_1
gDNA2_3_2
gDNA2_3_3
ATAC
gDNA

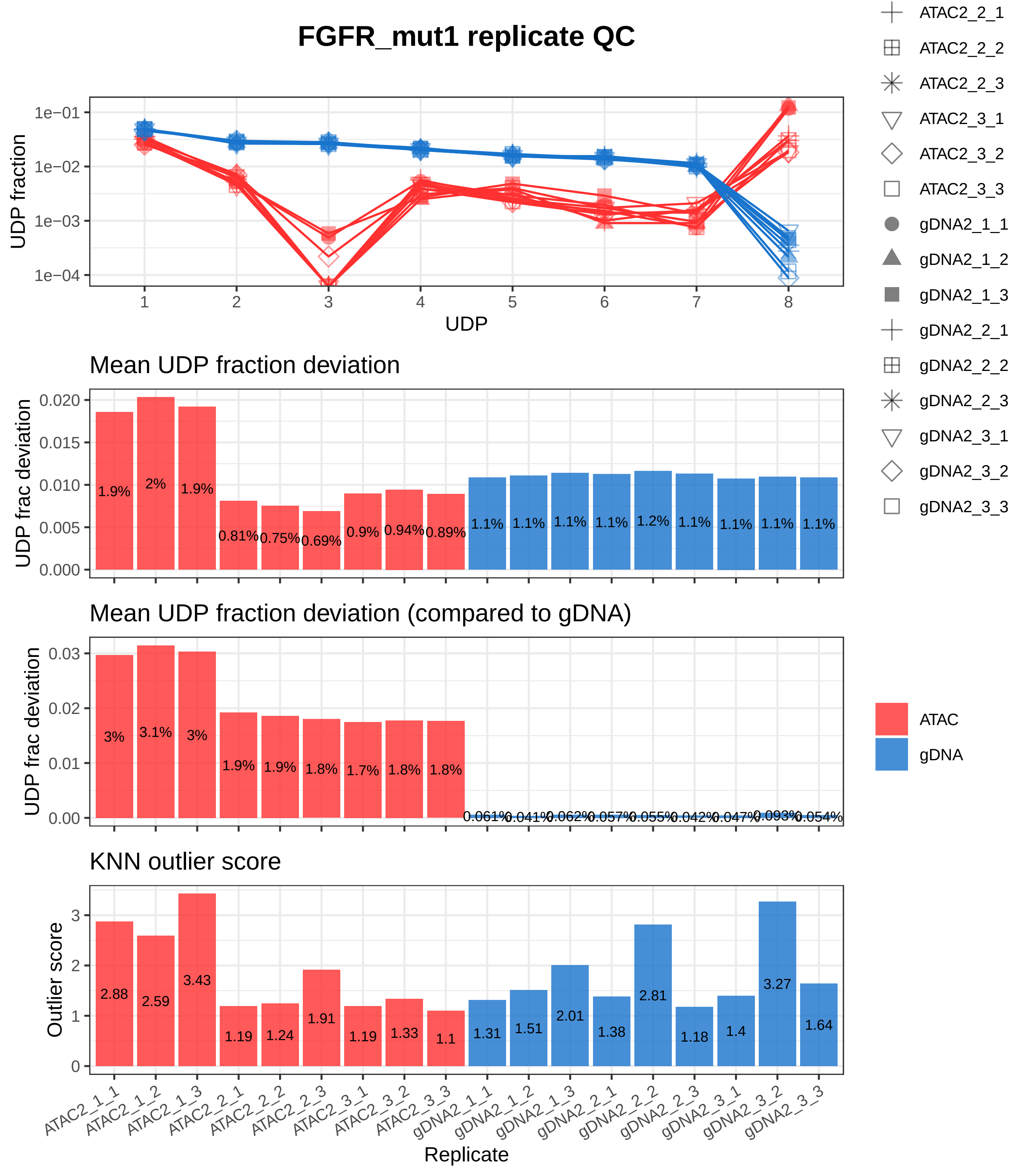
FGFR_mut1 replicate QC
UDP fraction
1e-01
1e-02
1e-03
1e-04
UDP
1 2 3 4 5 6 7 8
ATAC2_2_1
ATAC2_2_2
ATAC2_2_3
ATAC2_3_1
ATAC2_3_2
ATAC2_3_3
gDNA2_1_1
gDNA2_1_2
gDNA2_1_3
gDNA2_2_1
gDNA2_2_2
gDNA2_2_3
gDNA2_3_1
gDNA2_3_2
gDNA2_3_3
Mean UDP fraction deviation
UDP frac deviation
0.000 0.005 0.010 0.015 0.020
1.9% 2% 1.9% 0.81% 0.75% 0.69% 0.9% 0.94% 0.89% 1.1% 1.1% 1.1% 1.1% 1.2% 1.1% 1.1% 1.1% 1.1%
Mean UDP fraction deviation (compared to gDNA)
UDP frac deviation
0.00 0.01 0.02 0.03
3% 3.1% 3% 1.9% 1.9% 1.8% 1.7% 1.8% 1.8% 0.061% 0.041% 0.062% 0.057% 0.055% 0.042% 0.047% 0.093% 0.054%
ATAC
gDNA
KNN outlier score
Outlier score
0 1 2 3
2.88 2.59 3.43 1.19 1.24 1.91 1.19 1.33 1.1 1.31 1.51 2.01 1.38 2.81 1.18 1.4 3.27 1.64
ATAC2_1_1 ATAC2_1_2 ATAC2_1_3 ATAC2_2_1 ATAC2_2_2 ATAC2_2_3 ATAC2_3_1 ATAC2_3_2 ATAC2_3_3 gDNA2_1_1 gDNA2_1_2 gDNA2_1_3 gDNA2_2_1 gDNA2_2_2 gDNA2_2_3 gDNA2_3_1 gDNA2_3_2 gDNA2_3_3
Replicate

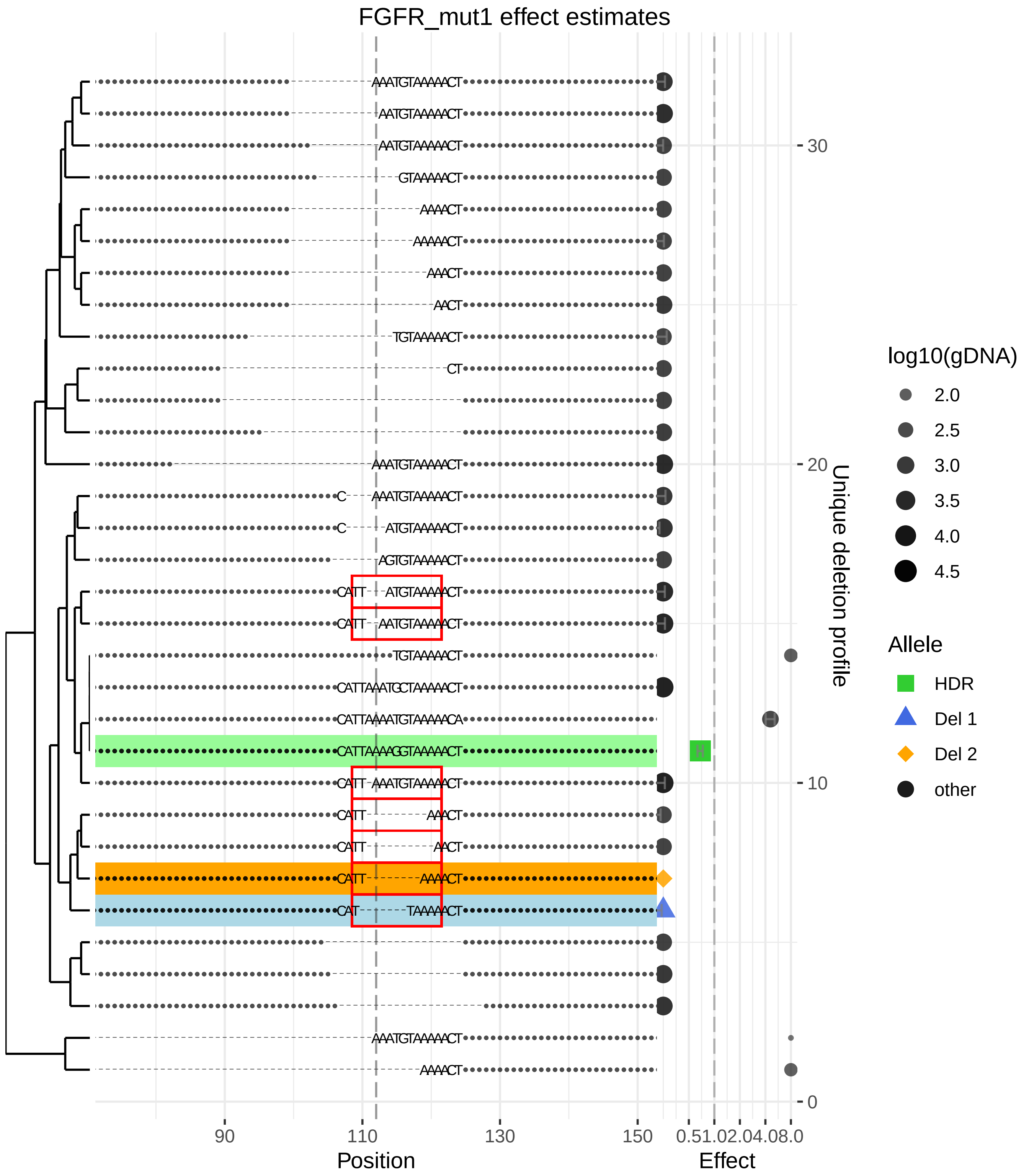
FGFR_mut1 effect estimates
AAATGTAAAAACT
AATGTAAAAACT
GTAAAAACT
AAAACT
AAAAACT
AAACT
AACT
TGTAAAAACT
CT
C
ATGTAAAAACT
AGTGTAAAAACT
CATT
CATTAAATGCTAAAAACT
CATTAAAATGTAAAAACA
CATTAAAAGGTAAAAACT
CAT
TAAAACT
90
110
130
150
Position
0.5 1.0 2.0 4.0 8.0
Effect
30
20
10
0
Unique deletion profile
log10(gDNA)
2.0
2.5
3.0
3.5
4.0
4.5
Allele
HDR
Del 1
Del 2
other

FGFR_mut3 grep summary

Mean HDR frac gDNA: 2.8%, ATAC: 0.0082%
Mean WT frac gDNA: 53%, ATAC: 0.75%

ATAC:gDNA ratio (HDR/WT): 0.206
95% CI: (0.177, 0.234), p = 3.29e−17

1	gDNA2_3_1	gDNA2_1_2	gDNA2_2_2	gDNA2_3_2	gDNA2_1_3	gDNA2_2_3	gDNA2_3_3	ATAC2_1_1	ATAC2_2_1	ATAC2_3_1	ATAC2_1_2	ATAC2_2_2	ATAC2_3_2	ATA
	gDNA	gDNA	gDNA	gDNA	gDNA	gDNA	gDNA	ATAC	ATAC	ATAC	ATAC	ATAC	ATAC	
	129563	88421	71282	92935	92062	118785	141527	385523	406512	399759	463317	445638	376497	5
	3492	2508	2107	2722	2608	3367	3821	38	27	24	36	43	38	
	67711	47037	38237	49564	47759	62813	73393	2905	2968	3232	3019	3641	2869	
	0.0516	0.0533	0.0551	0.0549	0.0546	0.0536	0.0521	0.0131	0.0091	0.00743	0.0119	0.0118	0.0132	0
	2.70%	2.84%	2.96%	2.93%	2.83%	2.83%	2.70%	0.01%	0.01%	0.01%	0.01%	0.01%	0.01%	
	52.26%	53.20%	53.64%	53.33%	51.88%	52.88%	51.86%	0.75%	0.73%	0.81%	0.65%	0.82%	0.76%	

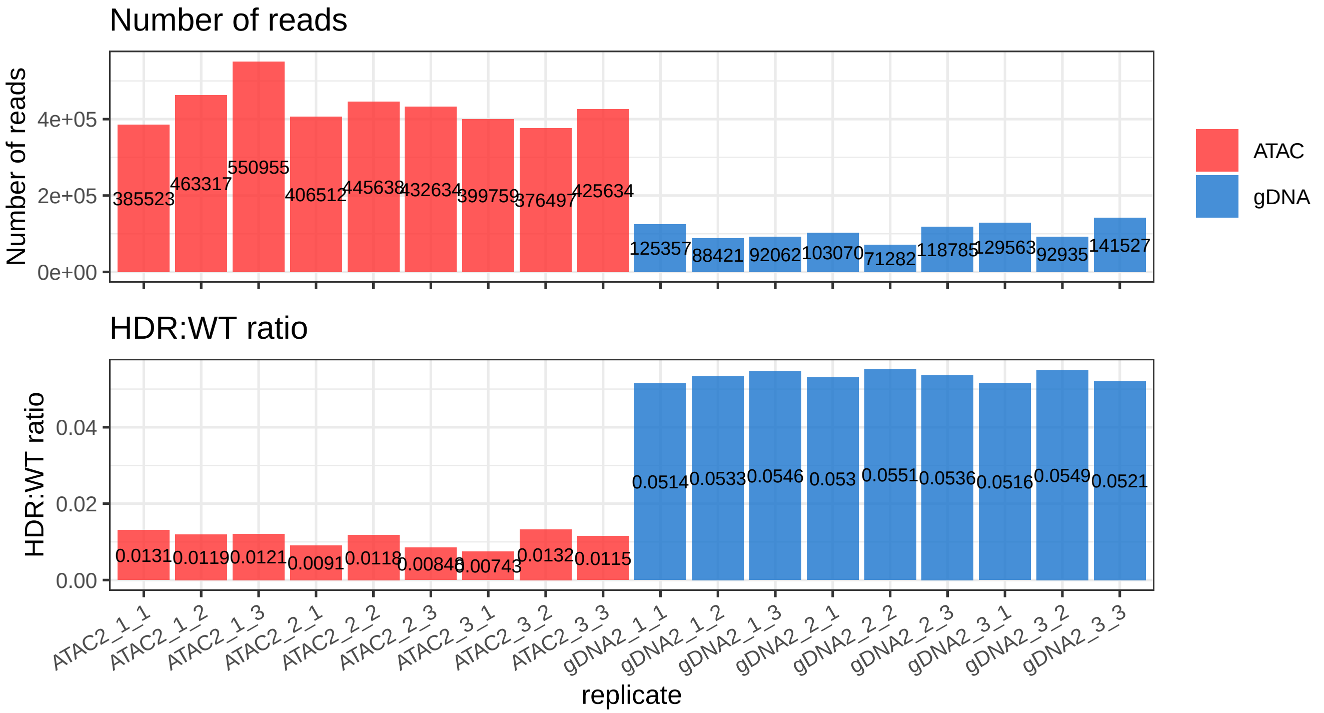

FGFR_mut3 analysis summary

Mean HDR frac gDNA: 2.7%, ATAC: 0.29%
Mean DEL frac gDNA: 35%, ATAC: 14%
Mean WT frac gDNA: 56%, ATAC: 46%

ATAC:gDNA ratio (HDR/WT): 0.132
95% CI: (0.0999, 0.164),   p = 9.89e−16

ATAC:gDNA ratio (DEL/WT) [109−121]: 0.291
95% CI: (0.278, 0.303),   p = 2.17e−20
ATAC:gDNA ratio (DEL/WT) − Del 1: 0.185
95% CI: (0.103, 0.267),   p = 1.17e−10
ATAC:gDNA ratio (DEL/WT) − Del 2: 0.148
95% CI: (0.106, 0.19),   p = 1.07e−15

DNA2_2_1	gDNA2_3_1	gDNA2_1_2	gDNA2_2_2	gDNA2_3_2	gDNA2_1_3	gDNA2_2_3	gDNA2_3_3	ATAC2_1_1	ATAC2_2_1	ATAC2_3_1	ATAC2_1_2	ATAC2_2_2	ATAC2
gDNA	gDNA	gDNA	gDNA	gDNA	gDNA	gDNA	gDNA	ATAC	ATAC	ATAC	ATAC	ATAC	ATA
1866	2217	1926	1593	1942	1966	2004	2271	370	401	414	373	417	39
0.0484	0.0467	0.0478	0.0491	0.0495	0.0487	0.0482	0.0468	0.00564	0.00453	0.00362	0.00887	0.00404	0.008
0.616	0.641	0.609	0.59	0.612	0.657	0.619	0.651	0.143	0.343	0.489	0.143	0.298	0.48
2.73%	2.60%	2.71%	2.81%	2.80%	2.68%	2.71%	2.58%	0.27%	0.21%	0.15%	0.41%	0.19%	0.37
34.72%	35.64%	34.54%	33.76%	34.67%	36.11%	34.83%	35.92%	6.85%	16.04%	20.71%	6.60%	14.03%	20.2
37.45%	38.23%	37.25%	36.57%	37.47%	38.79%	37.55%	38.50%	7.12%	16.25%	20.86%	7.00%	14.22%	20.6
56.33%	55.64%	56.74%	57.22%	56.68%	54.95%	56.27%	55.20%	47.89%	46.77%	42.37%	45.99%	47.10%	41.6
103070	129563	88421	71282	92935	92062	118785	141527	385523	406512	399759	463317	445638	3764
2389	2858	2024	1686	2214	2114	2758	3130	11	9	8	18	10	1
49335	61249	42310	34313	44749	43389	57161	66877	1950	1986	2208	2029	2474	189
30408	39233	25756	20246	27372	28513	35385	43523	279	681	1079	291	737	92
15213	18593	13382	11060	13583	12808	16656	19227	86	63	87	81	82	5
0	0	0	0	0	0	1	0	2499	2937	3589	2768	3326	310
62	84	65	58	78	63	96	105	933	949	1012	935	1164	105
220	796	400	194	318	237	446	1033	377933	398317	389860	455121	435813	3677
0	0	0	0	0	0	0	0	0	0	0	0	0	0

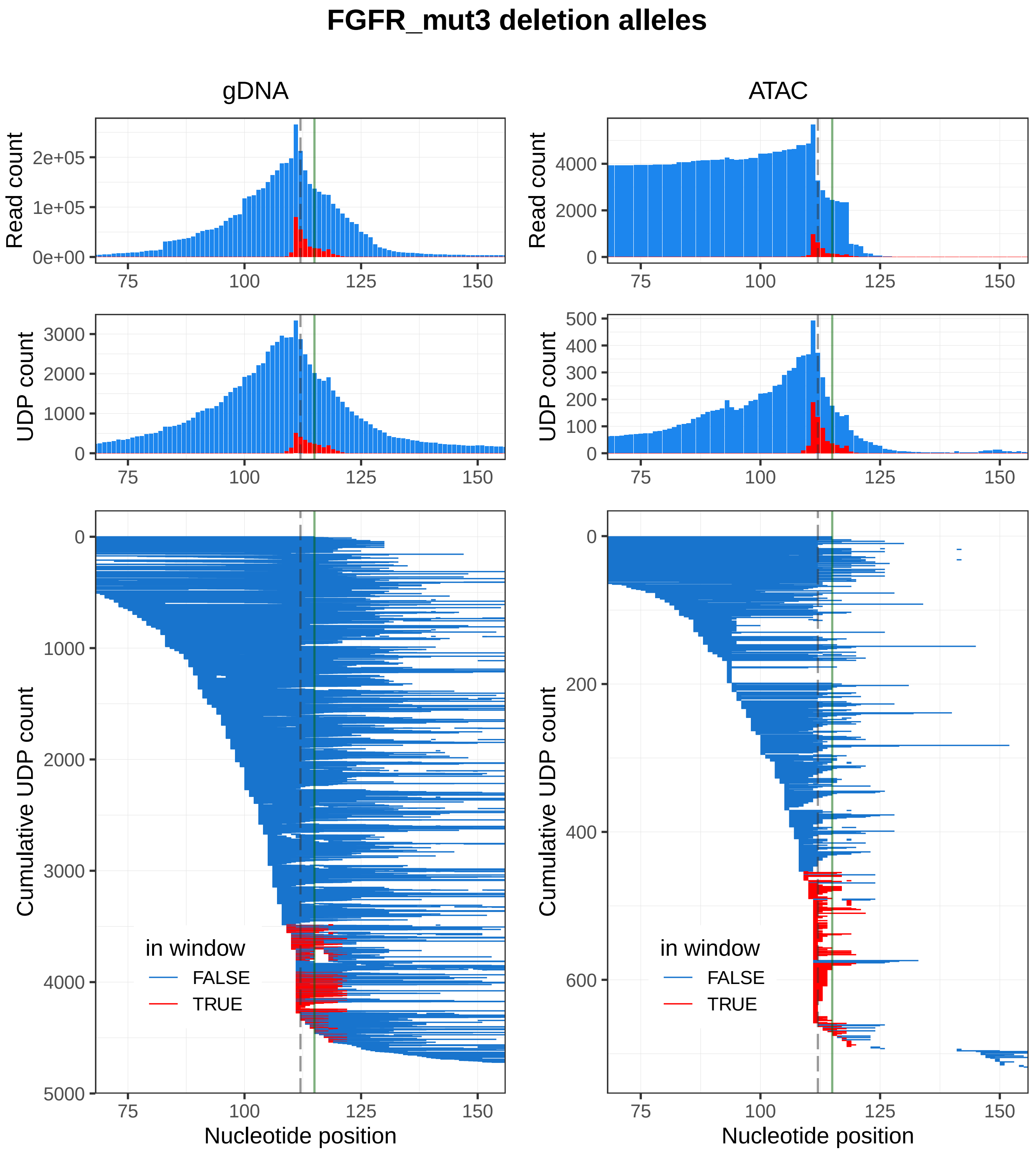
FGFR_mut3 deletion alleles
gDNA
ATAC
Read count
UDP count
Cumulative UDP count
Nucleotide position
in window
FALSE
TRUE
2e+05
1e+05
0e+00
4000
2000
0
3000
2000
1000
500
400
300
200
100
5000
600
75
100
125
150

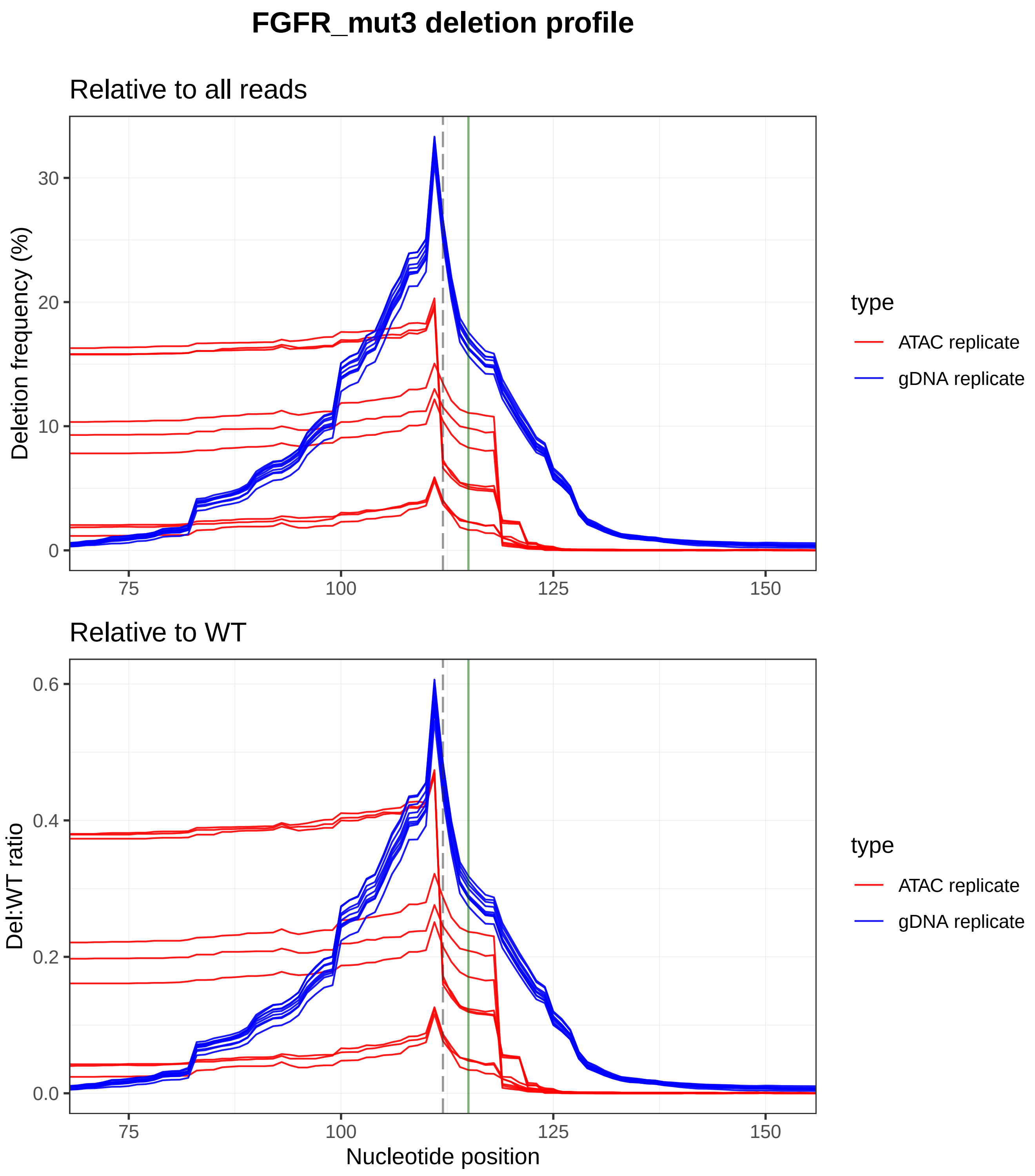
FGFR_mut3 deletion profile
Relative to all reads
Deletion frequency (%)
0
10
20
30
75
100
125
150
type
ATAC replicate
gDNA replicate
Relative to WT
Del:WT ratio
0.0
0.2
0.4
0.6
75
100
125
150
Nucleotide position
type
ATAC replicate
gDNA replicate

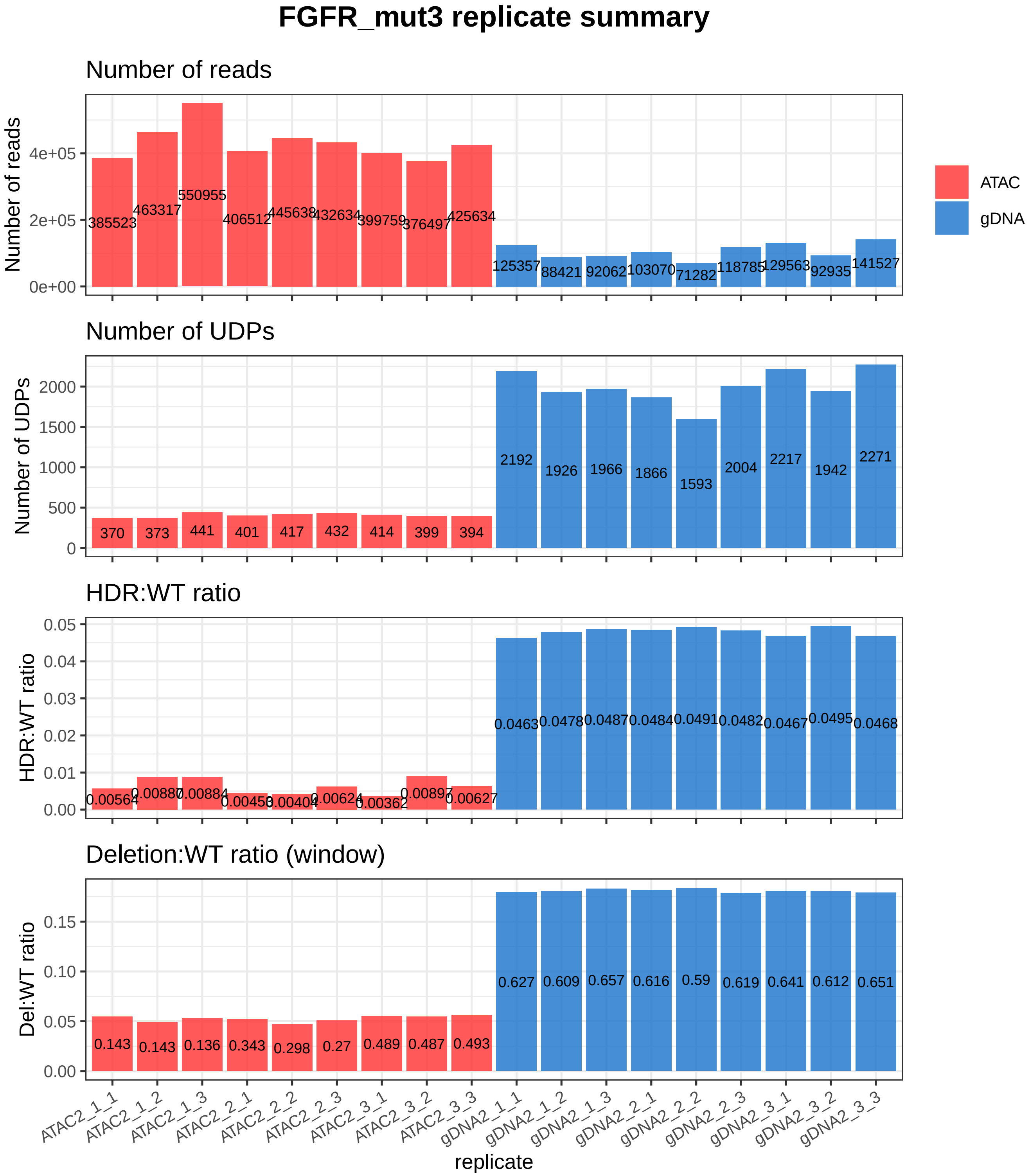
FGFR_mut3 replicate summary
Number of reads
Number of reads
4e+05
2e+05
0e+00
385523
463317
550955
406512
445638
432634
399759
376497
425634
125357
88421
92062
103070
71282
118785
129563
92935
141527
Number of UDPs
Number of UDPs
2000
1500
1000
500
0
370
373
441
401
417
432
414
399
394
2192
1926
1966
1866
1593
2004
2217
1942
2271
HDR:WT ratio
HDR:WT ratio
0.05
0.04
0.03
0.02
0.01
0.00
0.00564
0.00887
0.00884
0.00453
0.00404
0.00624
0.00362
0.00897
0.00627
0.0463
0.0478
0.0487
0.0484
0.0491
0.0482
0.0467
0.0495
0.0468
Deletion:WT ratio (window)
Del:WT ratio
0.15
0.10
0.05
0.00
0.143
0.143
0.136
0.343
0.298
0.27
0.489
0.487
0.493
0.627
0.609
0.657
0.616
0.59
0.619
0.641
0.612
0.651
ATAC2_1_1
ATAC2_1_2
ATAC2_1_3
ATAC2_2_1
ATAC2_2_2
ATAC2_2_3
ATAC2_3_1
ATAC2_3_2
ATAC2_3_3
gDNA2_1_1
gDNA2_1_2
gDNA2_1_3
gDNA2_2_1
gDNA2_2_2
gDNA2_2_3
gDNA2_3_1
gDNA2_3_2
gDNA2_3_3
replicate
ATAC
gDNA

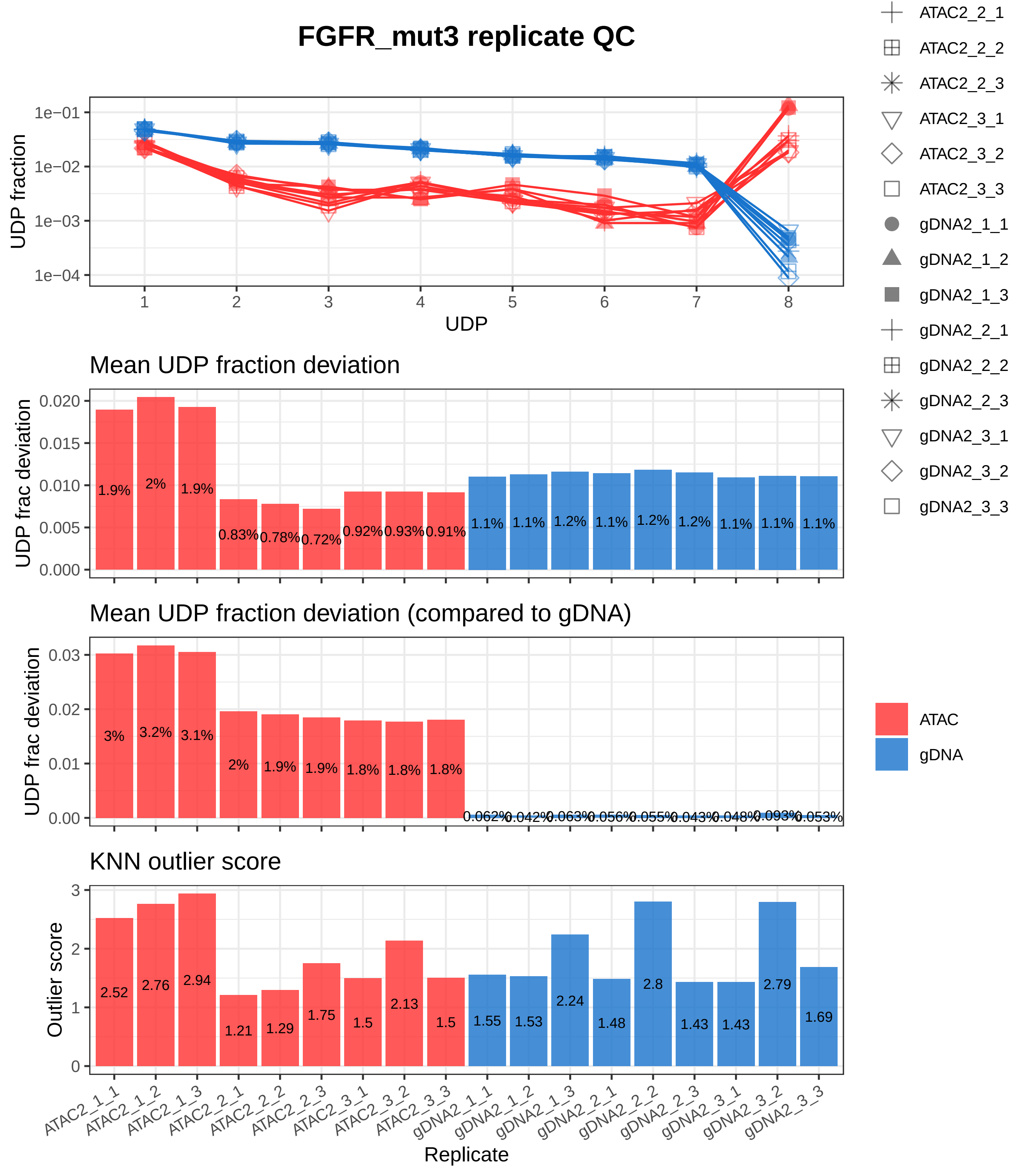
FGFR_mut3 replicate QC
UDP fraction
1e-01
1e-02
1e-03
1e-04
UDP
1 2 3 4 5 6 7 8
Mean UDP fraction deviation
UDP frac deviation
1.9% 2% 1.9% 0.83% 0.78% 0.72% 0.92% 0.93% 0.91% 1.1% 1.1% 1.2% 1.1% 1.2% 1.2% 1.1% 1.1% 1.1%
Mean UDP fraction deviation (compared to gDNA)
3% 3.2% 3.1% 2% 1.9% 1.9% 1.8% 1.8% 1.8% 0.062% 0.042% 0.063% 0.056% 0.055% 0.043% 0.048% 0.093% 0.053%
KNN outlier score
Outlier score
2.52 2.76 2.94 1.21 1.29 1.75 1.5 2.13 1.5 1.55 1.53 2.24 1.48 2.8 1.43 1.43 2.79 1.69
Replicate
ATAC2_1_1 ATAC2_1_2 ATAC2_1_3 ATAC2_2_1 ATAC2_2_2 ATAC2_2_3 ATAC2_3_1 ATAC2_3_2 ATAC2_3_3 gDNA2_1_1 gDNA2_1_2 gDNA2_1_3 gDNA2_2_1 gDNA2_2_2 gDNA2_2_3 gDNA2_3_1 gDNA2_3_2 gDNA2_3_3
ATAC
gDNA

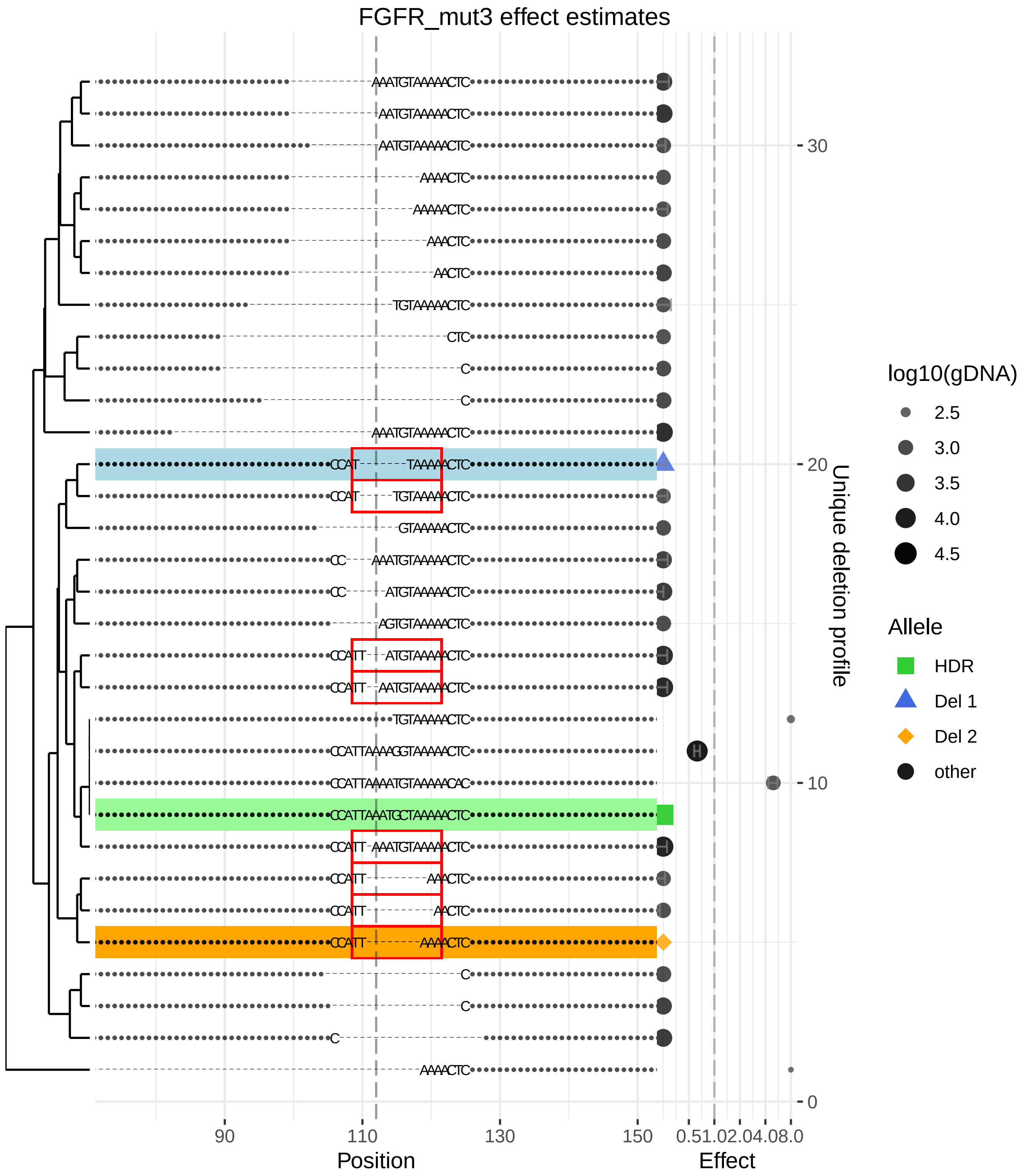

FGFR_mut3 effect estimates
Position
Effect
Unique deletion profile
log10(gDNA)
2.5
3.0
3.5
4.0
4.5
Allele
HDR
Del 1
Del 2
other
90
110
130
150
0.5
1.0
2.0
4.0
8.0
0
10
20
30

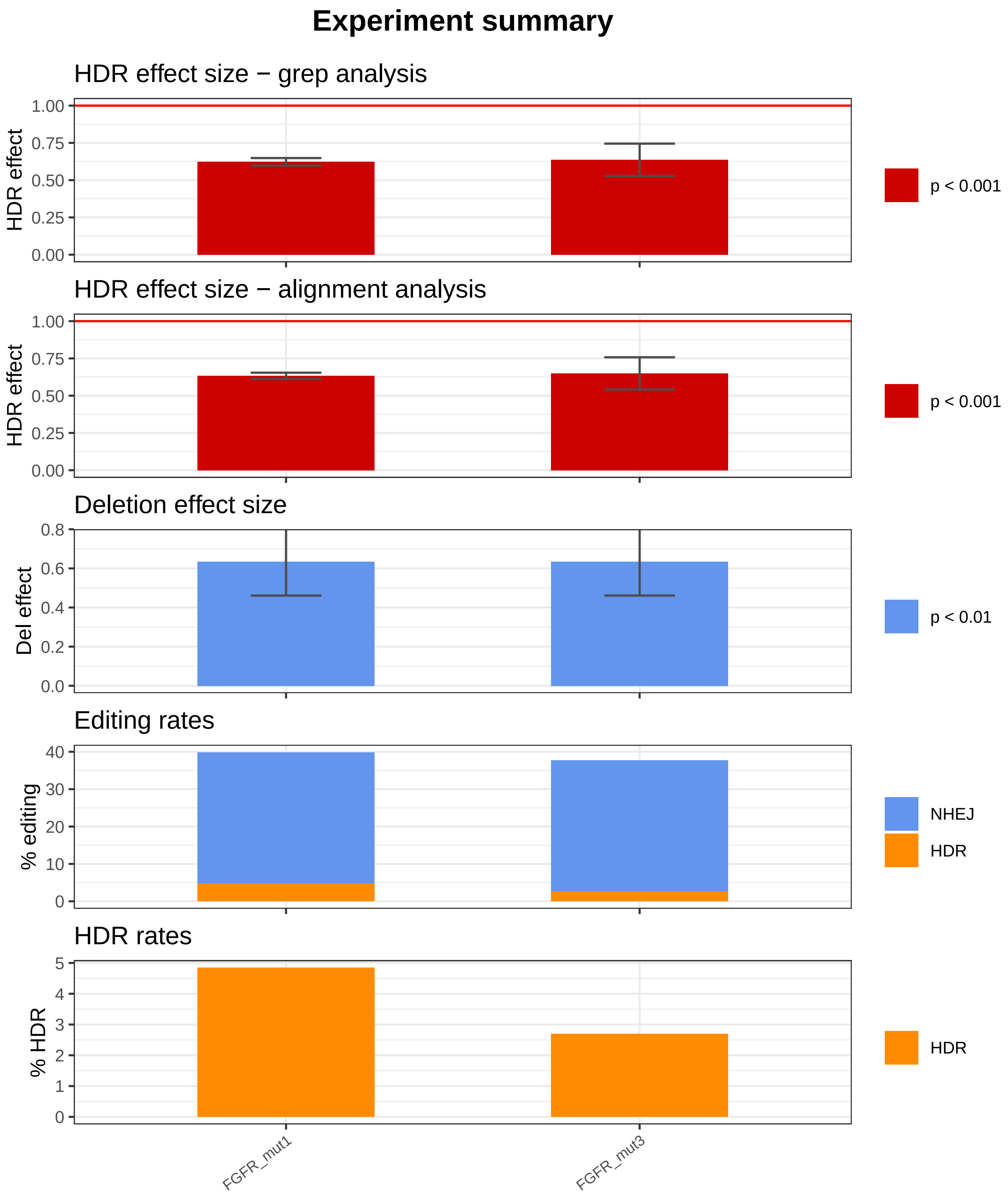

Experiment summary
HDR effect size − grep analysis
HDR effect
1.00
0.75
0.50
0.25
0.00
p < 0.001
HDR effect size − alignment analysis
HDR effect
1.00
0.75
0.50
0.25
0.00
p < 0.001
Deletion effect size
Del effect
0.8
0.6
0.4
0.2
0.0
p < 0.01
Editing rates
% editing
40
30
20
10
0
NHEJ
HDR
HDR rates
% HDR
5
4
3
2
1
0
HDR
FGFR_mut1
FGFR_mut3

FGFR_mut1 grep summary

Mean HDR frac gDNA: 5%, cDNA: 3.8%
Mean WT frac gDNA: 53%, cDNA: 65%

cDNA:gDNA ratio (HDR/WT): 0.623
95% CI: (0.597, 0.648),   p = 1.3e−14

gDNA2_3_1	gDNA2_1_2	gDNA2_2_2	gDNA2_3_2	gDNA2_1_3	gDNA2_2_3	gDNA2_3_3	cDNA2_1_1	cDNA2_2_1	cDNA2_3_1	cDNA2_1_2	cDNA2_2_2	cDNA2_3_2
gDNA	gDNA	gDNA	gDNA	gDNA	gDNA	gDNA	cDNA	cDNA	cDNA	cDNA	cDNA	cDNA
129563	88421	71282	92935	92062	118785	141527	71939	124494	114069	137371	109070	157116
6331	4367	3568	4376	4658	5959	7038	3318	4735	4241	5045	3918	5964
67873	47143	38307	49660	47863	62930	73565	64144	77104	70479	84036	67816	97061
0.0933	0.0926	0.0931	0.0881	0.0973	0.0947	0.0957	0.0517	0.0614	0.0602	0.06	0.0578	0.0614
4.89%	4.94%	5.01%	4.71%	5.06%	5.02%	4.97%	4.61%	3.80%	3.72%	3.67%	3.59%	3.80%
52.39%	53.32%	53.74%	53.44%	51.99%	52.98%	51.98%	89.16%	61.93%	61.79%	61.17%	62.18%	61.78%

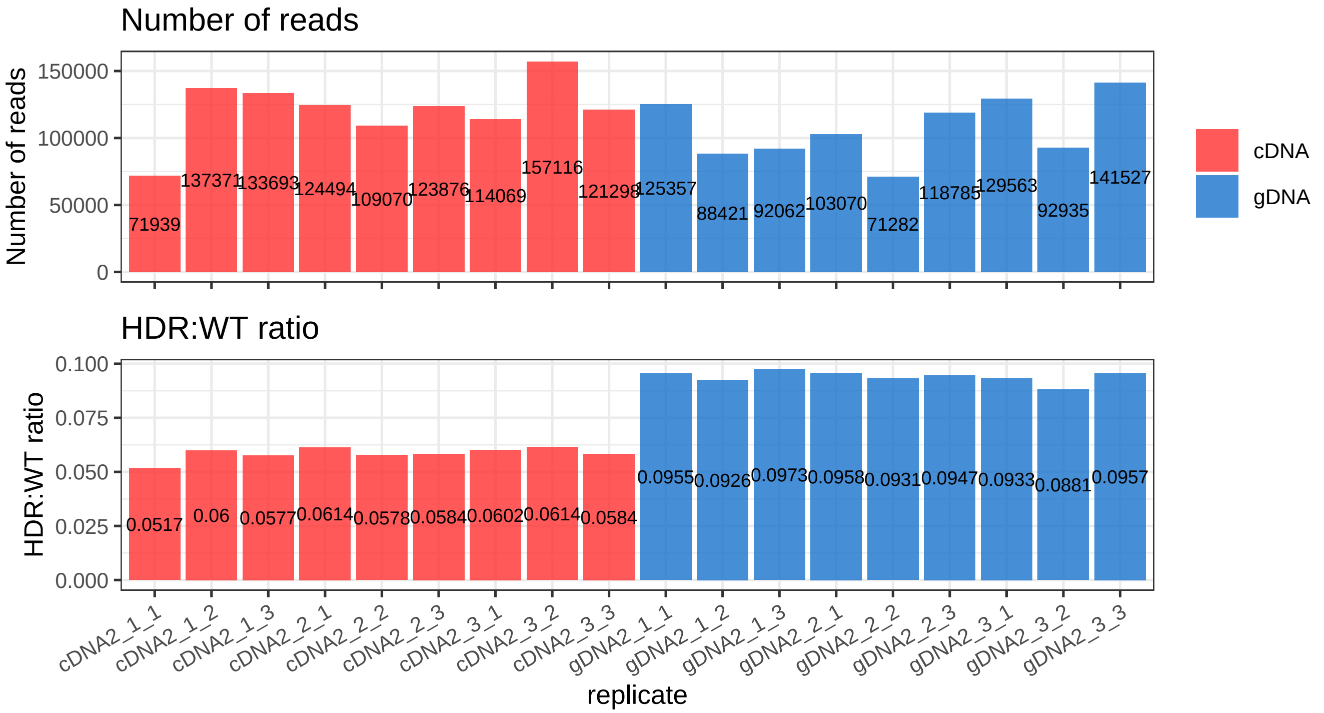

FGFR_mut1 analysis summary

Mean HDR frac gDNA: 4.8%, cDNA: 3.7%
Mean DEL frac gDNA: 35%, cDNA: 26%
Mean WT frac gDNA: 56%, cDNA: 67%

cDNA:gDNA ratio (HDR/WT): 0.633
95% CI: (0.612, 0.654), p = 1.67e−14

cDNA:gDNA ratio (DEL/WT): 0.635
95% CI: (0.461, 0.809), p = 0.00117

cDNA:gDNA ratio (DEL/WT) [109−121]: 0.634
95% CI: (0.456, 0.812), p = 0.00144

NA2_2_1	gDNA2_3_1	gDNA2_1_2	gDNA2_2_2	gDNA2_3_2	gDNA2_1_3	gDNA2_2_3	gDNA2_3_3	cDNA2_1_1	cDNA2_2_1	cDNA2_3_1	cDNA2_1_2	cDNA2_2_2	cDNA
gDNA	gDNA	gDNA	gDNA	gDNA	gDNA	gDNA	gDNA	cDNA	cDNA	cDNA	cDNA	cDNA	cD
1798	2123	1855	1540	1874	1913	1924	2186	466	1822	1698	1879	1696	19
0.0868	0.0863	0.0848	0.0851	0.0806	0.0893	0.0862	0.0889	0.0513	0.057	0.0544	0.0559	0.0532	0.05
0.615	0.639	0.608	0.589	0.611	0.656	0.618	0.65	0.0223	0.435	0.444	0.454	0.431	0.4
4.90%	4.81%	4.82%	4.88%	4.57%	4.91%	4.86%	4.92%	4.53%	3.69%	3.50%	3.58%	3.46%	3.7
4.72%	35.64%	34.54%	33.76%	34.67%	36.12%	34.84%	35.92%	1.97%	28.09%	28.60%	29.05%	28.02%	28.4
9.62%	40.45%	39.36%	38.64%	39.24%	41.03%	39.69%	40.84%	6.50%	31.78%	32.10%	32.62%	31.47%	32.1
6.43%	55.74%	56.82%	57.32%	56.77%	55.04%	56.36%	55.29%	88.23%	64.66%	64.39%	63.96%	65.02%	64.3
03070	129563	88421	71282	92935	92062	118785	141527	71939	124494	114069	137371	109070	157
4291	5297	3591	2926	3612	3879	4934	5957	2678	3504	3090	3798	2897	45
49420	61359	42370	34369	44820	43453	57246	66990	52190	61443	56826	67939	54504	785
30408	39233	25756	20246	27372	28513	35385	43523	1165	26697	25244	30854	23486	347
15213	18593	13382	11060	13583	12808	16656	19227	12729	29150	25522	30432	24892	344
0	0	0	0	0	0	1	0	0	13	0	0	1	4
62	87	71	64	81	68	104	116	47	111	116	145	116	22
220	796	400	194	318	237	446	1033	10	192	172	575	237	37
0	0	0	0	0	0	0	0	0	0	0	0	0	0

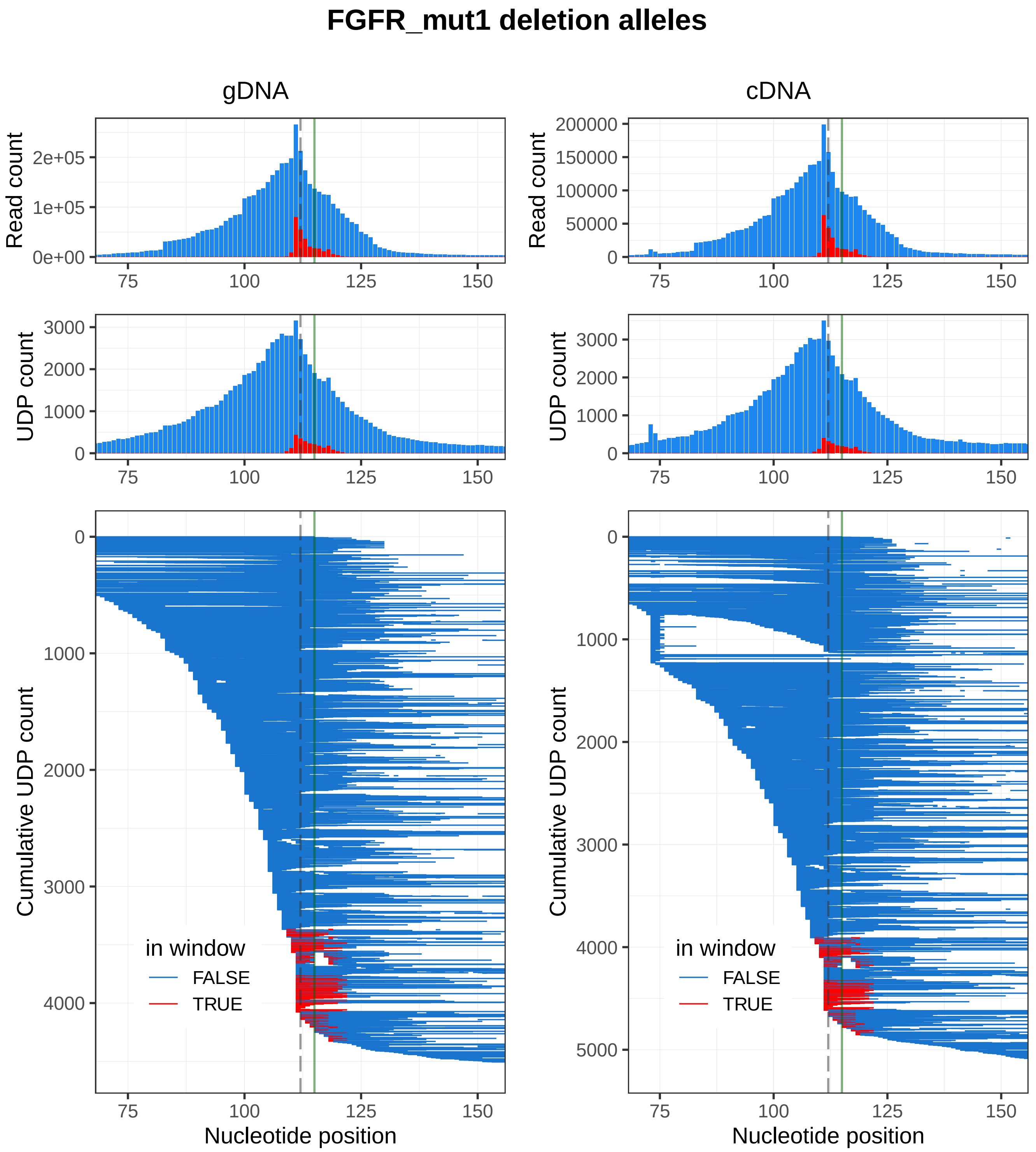
FGFR_mut1 deletion alleles
gDNA
cDNA
Read count
UDP count
Cumulative UDP count
Nucleotide position
0e+00
1e+05
2e+05
0
50000
100000
150000
200000
1000
2000
3000
4000
5000
75
100
125
150
in window
FALSE
TRUE

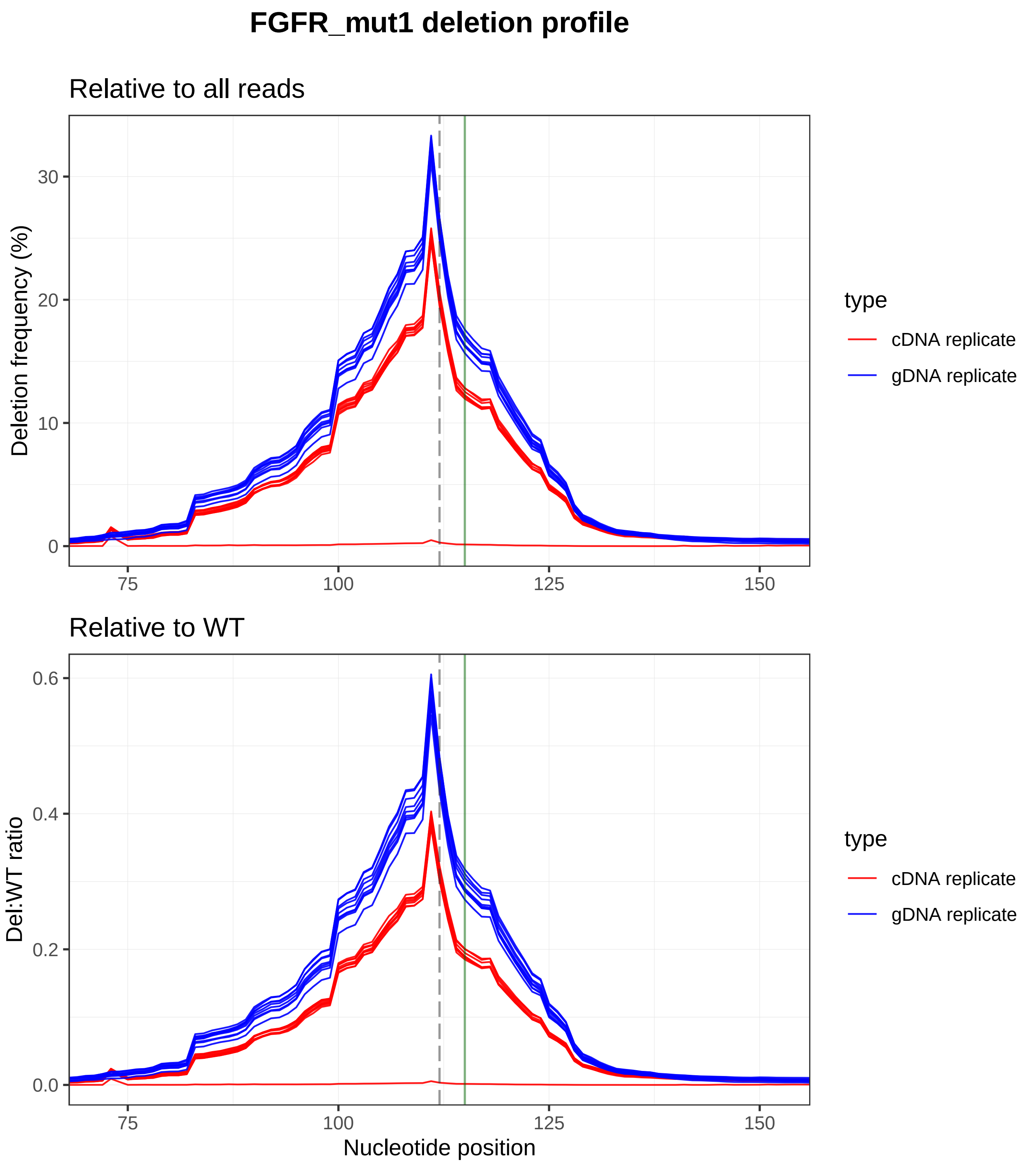
FGFR_mut1 deletion profile
Relative to all reads
Deletion frequency (%)
30
20
10
0
75
100
125
150
type
cDNA replicate
gDNA replicate
Relative to WT
Del:WT ratio
0.6
0.4
0.2
0.0
75
100
125
150
Nucleotide position
type
cDNA replicate
gDNA replicate

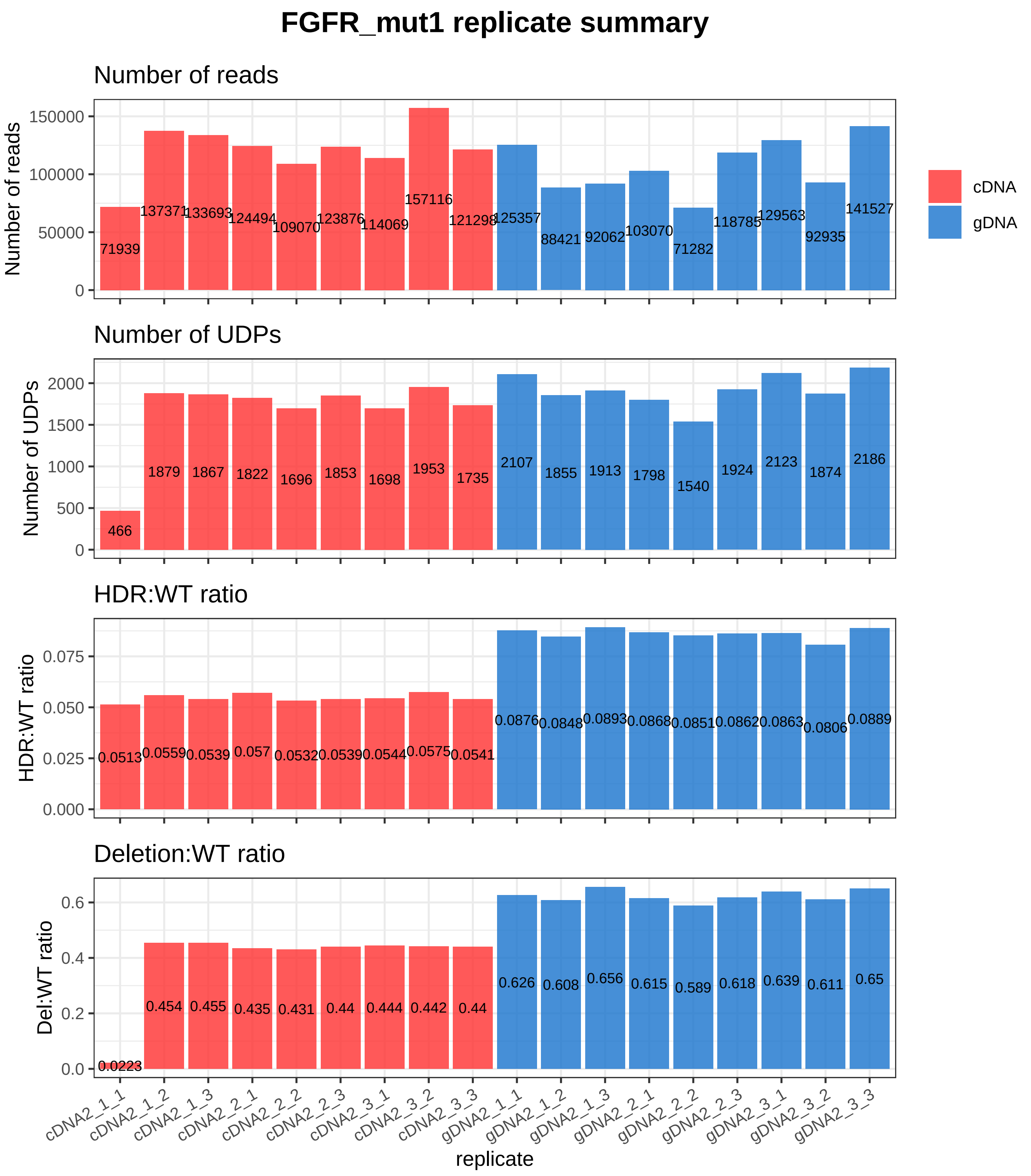
FGFR_mut1 replicate summary
Number of reads
Number of reads
150000
100000
50000
0
71939
137371
133693
124494
109070
123876
114069
157116
121298
125357
88421
92062
103070
71282
118785
129563
92935
141527
cDNA
gDNA
Number of UDPs
Number of UDPs
2000
1500
1000
500
0
466
1879
1867
1822
1696
1853
1698
1953
1735
2107
1855
1913
1798
1540
1924
2123
1874
2186
HDR:WT ratio
HDR:WT ratio
0.075
0.050
0.025
0.000
0.0513
0.0559
0.0539
0.057
0.0532
0.0539
0.0544
0.0575
0.0541
0.0876
0.0848
0.0893
0.0868
0.0851
0.0862
0.0863
0.0806
0.0889
Deletion:WT ratio
Del:WT ratio
0.6
0.4
0.2
0.0
0.0223
0.454
0.455
0.435
0.431
0.44
0.444
0.442
0.44
0.626
0.608
0.656
0.615
0.589
0.618
0.639
0.611
0.65
cDNA2_1_1
cDNA2_1_2
cDNA2_1_3
cDNA2_2_1
cDNA2_2_2
cDNA2_2_3
cDNA2_3_1
cDNA2_3_2
cDNA2_3_3
gDNA2_1_1
gDNA2_1_2
gDNA2_1_3
gDNA2_2_1
gDNA2_2_2
gDNA2_2_3
gDNA2_3_1
gDNA2_3_2
gDNA2_3_3
replicate

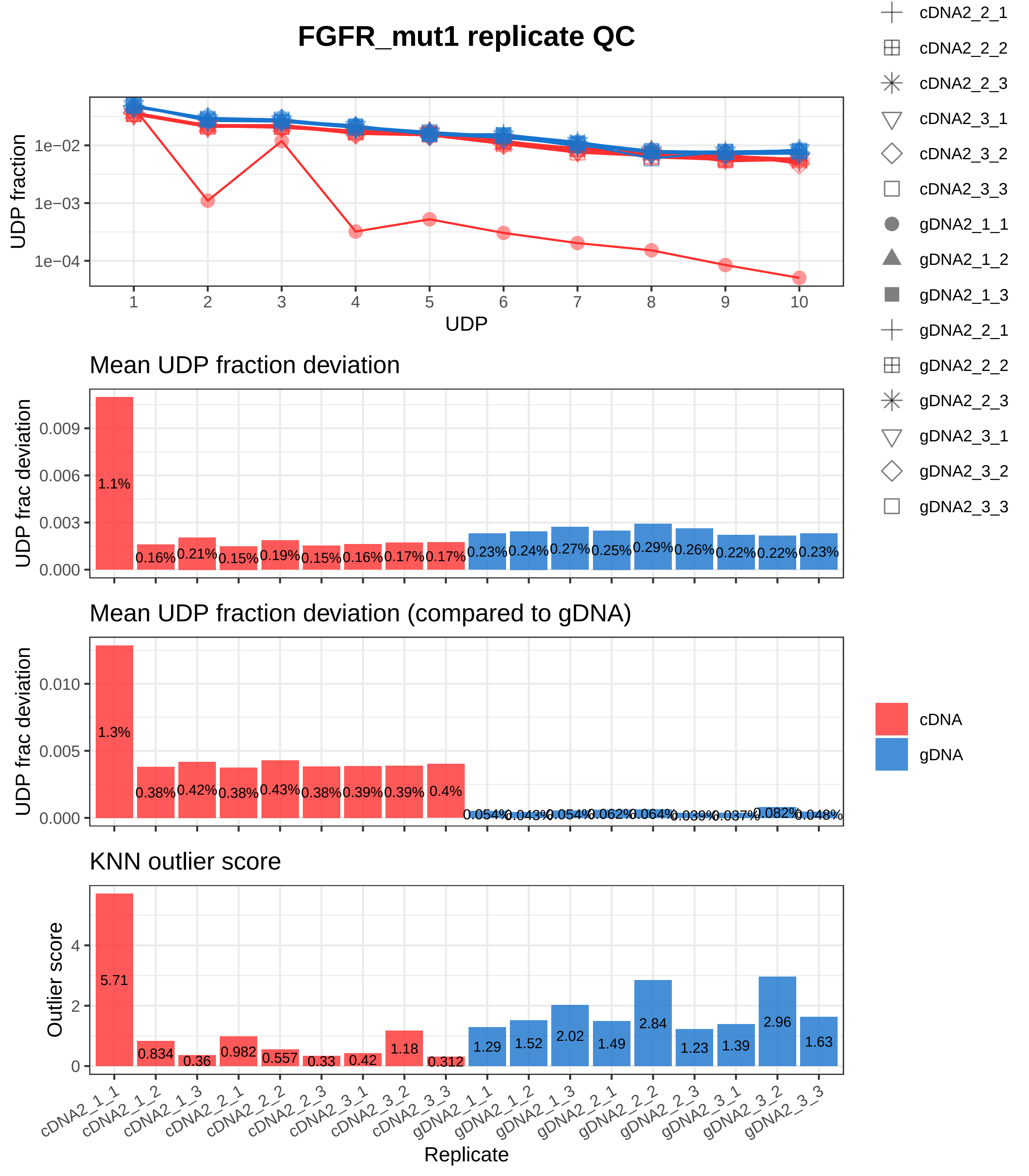

FGFR_mut1 replicate QC
UDP fraction
1e−02
1e−03
1e−04
1 2 3 4 5 6 7 8 9 10
UDP
Mean UDP fraction deviation
UDP frac deviation
0.009
0.006
0.003
0.000
1.1% 0.16% 0.21% 0.15% 0.19% 0.15% 0.16% 0.17% 0.17% 0.23% 0.24% 0.27% 0.25% 0.29% 0.26% 0.22% 0.22% 0.23%
Mean UDP fraction deviation (compared to gDNA)
UDP frac deviation
0.010
0.005
0.000
1.3% 0.38% 0.42% 0.38% 0.43% 0.38% 0.39% 0.39% 0.4% 0.054% 0.043% 0.054% 0.062% 0.064% 0.039% 0.037% 0.082% 0.048%
KNN outlier score
Outlier score
4
2
0
5.71 0.834 0.36 0.982 0.557 0.33 0.42 1.18 0.312 1.29 1.52 2.02 1.49 2.84 1.23 1.39 2.96 1.63
cDNA2_1_1 cDNA2_1_2 cDNA2_1_3 cDNA2_2_1 cDNA2_2_2 cDNA2_2_3 cDNA2_3_1 cDNA2_3_2 cDNA2_3_3 gDNA2_1_1 gDNA2_1_2 gDNA2_1_3 gDNA2_2_1 gDNA2_2_2 gDNA2_2_3 gDNA2_3_1 gDNA2_3_2 gDNA2_3_3
Replicate
cDNA2_2_1
cDNA2_2_2
cDNA2_2_3
cDNA2_3_1
cDNA2_3_2
cDNA2_3_3
gDNA2_1_1
gDNA2_1_2
gDNA2_1_3
gDNA2_2_1
gDNA2_2_2
gDNA2_2_3
gDNA2_3_1
gDNA2_3_2
gDNA2_3_3
cDNA
gDNA

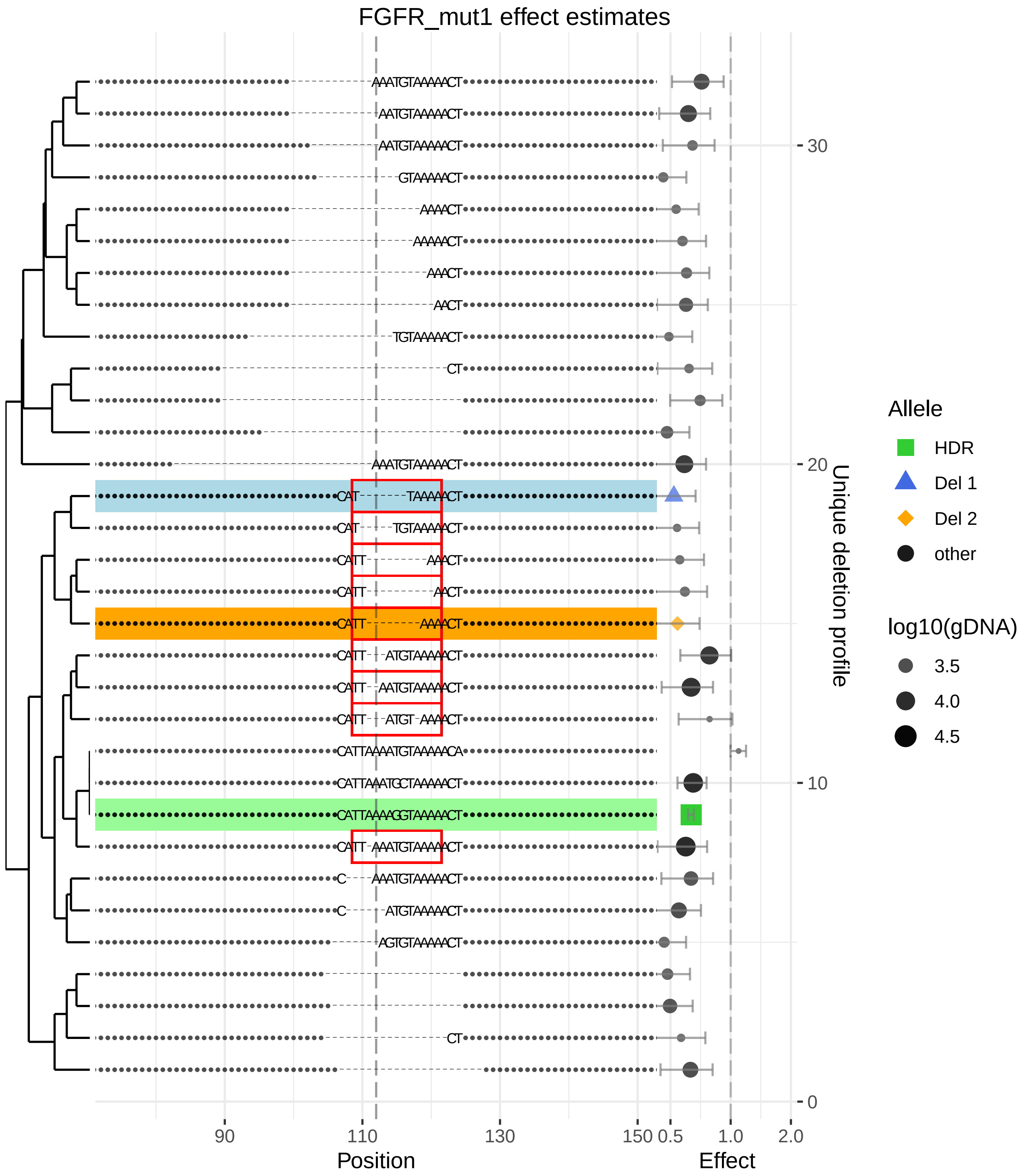
FGFR_mut1 effect estimates
AAATGTAAAAACT
AATGTAAAAACT
AATGTAAAAACT
GTAAAAACT
AAAACT
AAAAACT
AAACT
AACT
TGTAAAAACT
CT
AAATGTAAAAACT
CAT TAAAAACT
CAT TGTAAAAACT
CATT AAACT
CATT AACT
CATT AAAACT
CATT ATGTAAAAACT
CATT AATGTAAAAACT
CATT ATGT AAAACT
CATTAAAATGTAAAAACA
CATTAAATGCTAAAAACT
CATTAAAGGTAAAAACT
CATT AAATGTAAAAACT
C AAATGTAAAAACT
C ATGTAAAAACT
AGTGTAAAAACT
CT
90
110
130
150
0.5
1.0
2.0
Position
Effect
0
10
20
30
Unique deletion profile
Allele
HDR
Del 1
Del 2
other
log10(gDNA)
3.5
4.0
4.5

FGFR_mut3 grep summary

Mean HDR frac gDNA: 2.8%, cDNA: 2.1%
Mean WT frac gDNA: 53%, cDNA: 65%

cDNA:gDNA ratio (HDR/WT): 0.636
95% CI: (0.528, 0.745),   p = 4.89e−05

gDNA2_3_1	gDNA2_1_2	gDNA2_2_2	gDNA2_3_2	gDNA2_1_3	gDNA2_2_3	gDNA2_3_3	cDNA2_1_1	cDNA2_2_1	cDNA2_3_1	cDNA2_1_2	cDNA2_2_2	cDNA2_3_2
gDNA	gDNA	gDNA	gDNA	gDNA	gDNA	gDNA	cDNA	cDNA	cDNA	cDNA	cDNA	cDNA
129563	88421	71282	92935	92062	118785	141527	71939	124494	114069	137371	109070	157116
3492	2508	2107	2722	2608	3367	3821	880	2912	2544	3040	2494	3535
67711	47037	38237	49564	47759	62813	73393	64040	76905	70307	83824	67658	96785
0.0516	0.0533	0.0551	0.0549	0.0546	0.0536	0.0521	0.0137	0.0379	0.0362	0.0363	0.0369	0.0365
2.70%	2.84%	2.96%	2.93%	2.83%	2.83%	2.70%	1.22%	2.34%	2.23%	2.21%	2.29%	2.25%
52.26%	53.20%	53.64%	53.33%	51.88%	52.88%	51.86%	89.02%	61.77%	61.64%	61.02%	62.03%	61.60%

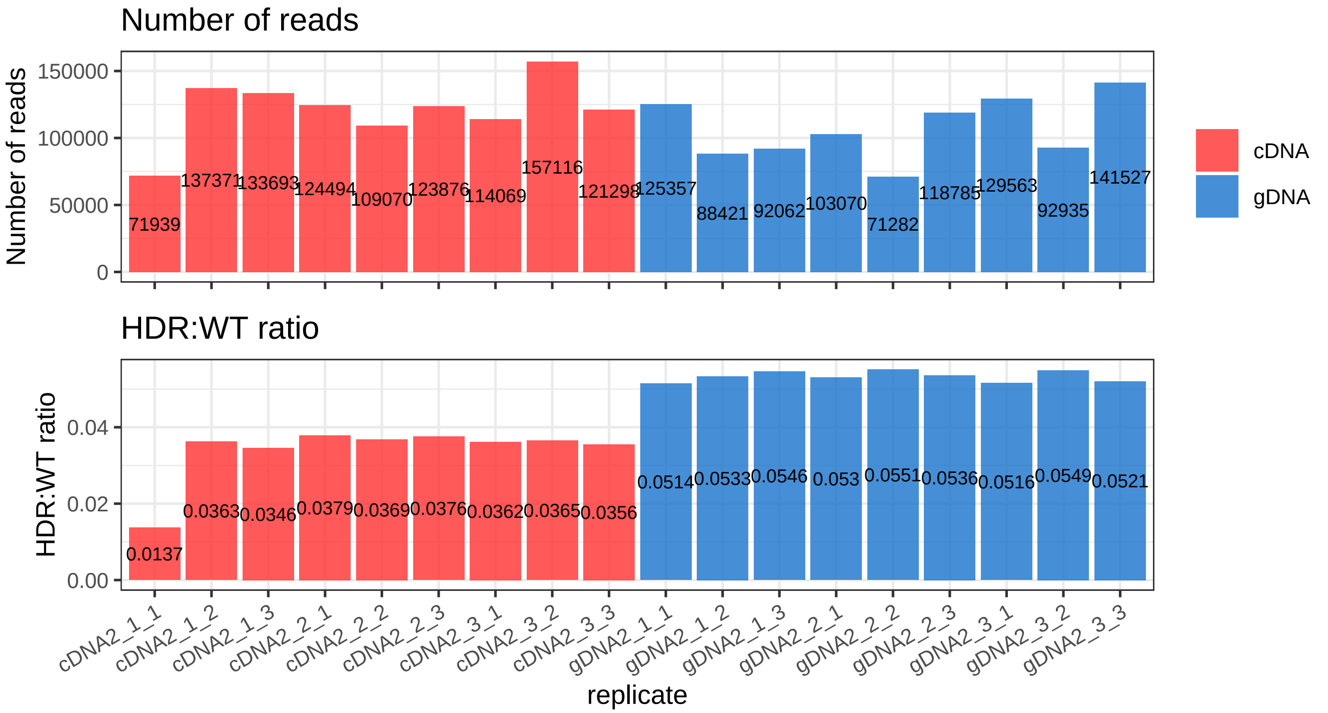

FGFR_mut3 analysis summary

Mean HDR frac gDNA: 2.7%, cDNA: 2%
Mean DEL frac gDNA: 35%, cDNA: 26%
Mean WT frac gDNA: 56%, cDNA: 67%

cDNA:gDNA ratio (HDR/WT): 0.65
95% CI: (0.542, 0.758),   p = 5.53e−05

cDNA:gDNA ratio (DEL/WT): 0.635
95% CI: (0.461, 0.809),   p = 0.00118

cDNA:gDNA ratio (DEL/WT) [109−121]: 0.634
95% CI: (0.456, 0.812),   p = 0.00145

NA2_2_1	gDNA2_3_1	gDNA2_1_2	gDNA2_2_2	gDNA2_3_2	gDNA2_1_3	gDNA2_2_3	gDNA2_3_3	cDNA2_1_1	cDNA2_2_1	cDNA2_3_1	cDNA2_1_2	cDNA2_2_2	cDNA
gDNA	gDNA	gDNA	gDNA	gDNA	gDNA	gDNA	gDNA	cDNA	cDNA	cDNA	cDNA	cDNA	cD
1866	2217	1926	1593	1942	1966	2004	2271	491	1890	1749	1945	1761	20
.0484	0.0467	0.0478	0.0491	0.0495	0.0487	0.0482	0.0468	0.0135	0.0346	0.0333	0.0337	0.0332	0.03
.616	0.641	0.609	0.59	0.612	0.657	0.619	0.651	0.0224	0.435	0.445	0.455	0.432	0.4
.73%	2.60%	2.71%	2.81%	2.80%	2.68%	2.71%	2.58%	1.19%	2.23%	2.14%	2.15%	2.15%	2.1
4.72%	35.64%	34.54%	33.76%	34.67%	36.11%	34.83%	35.92%	1.97%	28.09%	28.60%	29.05%	28.02%	28.4
7.45%	38.23%	37.25%	36.57%	37.47%	38.79%	37.55%	38.50%	3.16%	30.33%	30.74%	31.20%	30.17%	30.6
6.33%	55.64%	56.74%	57.22%	56.68%	54.95%	56.27%	55.20%	88.12%	64.52%	64.26%	63.82%	64.91%	64.2
03070	129563	88421	71282	92935	92062	118785	141527	71939	124494	114069	137371	109070	157
2389	2858	2024	1686	2214	2114	2758	3130	705	2122	1886	2287	1804	26
49335	61249	42310	34313	44749	43389	57161	66877	52124	61311	56721	67795	54414	784
30408	39233	25756	20246	27372	28513	35385	43523	1165	26697	25244	30854	23486	347
15213	18593	13382	11060	13583	12808	16656	19227	12729	29150	25522	30432	24892	344
0	0	0	0	0	0	1	0	0	13	0	0	1	4
62	84	65	58	78	63	96	105	47	110	110	138	116	22
220	796	400	194	318	237	446	1033	10	192	172	575	237	37
0	0	0	0	0	0	0	0	0	0	0	0	0	0

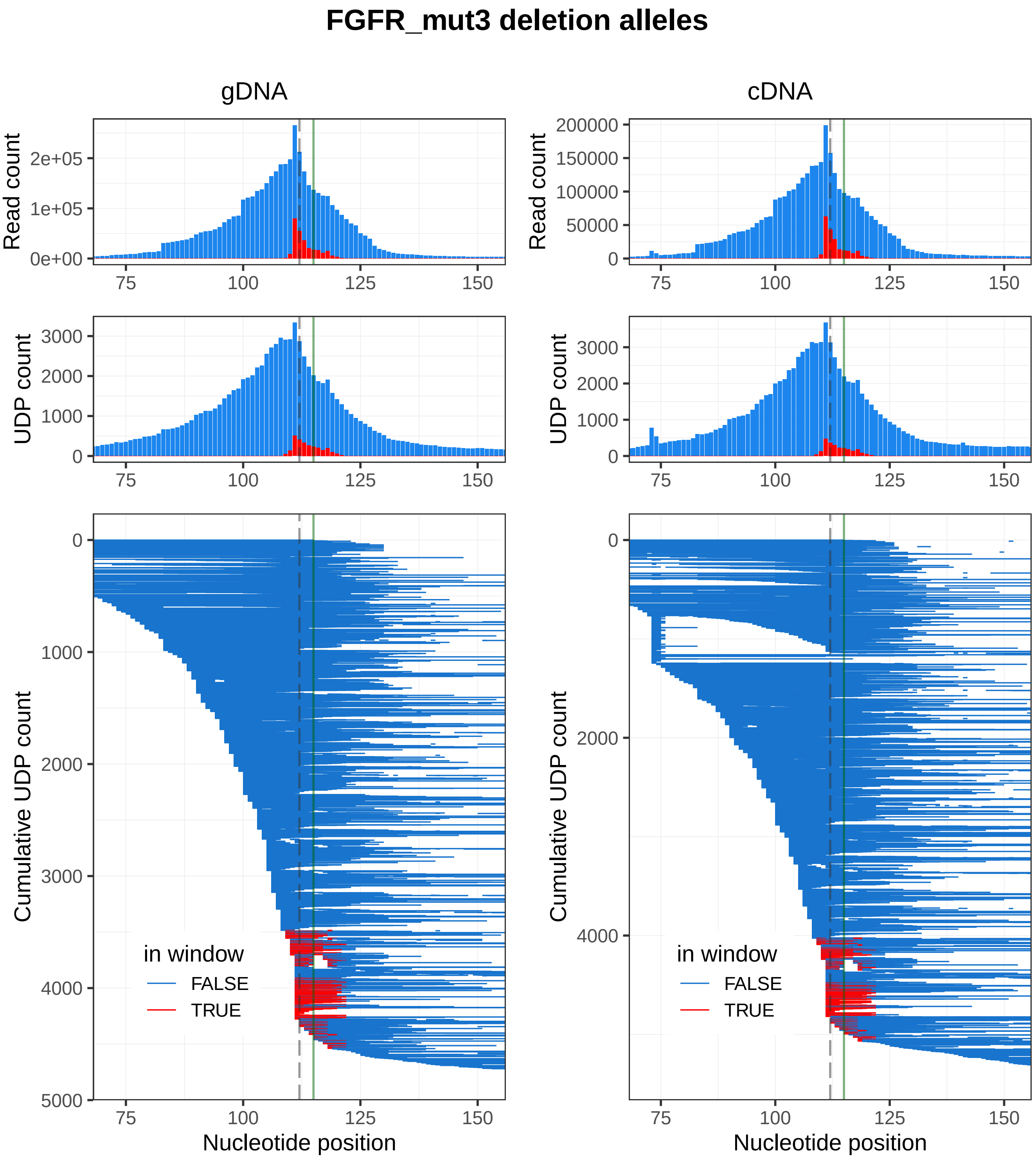
FGFR_mut3 deletion alleles
gDNA
cDNA
Read count
UDP count
Cumulative UDP count
Nucleotide position
in window
FALSE
TRUE
0e+00
1e+05
2e+05
200000
150000
100000
50000
0
3000
2000
1000
4000
5000
75
100
125
150

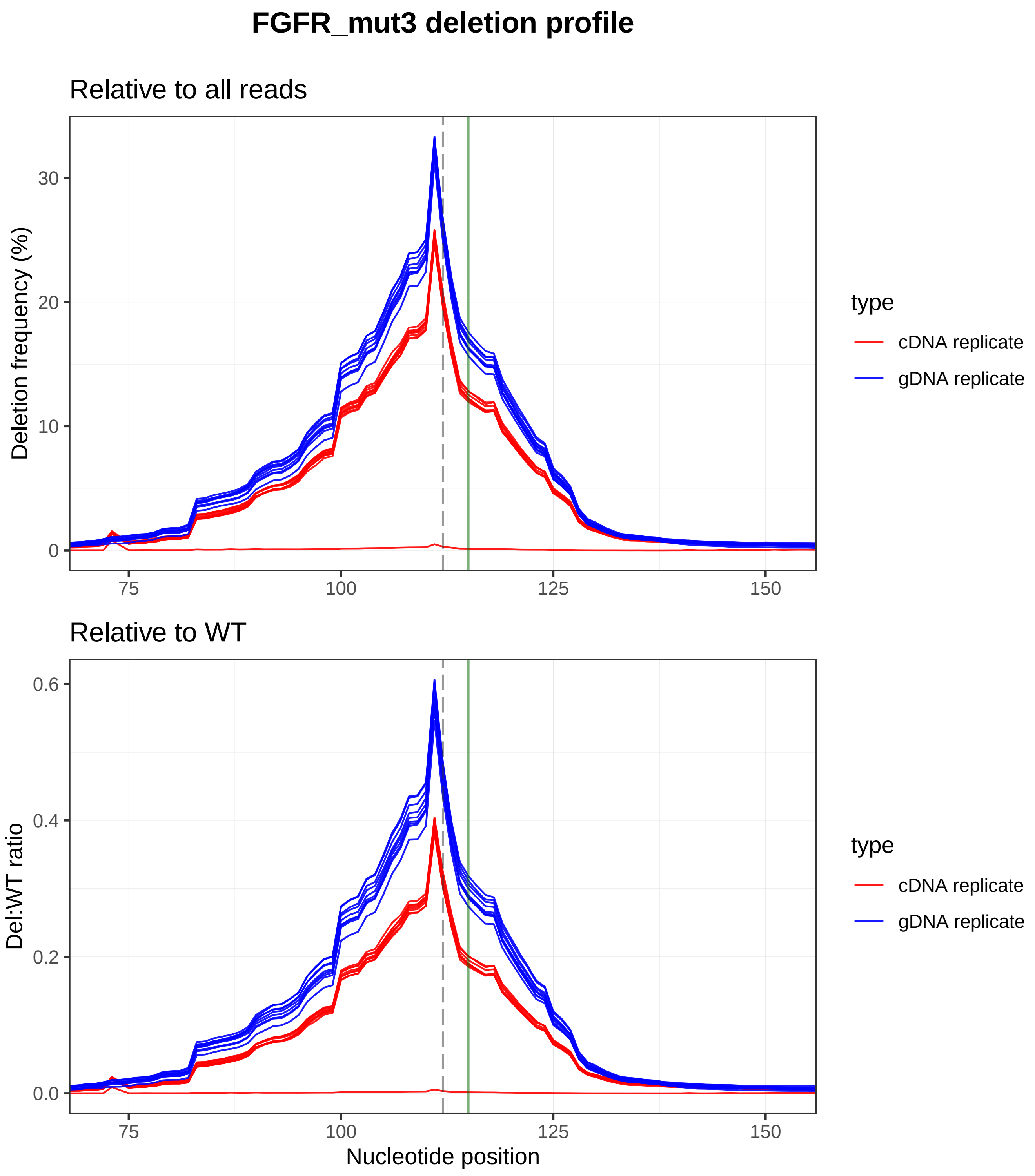
FGFR_mut3 deletion profile
Relative to all reads
Deletion frequency (%)
30
20
10
0
75
100
125
150
type
cDNA replicate
gDNA replicate
Relative to WT
Del:WT ratio
0.6
0.4
0.2
0.0
75
100
125
150
Nucleotide position
type
cDNA replicate
gDNA replicate

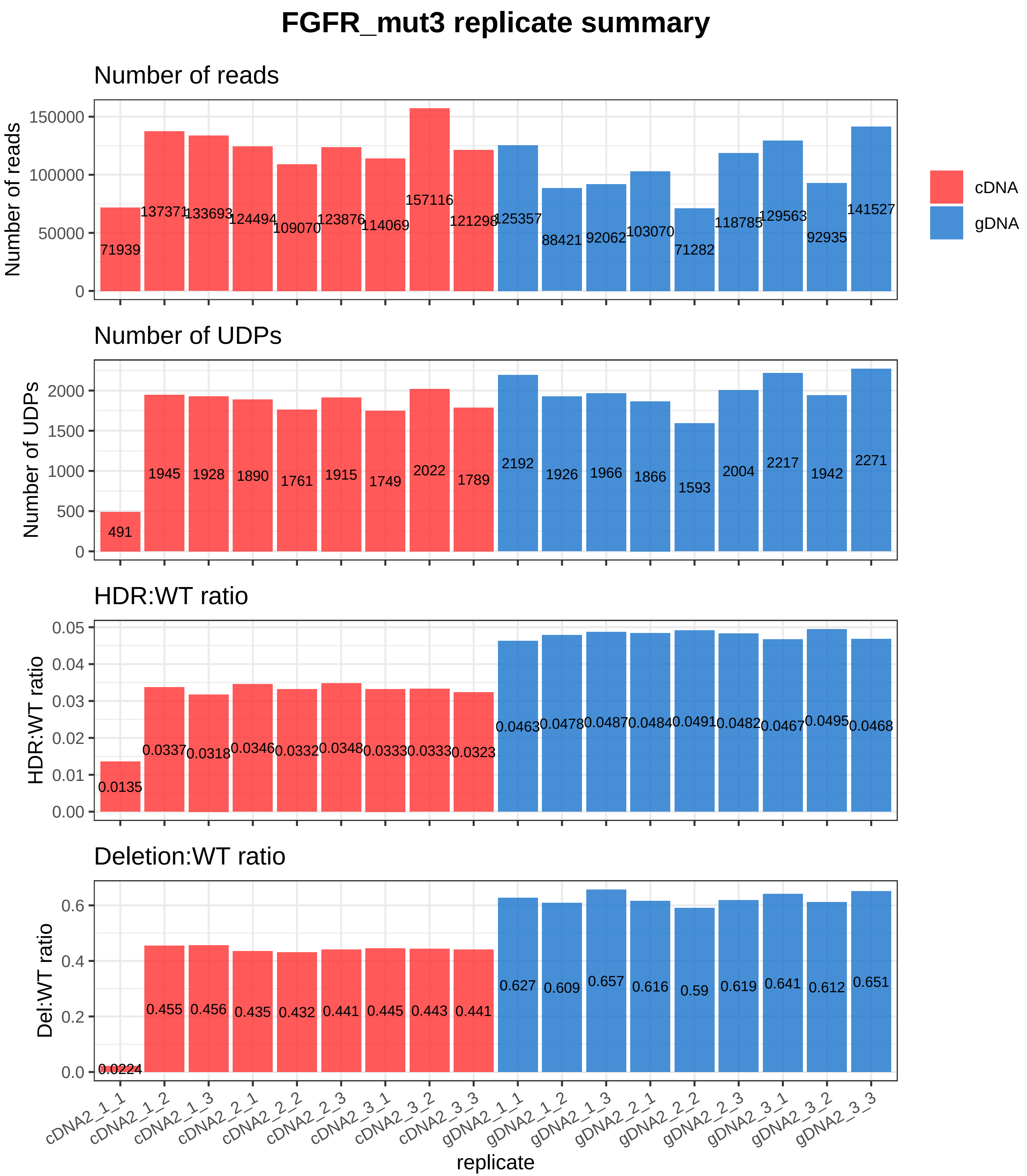
FGFR_mut3 replicate summary
Number of reads
Number of reads
150000
100000
50000
0
71939
137371
133693
124494
109070
123876
114069
157116
121298
125357
88421
92062
103070
71282
118785
129563
92935
141527
Number of UDPs
Number of UDPs
2000
1500
1000
500
0
491
1945
1928
1890
1761
1915
1749
2022
1789
2192
1926
1966
1866
1593
2004
2217
1942
2271
HDR:WT ratio
HDR:WT ratio
0.05
0.04
0.03
0.02
0.01
0.00
0.0135
0.0337
0.0318
0.0346
0.0332
0.0348
0.0333
0.0333
0.0323
0.0463
0.0478
0.0487
0.0484
0.0491
0.0482
0.0467
0.0495
0.0468
Deletion:WT ratio
Del:WT ratio
0.6
0.4
0.2
0.0
0.0224
0.455
0.456
0.435
0.432
0.441
0.445
0.443
0.441
0.627
0.609
0.657
0.616
0.59
0.619
0.641
0.612
0.651
cDNA2_1_1
cDNA2_1_2
cDNA2_1_3
cDNA2_2_1
cDNA2_2_2
cDNA2_2_3
cDNA2_3_1
cDNA2_3_2
cDNA2_3_3
gDNA2_1_1
gDNA2_1_2
gDNA2_1_3
gDNA2_2_1
gDNA2_2_2
gDNA2_2_3
gDNA2_3_1
gDNA2_3_2
gDNA2_3_3
replicate
cDNA
gDNA

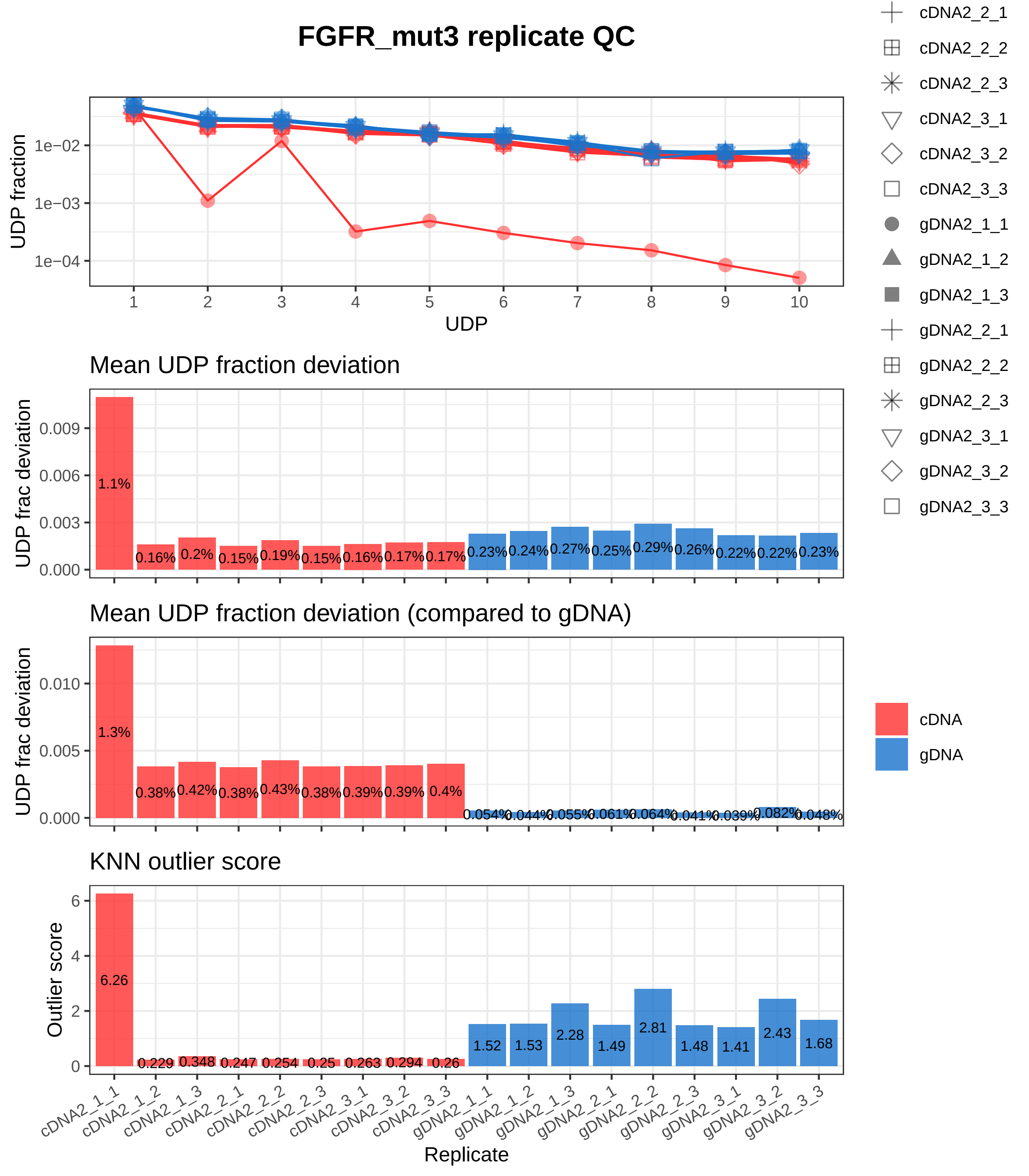

FGFR_mut3 replicate QC
UDP fraction
1e−02
1e−03
1e−04
UDP
1 2 3 4 5 6 7 8 9 10
cDNA2_2_1
cDNA2_2_2
cDNA2_2_3
cDNA2_3_1
cDNA2_3_2
cDNA2_3_3
gDNA2_1_1
gDNA2_1_2
gDNA2_1_3
gDNA2_2_1
gDNA2_2_2
gDNA2_2_3
gDNA2_3_1
gDNA2_3_2
gDNA2_3_3
Mean UDP fraction deviation
UDP frac deviation
0.009
0.006
0.003
0.000
1.1%
0.16%
0.2%
0.15%
0.19%
0.15%
0.16%
0.17%
0.17%
0.23%
0.24%
0.27%
0.25%
0.29%
0.26%
0.22%
0.22%
0.23%
Mean UDP fraction deviation (compared to gDNA)
UDP frac deviation
0.010
0.005
0.000
1.3%
0.38%
0.42%
0.38%
0.43%
0.38%
0.39%
0.39%
0.4%
0.054%
0.044%
0.055%
0.061%
0.064%
0.041%
0.039%
0.082%
0.048%
cDNA
gDNA
KNN outlier score
Outlier score
6
4
2
0
6.26
0.229
0.348
0.247
0.254
0.25
0.263
0.294
0.26
1.52
1.53
2.28
1.49
2.81
1.48
1.41
2.43
1.68
cDNA2_1_1
cDNA2_1_2
cDNA2_1_3
cDNA2_2_1
cDNA2_2_2
cDNA2_2_3
cDNA2_3_1
cDNA2_3_2
cDNA2_3_3
gDNA2_1_1
gDNA2_1_2
gDNA2_1_3
gDNA2_2_1
gDNA2_2_2
gDNA2_2_3
gDNA2_3_1
gDNA2_3_2
gDNA2_3_3
Replicate

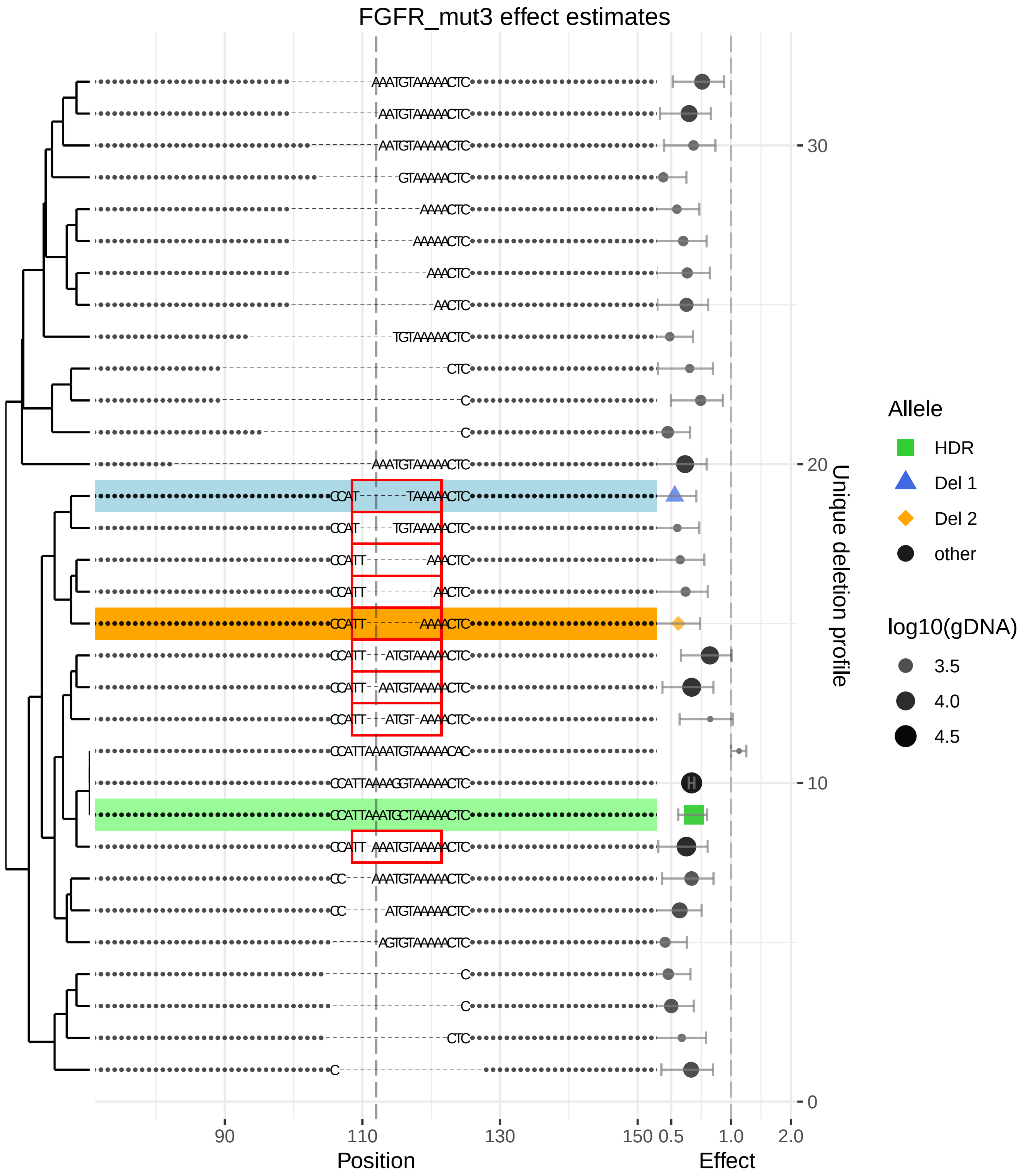
FGFR_mut3 effect estimates
AAATGTAAAAACTC
AATGTAAAAACTC
AATGTAAAAACTC
GTAAAAACTC
AAAACTC
AAAAACTC
AAACTC
AACTC
TGTAAAAACTC
CTC
C
C
AAATGTAAAAACTC
CCAT TAAAAACTC
CCAT TGTAAAAACTC
CCATT AAACTC
CCATT AACTC
CCATT AAAACTC
CCATT ATGTAAAAACTC
CCATT AATGTAAAAACTC
CCATT ATGT AAAACTC
CCATTAAAATGTAAAAACAC
CCATTAAAAGGTAAAAACTC
CCATTAAATGCTAAAAACTC
CCATT AAATGTAAAAACTC
CC AAATGTAAAAACTC
CC ATGTAAAAACTC
AGTGTAAAAACTC
C
C
CTC
C
90
110
130
150
0.5
1.0
2.0
Position
Effect
0
10
20
30
Unique deletion profile
Allele
HDR
Del 1
Del 2
other
log10(gDNA)
3.5
4.0
4.5
